# Easy access to oxygenated block polymers via switchable catalysis

**DOI:** 10.1038/s41467-019-10481-w

**Published:** 2019-06-17

**Authors:** Tim Stößer, Gregory S. Sulley, Georgina L. Gregory, Charlotte K. Williams

**Affiliations:** Department of Chemistry, University of Oxford, Chemistry Research Laboratory, 12 Mansfield Road, Oxford, OX1 3TA UK

**Keywords:** Catalytic mechanisms, Polymer synthesis

## Abstract

Oxygenated block polyols are versatile, potentially bio-based and/or degradable materials widely applied in the manufacture of coatings, resins, polyurethanes and other products. Typical preparations involve multistep syntheses and/or macroinitiator approaches. Here, a straightforward and well-controlled one-pot synthesis of ABA triblocks, namely poly(ether-*b*-ester-*b*-ether), and ABCBA pentablocks, of the form poly(ester-*b*-ether-*b*-ester’-*b*-ether-*b*-ester), using a commercial chromium catalyst system is described. The polymerization catalysis exploits mechanistic switches between anhydride/epoxide ring-opening copolymerization, epoxide ring-opening polymerization and lactone ring-opening polymerization without requiring any external stimuli. Testing a range of anhydrides, epoxides and chain-transfer agents reveals some of the requirements and guidelines for successful catalysis. Following these rules of switch catalysis with multiple monomer additions allows the preparation of multiblock polymers of the form (ABA)_n_ up to 15 blocks. Overall, this switchable catalysis delivers polyols in a straightforward and highly controlled manner. As proof of potential for the materials, methods to post-functionalize and/or couple the polyols to make higher polymers are demonstrated.

## Abstract (web summary)

Multiblock oxygenated polyols often show better properties than the constituent polyols, but their synthesis can be complex and difficult. Here a switchable catalysis concept is described which allows for the efficient preparation of multiblock poly(ether-*b*-ester) materials starting from mixtures of common monomers.

## Introduction

Polyethers are important commercial products for uses including lubricants, sealants, coatings, adhesives and are experiencing a renaissance due to new applications in energy storage, personal care and medicine^1–5^. Polyether polyols are especially useful as low molar mass, hydroxyl-terminated chains integral to polyurethane manufacturing. For example, polyether polyols are key to making the flex-foams used in many furnishings, automotive interiors, house hold goods and clothing. By changing their chemistry, polyether polyols are essential to make rigid foams for home insulation and construction^6,7^. Polyols fall into three major chemistries: polyether, polyester and polycarbonate and are widely used to make a myriad of higher polymers and networks such as polyurethanes, polyesters and polyamides. Overall material performance depends on many factors but it is important that polyols show properties such as: (i) Low molar masses, typically 400 < *M*_n_ < 5,000 g mol^−1^; (ii) Controllable hydroxyl end-group functionality; and (iii) Predictable structural features, such as soft vs. hard components, to moderate thermal-mechanical behaviour^6^. Polyether polyols are the largest volume polyols and are beneficial to improve product flexibility and hydrolytic stability. Polyester polyols are the second largest sub-sector and are applied to improve material strength and solvent resistance^6,8^. Combining both sets of properties in a single polyol would be highly desirable particularly because blends are often incompatible driving phase separation^9^. Block polyols would be attractive either to stabilise blends or as materials in their own right, but their preparation requires controlled polymerization methods.

Most polyethers are synthesised by epoxide ring-opening polymerization, e.g. polypropene oxide. A few polyesters are also made by lactone ROP, e.g. polycaprolactone, but most require condensation polymerizations of diacids and diols. Since the latter route lacks control it prevents generalised syntheses of the widest range of block polymers. In the academic literature selected aliphatic poly(ether-*b*-esters) are made using ROP of first an epoxide and second, after complete epoxide conversion, by lactone addition^10,11^. The approach is interesting but is fundamentally limited by the range of available and polymerizable lactones and, for some of the better catalysts, by the need for a time-sensitive acid addition to prevent undesirable side-reactions^10,11^.

An attractive alternative would be processes applicable to the broadest range of polymer structures which start from monomer mixtures to obviate careful reaction monitoring and timed reagent additions. Recently a number of groups have exploited external switch processes, such as redox or photochemical changes to the catalyst, to control selectivity from monomer mixtures^12–16^. The catalyst switching strategy is particularly successful when applied to lactone or cyclic carbonate ROP^17–23^, but because epoxide ROP catalysts are highly nucleophilic they are less compatible with polyester blocks.

Another controlled polymerization route to polyesters is the ring-opening copolymerization of epoxides and anhydrides (ROCOP). Its advantages include applicabilty to the wide range of commercial epoxides/anhydrides, many of which are industrially relevant, and a broad polymer scope. Importantly, ROCOP efficiently produces a much broader range of polyesters than ROP, including repeat units that are aromatic, rigid, functionalized and/or aliphatic. One draw-back is that some ROCOP catalysts randomly incorporate ether linkages through sequential epoxide ring-opening reactions and selectivity is exacerbated by significant rate accelerations for reactions conducted in neat epoxide (the rate law is frequently first order in epoxide concentration)^24,25^. There are only two types of switchable processes yielding poly(ester-*b*-ethers) from monomer mixtures. The first are a series of redox-switchable ROP catalysts yielding poly(cyclohexene oxide-*b*-lactide) which were reported independently by the teams of Diaconescu and Byers^12,16,26^. The second is a manganese corrole ROCOP catalyst that slowly produced poly(propylene glutarate-*b*-propylene oxide) which was reported by Nozaki and co-workers^27^.

Our research group have reported a different form of switchable cataysis where orthogonal metal-chain end-group reactivity enables high monomer selectivity and provides a route to switch between different polymerizations^17–19,21,23,28^. Various di-zinc catalysts can be switched between lactone ring-opening polymerizations and epoxide/CO_2_/anhydride ring-opening copolymerizations to make di- or triblock polymers^17–19,21^. Recently we showed that Cr(III) or Al(III) catalyst systems follow the same mechanism and yielded block polyesters^23,28^. Other researchers have successfully applied the same switchable polymerization method to di-zinc^20^, di-aluminium^29^ and transition metal^30^ catalysts. A requirement for successful catalysis is that the ROCOP cycles yield highly alternating polyesters, i.e. the catalysts are selective for epoxide alternating with anhydride enchainment. One consequence is that none of the previously reported systems show any appreciable epoxide homopolymerization and thus blocks featuring polyether linkages are inaccessible. Here, we overcome this deficiency and describe catalytic processes delivering block and multiblock polymers featuring ether and ester linkages from monomer mixtures.

## Results

### Selective polymerization of epoxide and anhydride mixtures

**Fig. 1:**
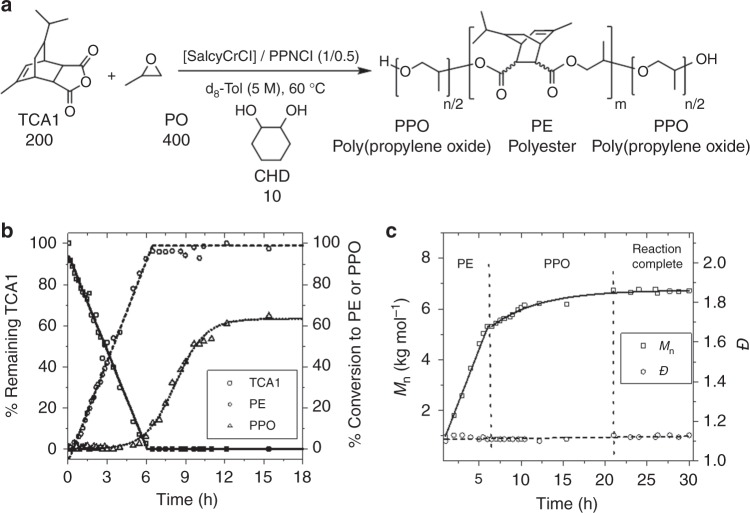
Switch catalysis using TCA1/PO. **a** Switch catalysis reaction scheme. Conditions: [SalcyCrCl]/[PPNCl]/[CHD]/TCA1/PO = 1:0.5:10:200:400, Tol-d_8_ (5 M), 60 °C; **b** normalised polymerization conversion vs. time plots for the reaction of TCA1/PO (Supplementary Figs. 1 and 2, Supplementary Table 1. The maximum conversion was limited to 65% due to monomer loss at a high number of aliquots) and **c** evolution of *M*_n_ and *Ð* (relative to PS standards; Supplementary Figs. 6 and 7, Supplementary Table 2)

The catalyst was a Cr(III) salen complex, [SalcyCrCl], applied with a co-catalyst (bis(triphenylphosphoranylidene)ammonium chloride, PPNCl) (Fig. 1). It was selected on the basis of its commercial availability and successful track record as an epoxide/anhydride ROCOP catalyst^31–33^. In these prior ROCOP studies the formation of ether linkages or blocks was not observed but the reactions were conducted with equivalent epoxide:anhydride loadings which would prevent observation of epoxide ROP following the alternating enchainment. Additionally, related bimetallic Cr-salen complexes were effective stereoselective propene oxide ROP catalysts, suggesting that under proper conditions the catalysts might be able to enchain polyether blocks^34,35^. It is also worth commenting that previously, [SalcyCrCl] was used in switchable polymerizations of cyclohexene oxide (CHO)/ anhydrides/decalactone to selectively produce poly(ester-*b*-ester’) without any evidence of ether blocks or linkages. This lack of cyclohexene oxide ROP, even when the epoxide was applied in excess, is proposed to be due to the higher relative barrier to cyclohexene ROP compared to propene oxide^36^.

As a starting point in the current investigations, the one-pot polymerization of a tricyclic anhydride (TCA1: available by the Diels-Alder reaction between maleic anhydride and α-phellandrene)^37^ and excess of propene oxide (PO) was investigated. The polymerization was conducted using a small excess of alcohol vs. catalyst which functions as chain-transfer agent (CTA; typically 10 equiv.). Adding alcohols to these polymerizations causes fast and reversible equilibration with the propagating metal alkoxide intermediate resulting in controlled polymerization^38–40^. Initially, 1,2-*trans*-cyclohexane diol (CHD) was selected to allow for chain-growth simultaneously on both sides of the CTA, thereby yielding symmetric polyols.

To investigate whether both monomers were incorporated randomly or if well-defined block-sequences were obtained, the reaction was monitored by both regular aliquot removal and analysis by ^1^H NMR spectroscopy and GPC (Fig. 1, Supplementary Fig. 1). The reaction occurred in two distinct stages: firstly by anhydride/epoxide ROCOP and, after complete anhydride consumption, with the excess PO undergoing ROP. The conversion-time data illustrate the high selectivity for epoxide/anhydride ROCOP over epoxide ROP and, in particular, only traces of polyether were detected during the first reaction stage (at 6.5 h: >95% alternating polyester, PE, from ROCOP and <5% polypropene oxide, PPO; NMR spectra shown in Supplementary Fig. 2 and MALDI in Supplementary Fig. 3). Once the ROCOP stage was complete, the ring-opening polymerization of PO yielded a polyether block until all the epoxide was depleted (Fig. 1b).

The polymerization kinetic profiles were analysed by applying fits to the conversion vs. time data. This suggests that the ROCOP polyester formation follows a zeroth order rate dependence in anhydride concentration (*k*_obs,ROCOP_ = 0.78 ± 0.02 M^−1^ h^−1^, Supplementary Fig. 4) and implies that the catalyst resting state is a chromium carboxylate intermediate formed by rapid TCA1 insertion. The ROP step shows a first order rate dependence in propene oxide concentration (*k*_obs,ROP_ = 0.15 ± 0.01 h^−1^, Supplementary Fig. 5). Although the high selectivity for PO/TCA1 ROCOP followed by PO ROP is clearly indicated by the data, the precise polymer composition is not yet known. In fact different polymer structures could all be consistent with the spectroscopy, including the desired block polymer (PPO-*b*-PE-*b*-PPO) but not ruling out mixtures of homopolymers (PE + PPO) or a scrambled block structure. To examine the polymer structure, the reaction aliquots were analysed by GPC as the reaction progressed (Fig. 1c and Supplementary Figs. 6, 7). The polymer molar mass increases continuously throughout the reaction and the dispersity remains narrow for all samples (*Ð*~1.1). Plotting the molar mass data vs. time shows a linear increase during the epoxide/anhydride ROCOP stage and an exponential increase during the epoxide ROP stage (Supplementary Table 2). The fits are in line with the kinetic data obtained from NMR spectroscopy. The molar mass data and fits suggest the product is a block polymer rather than a polymer mixture because the latter outcome should result in at least two molar mass distributions. Indeed, the expected bimodal molar mass distribution was observed when the GPC traces for individually synthesised PE and PPO were overlaid (Supplementary Fig. 8). The difference between the switchable catalyst and these control experiments provides good support for the formation of block polymers. The polymerization was also allowed to maintain contact with the catalyst system for 12 h after reaction completion and the molar mass distribution did not broaden. These findings are consistent with minimal transesterification side-reactions occurring under these conditions (Fig. 1c)^41^. The formation of a block polymer was also indicated by product analysis using DOSY NMR spectroscopy (Supplementary Fig. 9). The reaction product showed a single diffusion coefficient but a blend of the analogous polyester and polyether (of similar molar masses to the constituent blocks) showed two different diffusion rates. Moreover, only signals for polyether end-groups were observed by ^1^H NMR spectroscopy, as confirmed by comparison against the signals for pure PPO and pure PE (Supplementary Fig. 10). Overall the formation of a block polymer is indicated by all the analytical techniques and spectroscopies and it is an ABA type poly(ether-*b*-ester-*b*-ether).

The block polymer microstructure was examined by ^13^C{^1^H} NMR spectroscopy to understand the regioselectivity of PO ring-opening (73–76 ppm) (Supplementary Fig. 11). The dominant linkages are head-tail (H-T) linkages, with a small preference for isotactic junctions. The selectivity is consistent with other metal-based catalysts for propene oxide ring-opening polymerizations^2^. The relative stereochemistry of the ester-linkages (PE) is a 80:20 mixture of *cis*:*trans* linkages, as determined by reductive degradation experiments using lithium aluminium hydride, and indicating some linkage epimerisation during polymerization (Supplementary Fig. 12).

### Mechanistic hypothesis

**Fig. 2:**
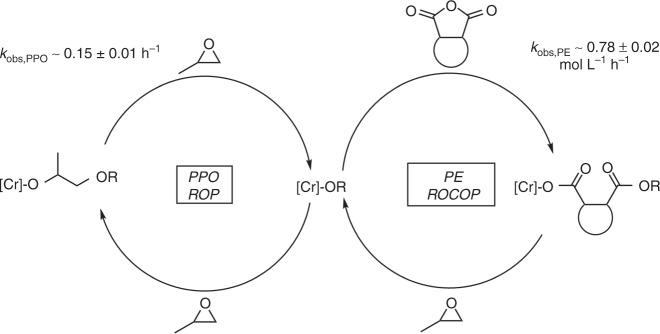
Catalytic cycles. An illustration of the two catalytic cycles and key Cr(III) intermediates during switch catalysis

The triblock polymers are hypothesised to form by switching the catalyst between two different polymerization pathways: anhydride/epoxide ROCOP and epoxide ROP (Fig. 2). The two catalytic cycles connect through a chromium alkoxide intermediate which forms after propene oxide ring-opening. During the first phase of the polymerization, TCA1-PO ROCOP occurs and the kinetic data indicate that anhydride ring-opening is fast (pre-rate limiting) resulting in the catalyst resting state being the chromium carboxylate intermediate. This intermediate subsequently reacts with propene oxide to (re)generate a chromium alkoxide which rapidly inserts the anhydride (Fig. 2, PE ROCOP). The epoxide/anhydride ROCOP progresses until consumption of all the anhydride. At the end-point of the ROCOP excess propene oxide is present and this reacts to form the chromium alkoxide intermediate which catalyses epoxide ROP (Fig. 2, PPO ROP). The high monomer selectivity is proposed to result because of both the Cr-carboxylate thermodynamic stability and the faster rates of anhydride compared to epoxide insertion into the Cr-alkoxide. Indeed, such a combination of thermodynamic and kinetic control was proposed to rationalise high selectivity in epoxide/anhydride/lactone polymerizations using related Cr(III) or Zn(II) catalysts^17,19,23,36^. The precise speciation, nuclearity and coordination environments of these Cr(III) catalytic intermediates are not yet known and, indeed, the metal salen catalyst coordination chemistry remains a hot-topic^42–45^.

### General guidelines for switch catalysis

The catalytic scope and polymer diversity was tested using a range of epoxides, anhydrides and chain transfer agents (Table 1). Switch catalysis was successful using seven epoxides, reacted with TCA1, and produced ABA triblocks (TCA1:epoxide = 200:400, Supplementary Table 3, Supplementary Fig. 13). In each reaction, the monomer conversions were high (typically >95%) for both anhydride and epoxide. The block polymers all showed narrow and monomodal molar mass distributions and differences in molar mass distributions between the block polymers and (individually synthesised) homopolymers substantiates the attachment of all blocks (Supplementary Table 4, Supplementary Fig. 15). All the polyols are amorphous and show a single glass-transition temperature, which can be tuned by the epoxide choice. The *T*_g_ is controllable over a range of ~100 °C, with the highest value resulting from use of styrene oxide (83 °C), and the lowest value (−17 °C) for 2-ethylhexyl glycidyl ether (Supplementary Fig. 14). The results using different epoxides allows understanding of two general guidelines for this catalysis: (i) Cr catalyst systems are successful using a wide range of epoxides but α-substituted epoxides featuring sterically hindered groups show lower rates and (ii) epoxides that could generate resonance stabilised radicals may undergo side-reactions over long reaction times to cross-link (i.e. allyl glycidyl ether)^31^.

**Table 1:** Monomer scope for switch catalysis

Conditions: PPNCl:[Cr]:CTA:A:E = 0.5:1:10:200:400 in d_8_-Toluene (5 M) at 60 °C for 3–6 days; Conversions determined by ^1^H NMR spectroscopy using mesitylene as internal standard; *M*_n_ (*Ð*) values by GPC (THF; 30 °C; based on PS-standards); *T*_g_ values determined by DSC (2^nd^/3^rd^ heating cycle)

^a^Bimodal molar mass distributions observed (only highest intensity peak given)

Three different classes of anhydrides were each investigated in switch catalysis with PO: mono-, bi-, and tricyclic substrates (Table 1, middle, and Supplementary Table 5). Monocyclic derivatives, including maleic anhydride, succinic anhydride, glutaric anhydride or diglycolic anhydride, produce somewhat ill-defined polymers showing molar mass values with high dispersity (*Ð*~1.5–1.9). Unsaturated groups were observed to undergo cross-linking resulting in gelling during isolation of the polymers. It is noted that these anhydrides are known to be challenging to polymerize and only a few ROCOP catalysts have been successfully deployed^44,46^. Bicyclic anhydride derivatives displayed better polymerization control (*Ð*~1.2–1.5) and narrower molar mass distributions correlated with the use of sterically demanding anhydrides. This finding is exactly in line with previous investigations of PO/anhydride ROCOP using Al-salen catalysts^43^. Reactions of CA/PO mixtures formed a statistical copolymer, rather than a block structure^23^. The best control occurred when using tricyclic anhydrides which showed well-defined monomodal molar mass distributions (*Ð* ≤ 1.2). The resulting triblock polymers are all amorphous with glass-transition temperatures from −40 to 34 °C, depending on the structure of the anhydride (Supplementary Fig. 17)^47^. The anhydride scope experiments allow two further guidelines: (iii) Anhydrides showing minimal steric hindrance result in broadened molar mass distributions, perhaps due to transesterification side-reactions; and (iv) Well-defined block polymers result when using sterically encumbered bi- and tricyclic anhydrides^43^.

**Fig. 3:**
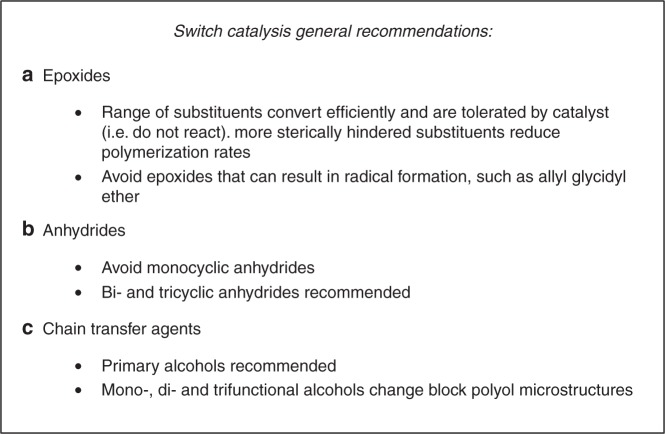
Recommendations for switch catalysis. **a** Epoxide considerations; **b** Anhydride considerations and **c** Chain transfer agent considerations

The scope of structures of alcohols for use as chain transfer agents (CTA) was investigated using TCA1/PO (Table 1, bottom). Primary and secondary alcohols resulted in good control and yielded polymers showing narrow, monomodal molar mass distributions. Tertiary alcohols formed polymers showing bimodal molar mass distributions suggesting that chain-transfer equilibria were not fully established (Supplementary Table 6 and Supplementary Figs. 18 and 19)^48^. Comparing mono-, bi-, tri- and hexafunctional alcohols reveals that as steric bulk increases the chain-transfer reactions slow and more complex product distributions result. The successful synthesis of AB, ABA and star polymers is also possible by using mono-, bi- and trifunctional primary or secondary alcohols. Consequently, two more switch catalysis guidelines are established: (v) Primary alcohol chain transfer agents are recommended for the best control and (vi) Sterically hindered multifunctional alcohols (>3 hydroxyl groups) should be avoided. All the catalysis recommendations are summarised in Fig. 3.

### Multi-switch reactions

**Fig. 4:**
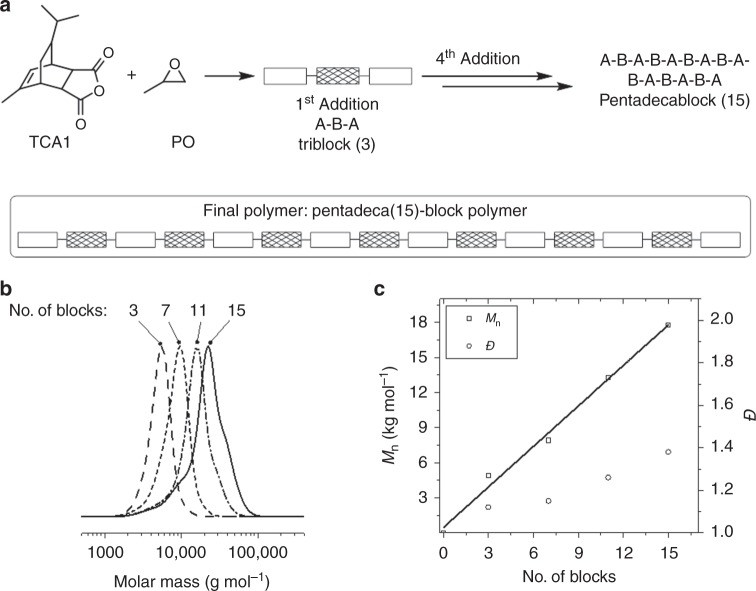
Multi-block polymer syntheses by switch catalysis. **a** Synthesis of pentadeca(15) block polymer; **b** GPC traces showing evolution of molar mass values with block sequence and **c** Plot showing block number against mass, *M*_n_, and dispersity value, *Ð* (GPC). Reaction conditions: [SalcyCrCl]/[PPNCl]/[CHD]/TCA1/PO = 1:0.5:15:200:400, Tol-d_8_ (5 M), 60 °C, 3–5 days. For each subsequent monomer addition, 200 equiv. of TCA1 and 400 equiv. of PO were added

The switch catalyst system is a controlled polymerization and so sequential monomer mixture addition was used to form multi-block polymers (Fig. 4 and Supplementary Table 7). Polymerizations were monitored until >95% propene oxide conversion (by which time anhydride conversion >99%) and this was followed by a further injection of monomer mixture and repeated as necessary. From 1 to 4 mixture additions allowed isolation of multi-block polymers comprising 3, 7, 11 and 15 blocks, respectively. The block sequence selectivity was consistently ABA and was replicated with high control in all the multi-block polymers. Block polymer formation was confirmed after each addition using the following techniques: (i) determining monomer conversion using calibrated ^1^H NMR spectroscopy, (ii) noting the continuous increase in molar mass (with narrow dispersity) as measured by GPC and (iii) DOSY NMR spectroscopy of the final polymer showing a single diffusion coefficient (Supplementary Table 7 and Supplementary Figs. 20–23).

### Polymerization of epoxides, anhydrides and lactones

**Fig. 5:**
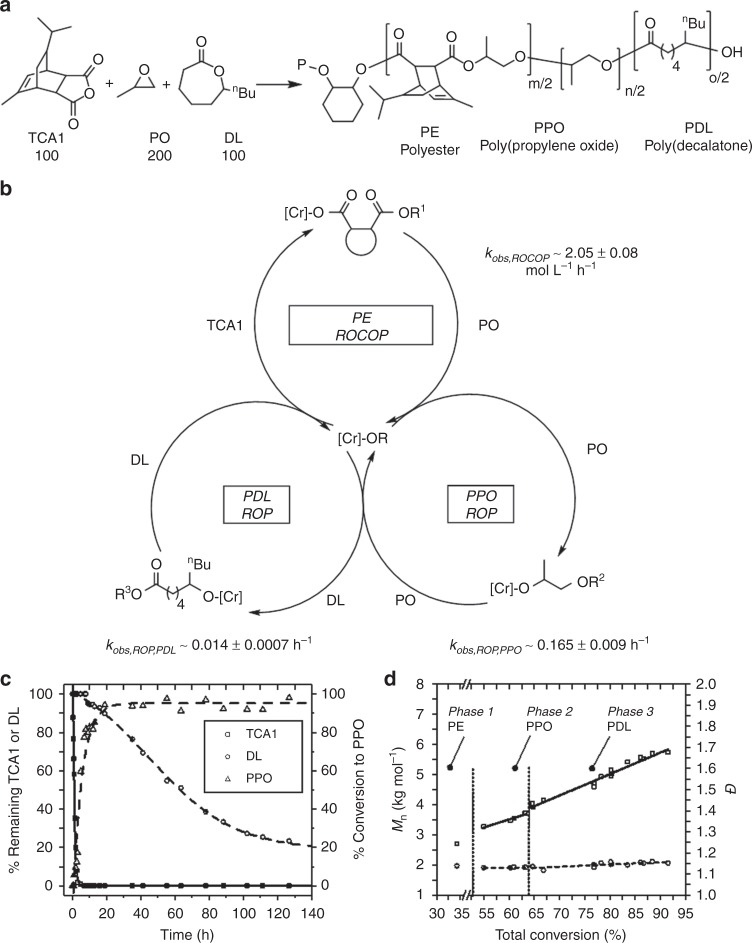
Switch catalysis using mixtures of anhydride (TCA1), epoxide (PO) and lactone (DL). **a** Reaction conditions: [PPNCl]:[Cr]:[CHD]:[TCA1]:[PO]:[DL] = 0.5:1:10:100:200:100, Toluene (5 M, 60 °C); **b** Illustration of three different catalytic cycles connected by the Cr-salen system; **c** Monomer conversion vs. time plot and **d** Plot showing the evolution of *M*_n_ and *Ð* vs. total monomer conversion. The rate of ROCOP and PO ROP are higher than in Fig. 1 due to a higher concentration of catalyst. See Supplementary Fig. 38

We recently discovered that chromium salen catalyst systems also catalyse the ROP of *ε*-decalactone (DL) at 100 °C^23^. It was proposed that another catalytic cycle might be accessible during these switch polymerizations provided there were no significant cross-over between ROP of PO and DL. To test this hypothesis, switch catalysis from mixtures of PO/TCA1/DL was monitored by regular removal of aliquots which were each analysed by ^1^H NMR spectroscopy (against an internal standard; Fig. 5c) and GPC (Fig. 5d). The reaction progresses in three different stages: firstly, the formation of the polyester from TCA1/PO ROCOP occurs, followed by a polyether block from PO ROP. Once the PO conversion is >80%, slow consumption of DL by ROP occurs. The catalysis using three-component mixtures was also highly selective and only a minimal tapered region featuring PPO and PDL was obtained, as indicated by the ^1^H NMR spectra for the final pentablock polymer (Supplementary Figs. 24 and 25). In line with this NMR investigation, reactivity ratio experiments for the two ROP processes, i.e., PO and DL ROP indicate a high kinetic preference for PO ROP and indicate composition drift would only be significant at high conversions (Supplementary Figs. 26 and 27). The catalysis yielded a pentablock polymer (PDL_5_-*b*-PPO_5_-*b*-PE_10_-*b*-PPO_5_-*b*-PDL_5_) and this material showed the desirable polyol low molar mass and narrow dispersity (Supplementary Fig. 31). The chain end-groups were titrated using ^31^P{^1^H} NMR spectroscopy which showed only PDL polyester hydroxyl end-groups (Supplementary Fig. 32). Formation of a single block polymer was confirmed by DOSY NMR spectroscopy (Supplementary Fig. 33). The material shows an amorphous structure and its glass transition is −9 °C which is significantly lower than the triblock material prepared only from TCA1/PO (30–40 °C; Supplementary Fig. 34). This further variant on switch catalysis connects three different catalytic cycles: epoxide/anhydride ROCOP, epoxide ROP and lactone ROP. It shows very high selectivity and kinetic analysis reveals higher relative rates for PO compared to DL ROP, which is consistent with the experimentally observed product selectivity (block order) (Fig. 5b).

### Polymer properties and scope

This switchable catalysis offers a straightforward and rather general route to hydroxyl-telechelic block polymers (polyols). The focus of this work is necessarily to establish the guidelines, scope and proof for the catalysis. To demonstrate the potential for the polyols, a representative sample (PPO-*b*-PE-*b*-PPO) was reacted with diphenylmethane-4,4′-diisocyanate (MDI) to prepare the corresponding polyurethane. First, the number of hydroxyl groups on the polyol was determined using a ^31^P{^1^H} NMR titration (accounting for differing relaxation rates). The titration involves reaction of the dry polyol with 2-chloro-4,4,5,5-tetramethyl-1,3,2-dioxaphospholane and benchmarking the resulting integrals by comparison against an internal standard (Supplementary Figs. 35–37)^49^. With the hydroxyl-number in hand, the correct ratio of polyol:isocyanate groups was adjusted so as to access a higher molar mass PU material. After the reaction the polymer molar mass increased from 3700 g mol^−1^ (polyol) to 45,000 g mol^−1^ (polyurethane) and the expected consumption of hydroxyl end groups was further confirmed by ^1^H NMR spectroscopy (Supplementary Fig. 39). This experiment demonstrates the facility to use these polyols to prepare a range of higher polymers, including useful polyurethanes.

Over the series of polyols produced using switchable catalysis, it is straightforward to control the molar mass values within the range 3000–20,000 g mol^−1^, although with increased dispersity for higher molar mass. All polyols are amorphous materials with glass transition temperatures over the range −68 °C < *T*_g_ < 73 °C, depending on the block compositions (Supplementary Tables 9–12). Some of the resulting polyols feature alkene groups in the repeat unit and the post-functionalization of a representative sample was evaluated. The thiol-ene reaction is a well-known method to cross-link polyol resins; it is used industrially to produce coatings^50,51^ or elastomers^52^ and popular in research science to install functional groups for biomedical applications^53^. The triblock polyol prepared from VPO/TCA1 was successfully reacted with two representative thiols: butane thiol as a model hydrophobic substituent and 2-mercaptoethanol as a model hydrophilic group (Supplementary Figs. 47 and 48)^53,54^.

In conclusion, a versatile and straightforward one-pot synthesis of oxygenated block polymers featuring polyester and polyether segments was demonstrated. The synthesis takes advantage of the ability to switch the Cr-catalyst between epoxide/anhydride ring-opening copolymerization and epoxide or lactone ring-opening polymerization. In the simplest case, a series of triblock poly(ether-*b*-ester-*b*-ether) were produced from a wide range of aromatic, aliphatic, functionalized and substituted monomers. By taking advantage of the high degree of polymerization control, three component mixtures yielded ABCBA type poly(ester-*b*-ether-*b*-ester’-*b*-ether-*b*-ester) and sequential additions of the three-component mixture formed predictable multi-block polymers of the type (ABA)_n_ (*n* = 3, 7, 11, 15). A notable advantage of this catalytic route is the ability to produce multi-component and functional polymer structures very easily. It also provides access to classes of materials which are compatible with existing coupling and cross-linking chemistries relevant to production of polyurethanes or polyester resins. Access to more complex but controlled block polyol structures is expected to furnish materials with properties matched to high-value emerging applications such as energy storage and medicine.

## Methods

### General switch polymerisation protocol

In a glovebox, the catalyst, co-catalyst, chain-transfer agent, monomers, internal standard (mesitylene) and solvent were added to a flame-dried vial which was equipped with a magnetic stir bar. The reaction mixture was sealed tightly with a melamine-cap containing a Teflon inlay and with electrical isolation tape, which was held in place by a rubber band. The polymerisations were monitored by regular removal of aliquots which were exposed to air to quench the polymerisation. The reaction (monomer) conversion was determined by ^1^H NMR spectroscopy by integrating diagnostic monomer signals and use of an internal standard. The polymer molar mass and dispersity values were determined by GPC, at 30 °C using THF as the eluent and narrow molar mass polystyrene calibrants. The end group assay with 2-chloro-4,4,5,5-tetramethyl dioxaphospholane was previously reported by Spyros and co-workers^55^. Where necessary, polyols were purified by precipitation from pentane. A representative procedure involved adding [SalcyCrCl] (1 equiv., 9.5 mg, 15 μmol), PPNCl (0.5 equiv., 21 μL of a stock solution of 50 mg PPNCl in 250 μL dry acetonitrile [equivalent to 4.3 mg, 7.5 μmol]), CHD (10 equiv., 17.4 mg, 150 μmol), anhydride (200 equiv., 3.00 mmol) and epoxide (400 equiv., 6.00 mmol) to toluene-*d*_*8*_ (550 μL to obtain a 5 M solution in anhydride). Detailed experimental procedures and spectroscopy results are provided in the supporting information.

## Supplementary information

Supplementary Information

## Data Availability

The data that support the findings of this study are available from the corresponding author upon reasonable request.
