# Supplementary material for: Easy access to oxygenated block polymers via switchable catalysis
Source: Nat Commun. 2019 Jun 17;10:2668. doi: 10.1038/s41467-019-10481-w (PMC6572807; doi:10.1038/s41467-019-10481-w)
Supplement: Supplementary file 1 — Supplementary Information [file 41467_2019_10481_MOESM1_ESM.pdf]

Supporting Information

Easy Access to Oxygenated Block Polymers via Switchable Catalysis

Stößer et al.

Supplementary Methods

Materials

[SalcyCrCl] was purchased from Strem Chemicals and stored inside a glovebox; PPNCl (97%, Sigma Aldrich) was recrystallised from anhydrous acetonitrile/diethyl ether, dried at 40 °C under high vacuum overnight and stored inside a glovebox. (±)-Propylene oxide (99%, Sigma Aldrich) was purified by fractional distillation after drying over calcium hydride overnight and TCA1 was prepared according to a literature procedure. All anhydrides were recrystallised and sublimed under high vacuum (10^{-2} mbar, 60-100 °C) before use; phthalic anhydride was further stirred in anhydrous benzene overnight, filtered, evaporated, and recrystallised from anhydrous chloroform before sublimation. Chain-transfer agents were recrystallised and sublimed before use (if solid) or dried over calcium hydride and fractionally distilled (if liquid).

Characterisation Techniques

DSC thermograms were recorded on a Mettler Toledo DSC 3 Star calorimeter. First, the sample was kept at 25 °C for 1 minute, then heated to 180 °C at 10 °C min^{-1}, kept for 1 minute and cooled down to -90 °C and kept for 1 minute; the nitrogen flow rate was 80 mL min^{-1}. This heating / cooling cycle was then repeated three times and the data from the second or third heating cycle is reported. NMR spectra were measured on a Bruker Avance III HD nanobay NMR equipped with a 9.4T magnet (^{1}H 400.2 MHz, ^{13}C 100.6 MHz), a Bruker Avance NMR equipped with a 11.75T magnet and a ^{13}C detect cryoprobe (^{1}H 500.3MHz, ^{13}C 125.8MHz) or a Bruker AV 400 (^{1}H 400 MHz, ^{13}C 100 MHz) instrument. All spectra were recorded in $CDCl_3$.[1] Molar masses were determined by SEC on an Agilent PL GPC-50 instrument, with HPLC grade THF (flow rate: 1.0 mL min^{-1}) after calibration with near monodisperse polystyrene standards. Samples were dissolved in THF (ca. 10 mg mL^{-1}) and filtered through a 2 μm filter before injection.

General Polymerisation Procedure

In a nitrogen-filled glovebox, CHD, PPNCl, [SalcyCrCl], TCA1, PO and toluene were added to a flame dried vial equipped with a rare-earth magnetic stir bar and sealed with a melamine cap containing a Teflon inlay and electrical isolation tape (PVC). The vial was then placed in a pre-heated aluminium block (60 °C) and reacted for the specified time. Typical amounts for [TCA1]:[PO]:[CHD]:[SalcyCrCl]:[PPNCl]=200:400:10:1:0.5 were 700 mg (TCA1, 3 mmol), 420 µL (PO, 6 mmol), 17.4 mg (CHD, 225 µmol), 9.5 mg ([SalcyCrCl], 15 µmol), 4.3 mg ([PPNCl], 7.5 µmol – based on a stock solution of 50 mg PPNCl in 250 µL of anhydrous acetonitrile) and 0.6 mL of toluene. To determine conversions, 5 equiv. of mesitylene were added as an internal standard; the polymers were typically isolated by precipitation from pentane, drying under high vacuum, or filtration over silica followed by drying under high vacuum. Please note that the purification methods resulted in isolated yield values which were, in some cases, significantly lower than the overall conversions.

Large Scale Procedure

TCA1 (25.0 g, 211 mmol, 200 equiv.), PO (15 mL, 422 mmol, 400 equiv.), CHD (1.24 g, 10.6 mmol, 10 equiv.), [SalcyCrCl] (675 mg, 1.06 mmol, 1.00 equiv.), PPNCl (306 mg, 527.5 mmol, 0.5 equiv.) and toluene (21.25 mL) were added a 100 mL stainless steel Paar reactor inside a nitrogen-filled glovebox and sealed. The reaction was stirred for 4 days at 60 °C before exposure to air. The conversions and molar mass was determined from this crude reaction mixture and the polymer was purified by filtration over silica (three times) and concentration to dryness.

Post-Functionalisation Reaction

Synthesis of Pre-Polymer:

The general procedure was followed TCA1 (5.00 g, 21.34 mmol, 200 equiv.), 3,4-epoxy-1-butene (3.44 mL, 42.68 mol, 400 equiv.), cyclohexane diol (124 mg, 1.07 mmol, 10 equiv.), [SalcyCrCl] (67.65 mg, 107 µmol, 1 equiv.), PPNCl (30.4 mg, 53 µmol, 0.5 equiv.) and 4.2

mL of toluene. After four days, the mixture was dried under high vacuum, dissolved in dichloromethane and filtered over a silica plug to remove the catalyst. The obtained solution was concentrated and dried overnight under high vacuum (yield: 600 mg).

Thiol-ene Reactions

Inside a glovebox, the polymer (100 mg, 1.0 equiv.) was dissolved in 0.5 mL of anhydrous (deuterated) chloroform and dimethylacetophenone (10 mg, 0.1 equiv.) and the corresponding thiol (30 equiv.) were added. The vial was sealed in the glovebox, exposed to UV light (Philips Facial Tanner HB172, 4x15 W) in a distance of ca. 10 cm and stirred for 2 hours before analysis by ^{1}H NMR spectroscopy and GPC.

MDI Chain-Extension Reaction

The polymer (M_n = 3,700 g mol^{-1}; 0.5 g), MDI (360 mg), tin(II) octoate (45 μL) and toluene (5.5 mL) were added to a flame-dried vial and the mixture was reacted overnight at 60 °C before being quenched by exposure to air. The viscous solution was dried under high vacuum, dissolved in a minimal amount of DCM and precipitated from methanol (three times).

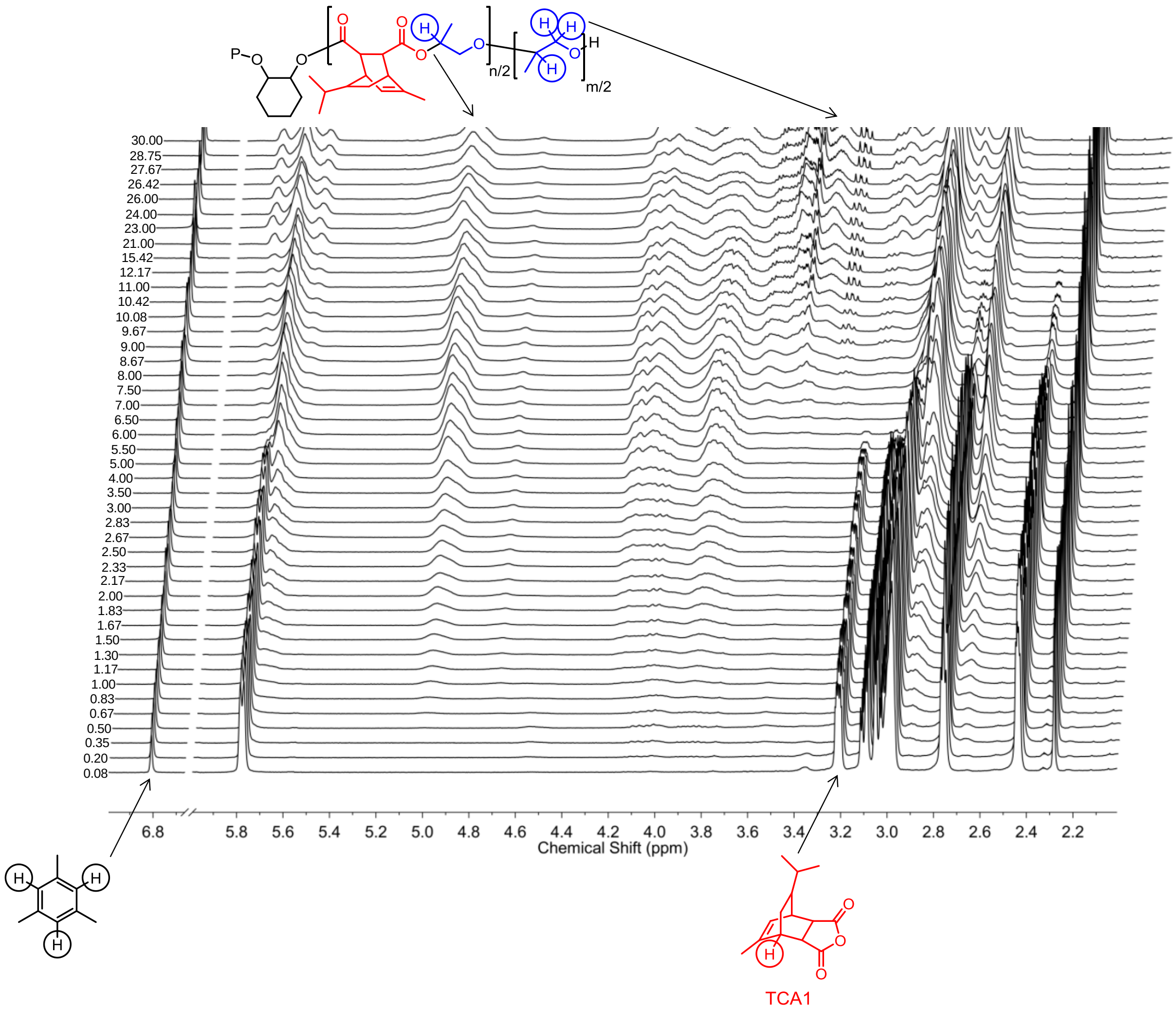

Supplementary Figure 1 – ^{1}H NMR spectra at different times of the one-pot polymerization of TCA1/PO.

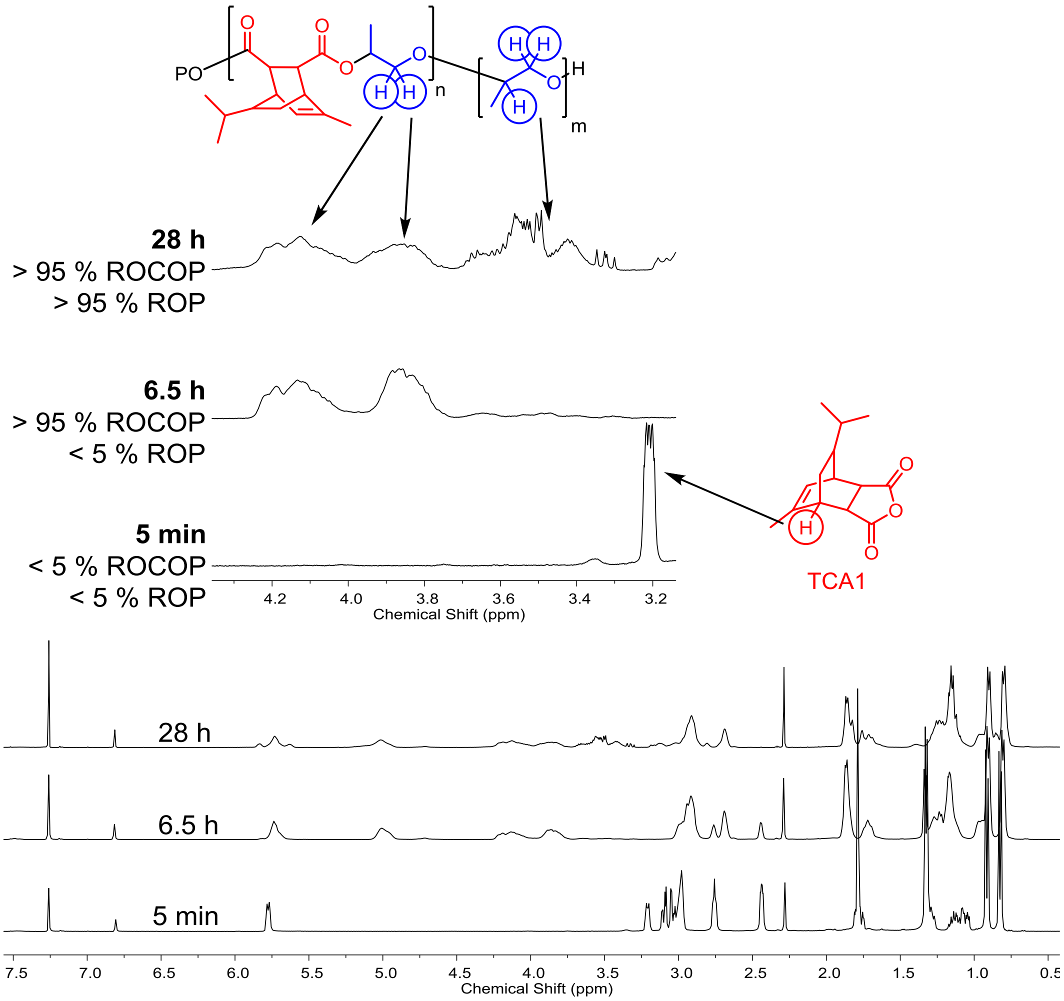

Supplementary Figure 2 – Selected ^{1}H NMR spectra of one-pot polymerization of TCA1/PO at different conversions.

Supplementary Table 1 – Integrals based on ^{1}H NMR spectroscopy used for conversion vs. time plots.

Time (h)	Anhydride (3.27-3.17)	Polyester (5.15-4.83)	Polyether (3.72-3.29)	Normalized Anhydride	Normalized Polyester[a]	Normalized Polyether
0.08	6.69	0.00	0.00	100.00	0.00	0.81
0.20	6.13	0.00	0.15	91.63	0.00	0.75
0.35	5.95	0.00	0.14	88.94	0.00	0
0.50	5.71	0.19	0.00	85.35	3.12	0.48
0.67	5.53	0.16	0.09	82.66	2.63	1.08
0.83	5.49	0.37	0.20	82.06	6.08	0
1.00	5.23	0.45	0.00	78.18	7.39	0.70
1.17	5.11	0.62	0.13	76.38	10.18	0.75
1.33	5.00	0.85	0.14	74.74	13.96	1.08
1.50	4.88	1.06	0.20	72.94	17.41	1.40
1.67	5.07	1.20	0.26	75.78	19.70	0
1.83	4.35	1.42	0.00	65.02	23.32	0.05
2.00	4.18	1.52	0.01	62.48	24.96	1.35

2.17	4.35	1.82	0.25	65.02	29.89	0.97
2.33	3.80	1.90	0.18	56.80	31.20	0.05
2.50	3.63	1.99	0.01	54.26	32.68	0.75
2.67	2.94	2.20	0.14	43.95	36.12	0
2.83	3.39	2.38	0.00	50.67	39.08	1.02
3.00	3.47	2.55	0.19	51.87	41.87	0
3.50	2.67	3.28	0.00	39.91	53.86	0
4.00	1.99	3.46	0.00	29.75	56.81	0.59
5.00	0.88	4.75	0.11	13.15	78.00	1.62
5.50	0.47	5.20	0.30	7.03	85.39	2.59
6.00	0.17	5.63	0.48	2.54	92.45	5.88
6.50	0.00	5.86	1.09	0.00	96.22	12.47
7.00	0.00	5.84	2.31	0.00	95.89	14.20
7.50	0.00	5.84	2.63	0.00	95.89	22.20
8.00	0.00	5.86	4.11	0.00	96.22	32.25
8.67	0.00	5.85	5.97	0.00	96.06	38.51
9.00	0.00	5.74	7.13	0.00	94.25	42.40
9.67	0.00	5.97	7.85	0.00	98.03	51.05
10.08	0.00	5.65	9.45	0.00	92.78	51.10
10.42	0.00	6.00	9.46	0.00	98.52	52.35
11.00	0.00	6.13	9.69	0.00	--*	53.48
12.17	0.00	6.09	9.90	0.00	100.00	60.94
15.42	0.00	5.93	11.28[+]	0.00	97.37	64.77
21.00	0.00	5.56	11.99	0.00	91.30	67.42
23.00	0.00	5.58	12.48	0.00	91.63	62.18
24.00	0.00	6.05	11.51	0.00	99.34	64.82
26.00	0.00	6.06	12.00	0.00	99.51	--*
26.42	0.00	6.55	13.41	0.00	--*	61.69
27.67	0.00	5.93	11.42	0.00	97.37	64.12
28.75	0.00	5.89	11.87	0.00	96.72	--*
30.00	0.00	5.89	12.55	0.00	96.72	--*

[a] Calculated from integral at 5.88-5.55 pm (corresponds to C=C of polyester); * not considered.

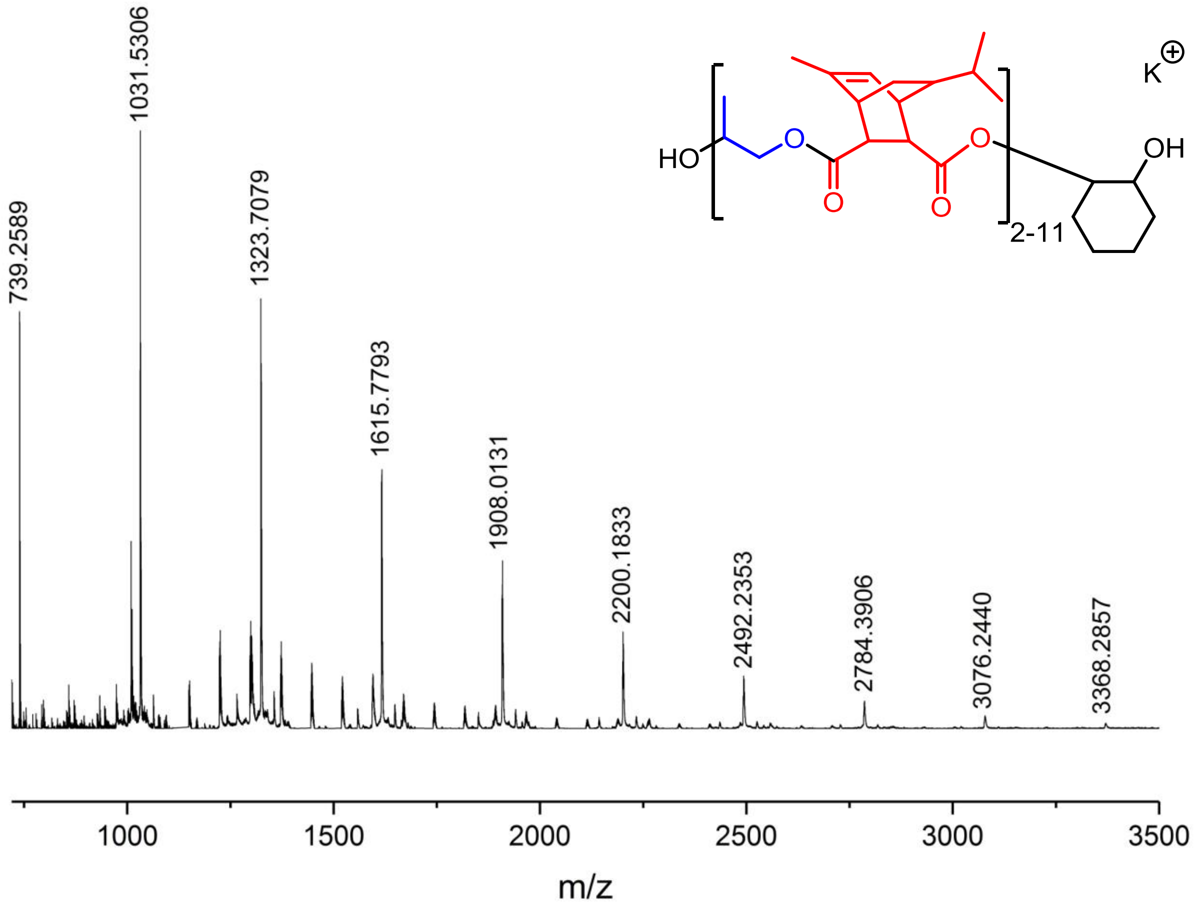

Supplementary Figure 3 – MALDI Spectrum of crude sample of one-pot polymerization of TCA1/PO after 2h (25% polyester formation, < 5% polyether formation, M_n=1,790 g mol^{-1} ($Đ$=1.13). The peaks correspond to a perfectly alternating polyester (n * 292.167 with n=2-11) + CHD (116.08) + K^+ (38.96). A second distribution with a repeating unit of m/z=74.00 was assigned to polysiloxane, a common trace impurity in high-resolution mass spectrometry (not shown).[2,3]

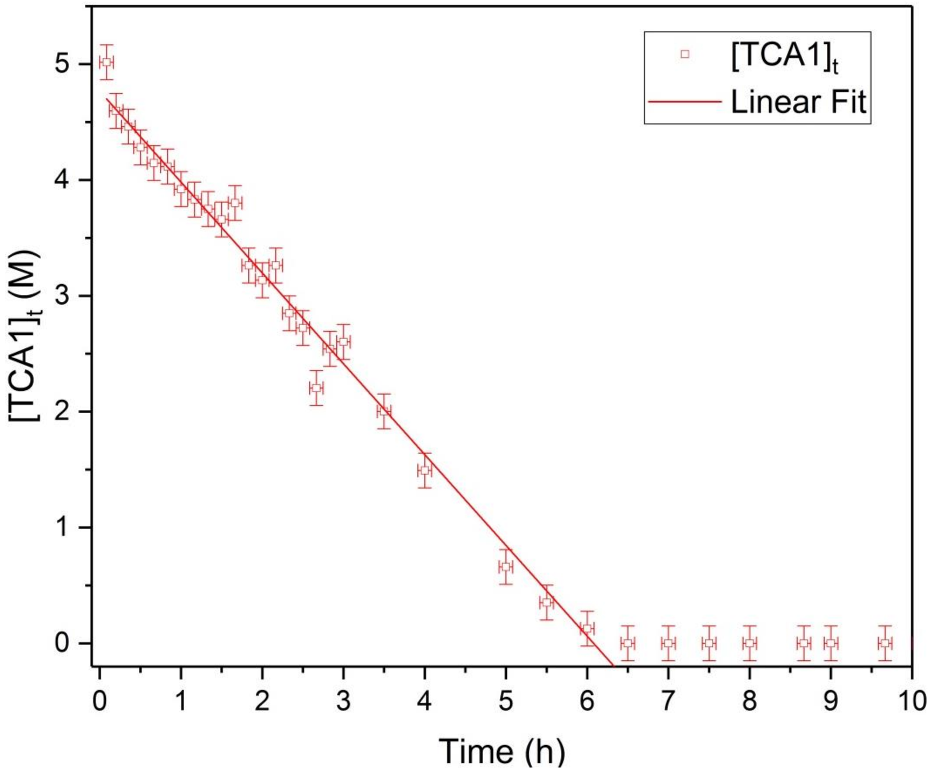

Equation	y = a + b*x
Plot	W
Weight	Instrumental
Intercept	4.76 ± 0.06
Slope	-0.78 ± 0.02
Residual Sum of Squares	32.70
Pearson's r	-0.99
R-Square(COD)	0.99
Adj. R-Square	0.98

Supplementary Figure 4 – Zeroth-order plot for TCA1/PO ROCOP. Based on integrals from 1H NMR spectroscopy after normalizing against mesitylene as an internal standard. The following errors were applied: y-axis ± 3 % and x-axis ± 0.08 h.

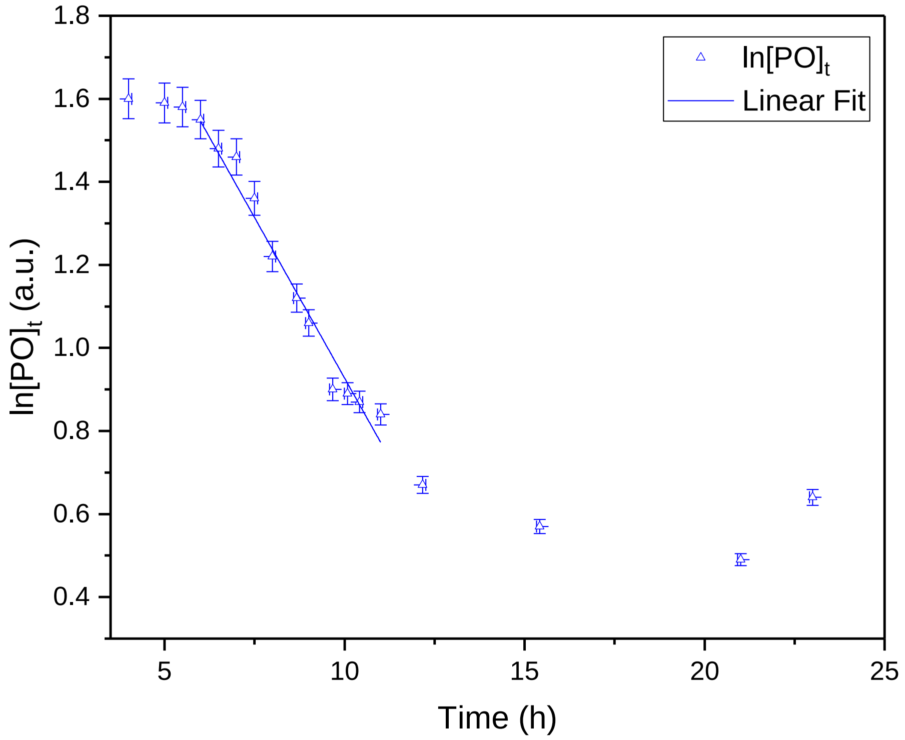

Equation	y = a + b*x
Plot	W
Weight	No Weighting
Intercept	2.47343 ± 0.09468
Slope	-0.1546 ± 0.01017
Residual Sum of Squares	21.16241
Pearson's r	-0.98108
R-Square(COD)	0.96251
Adj. R-Square	0.95835

Supplementary Figure 5 – Semi-logarithmic first-order plot for the polymerisation of PO. Based on conversion vs. time data based on integrals of PPO from ^{1}H NMR spectroscopy after normalizing against mesitylene as an internal standard. The following errors were applied: y-axis ± 3 % and x-axis ± 0.08 h.

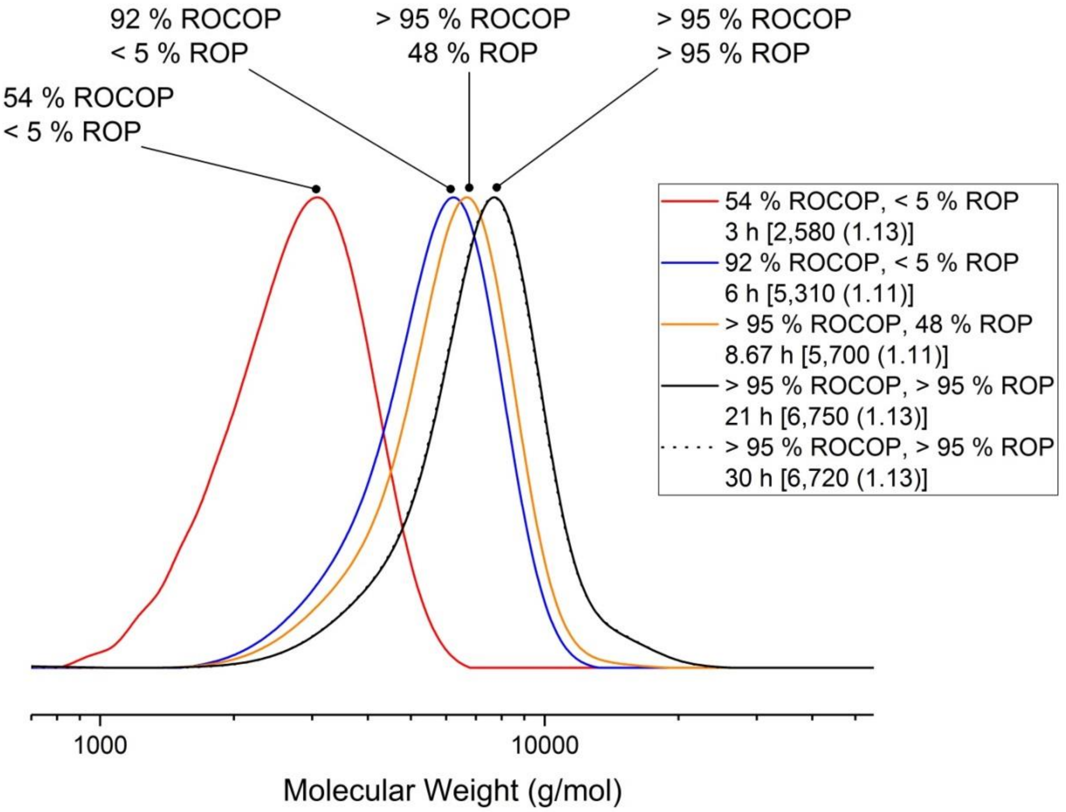

Supplementary Figure 6 – Selected GPC traces during the one-pot polymerization of TCA1/PO at different stages of the reaction. Reaction times are given in the legend; M_n values (in g mol^{-1}) and $Đ$ values are given in brackets.

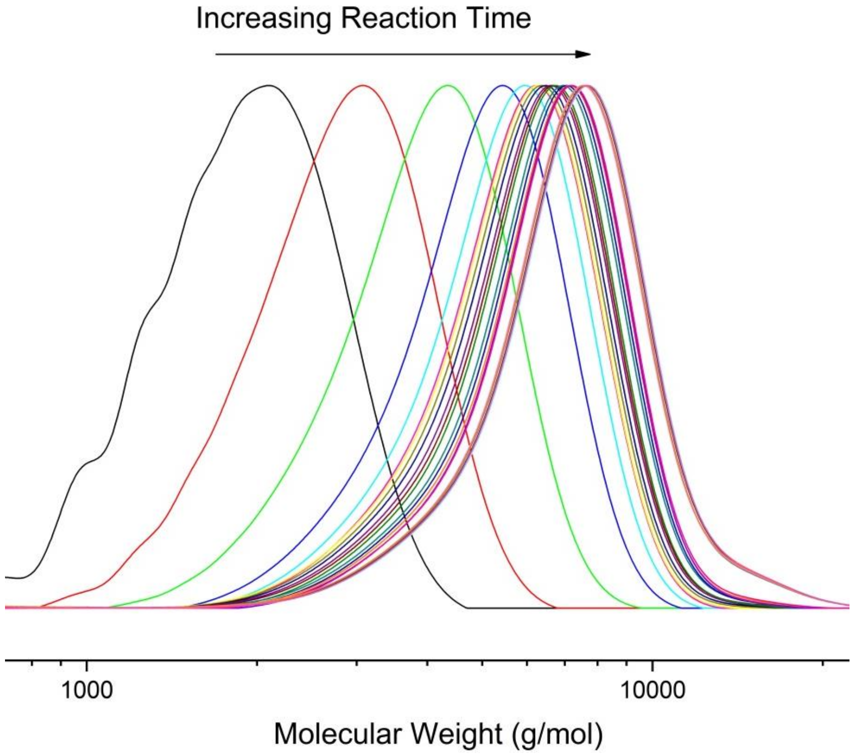

Supplementary Figure 7 – GPC traces during the one-pot polymerization of TCA1/PO. Time, M_n and $Đ$ values are given in Supplementary Table 2.

Supplementary Table 2 – M_n (g mol^{-1}) and *Đ* values of GPC traces during the one-pot polymerization of TCA1/PO.

Time (h)	M_n (g mol^{-1})	*Đ*
1	950	1.10
2	1,790	1.13
3	2,580	1.13
4	3,670	1.12
5	4,630	1.11
5.5	5,040	1.12
6	5,310	1.11
6.5	5,320	1.11
7	5,430	1.11
7.5	5,540	1.11
8	5,630	1.11
8.67	5,700	1.11
9	5,790	1.11
9.67	5,960	1.11
10	6,070	1.11
10.42	6,150	1.11
12.17	6,220	1.10
15.42	6,184	1.11
21	6,750	1.13
23	6,650	1.12
24	6,770	1.12
26	6,780	1.12
26.25	6,610	1.13
27.67	6,700	1.13
28.75	6,680	1.12
30	6,720	1.13

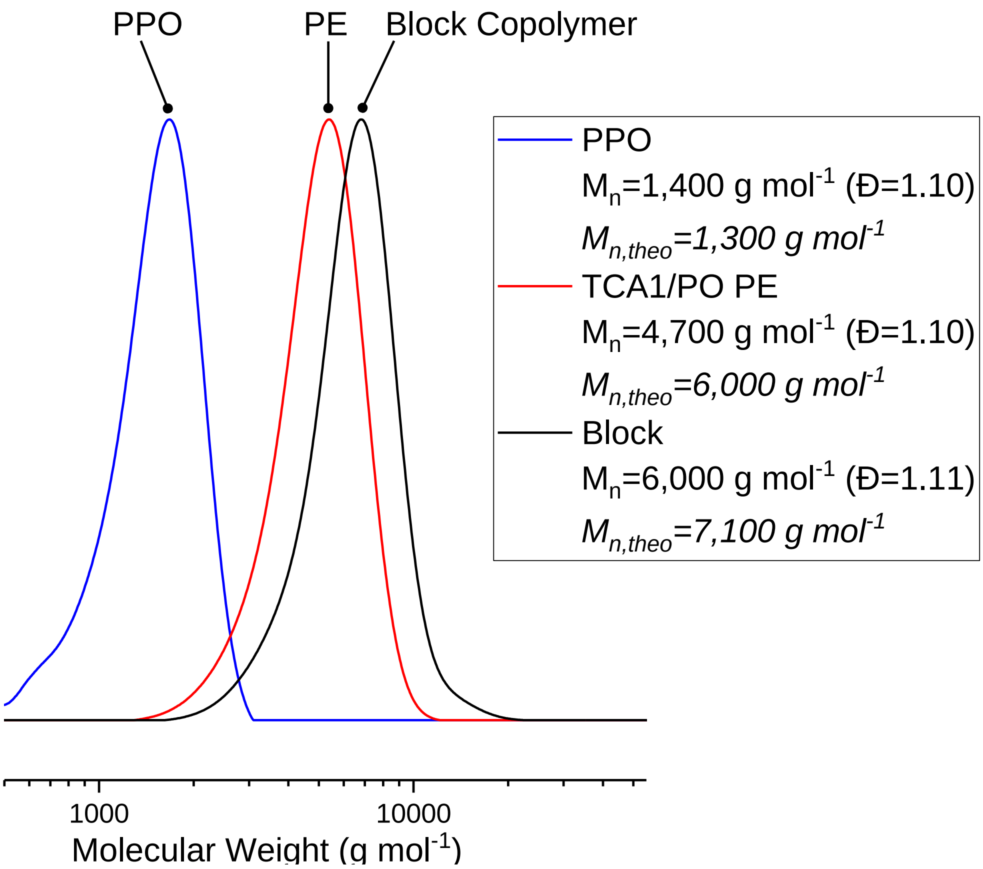

Supplementary Figure 8 – Comparison of GPC traces of PPO, PE and the corresponding block copolymer (DP = 20 in all cases).

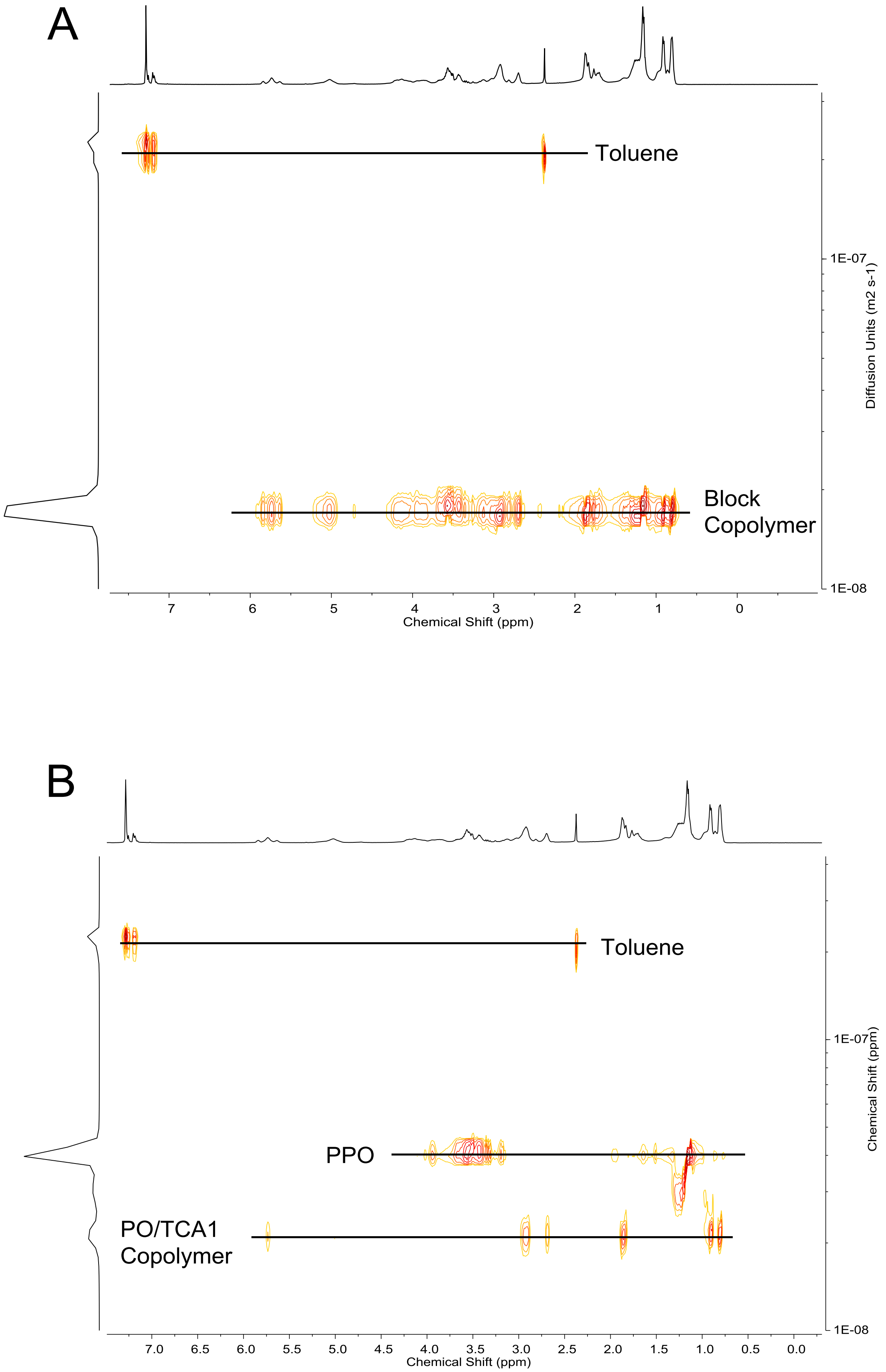

Supplementary Figure 9 – DOSY NMR Spectra of block copolymer (top) and blend of homopolymers (bottom). In both cases, samples with similar theoretical molecular weight were used.

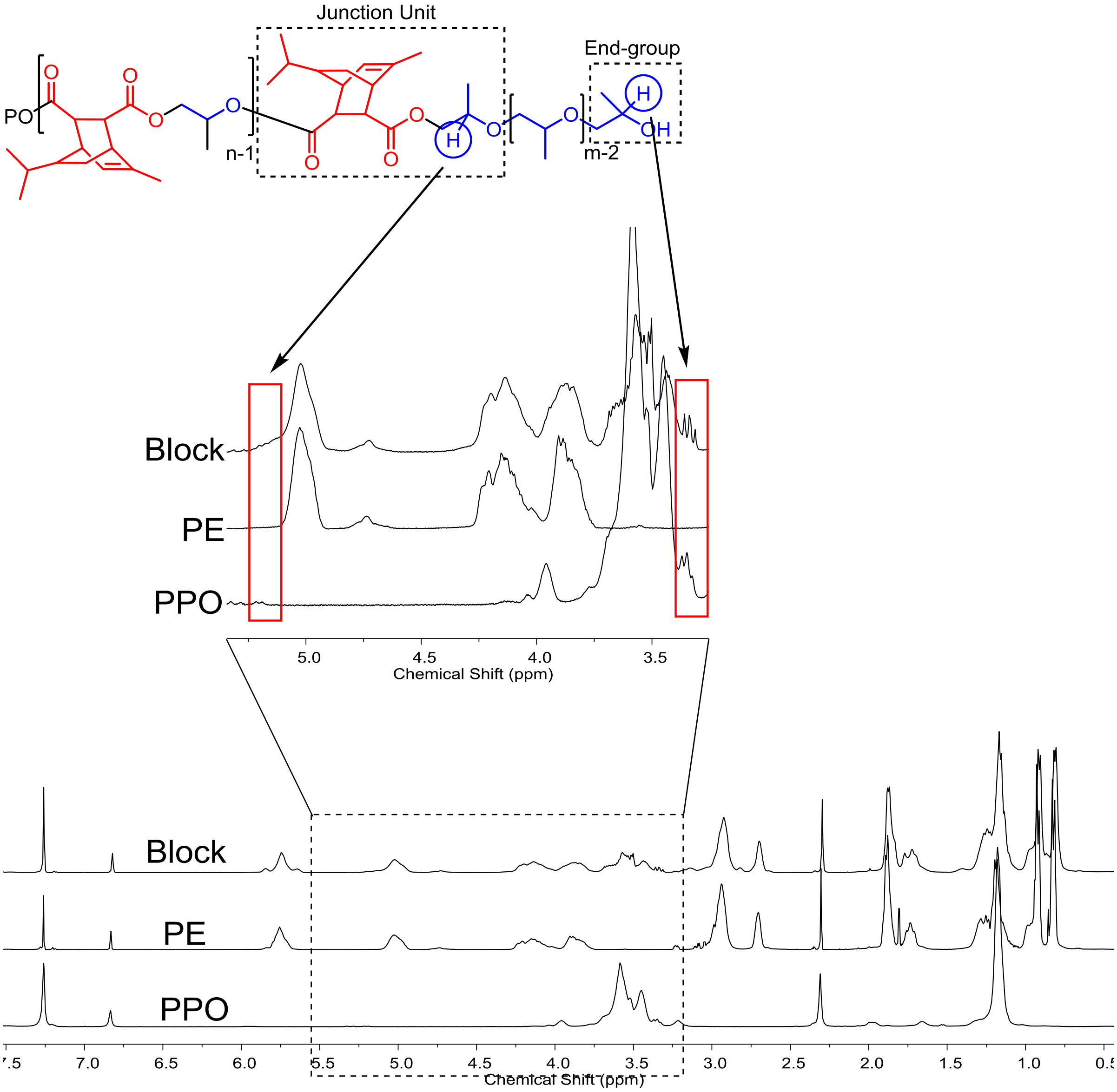

Supplementary Figure 10 – ^{1}H NMR spectra of homopolymers (PPO, PE) and the block copolymer.

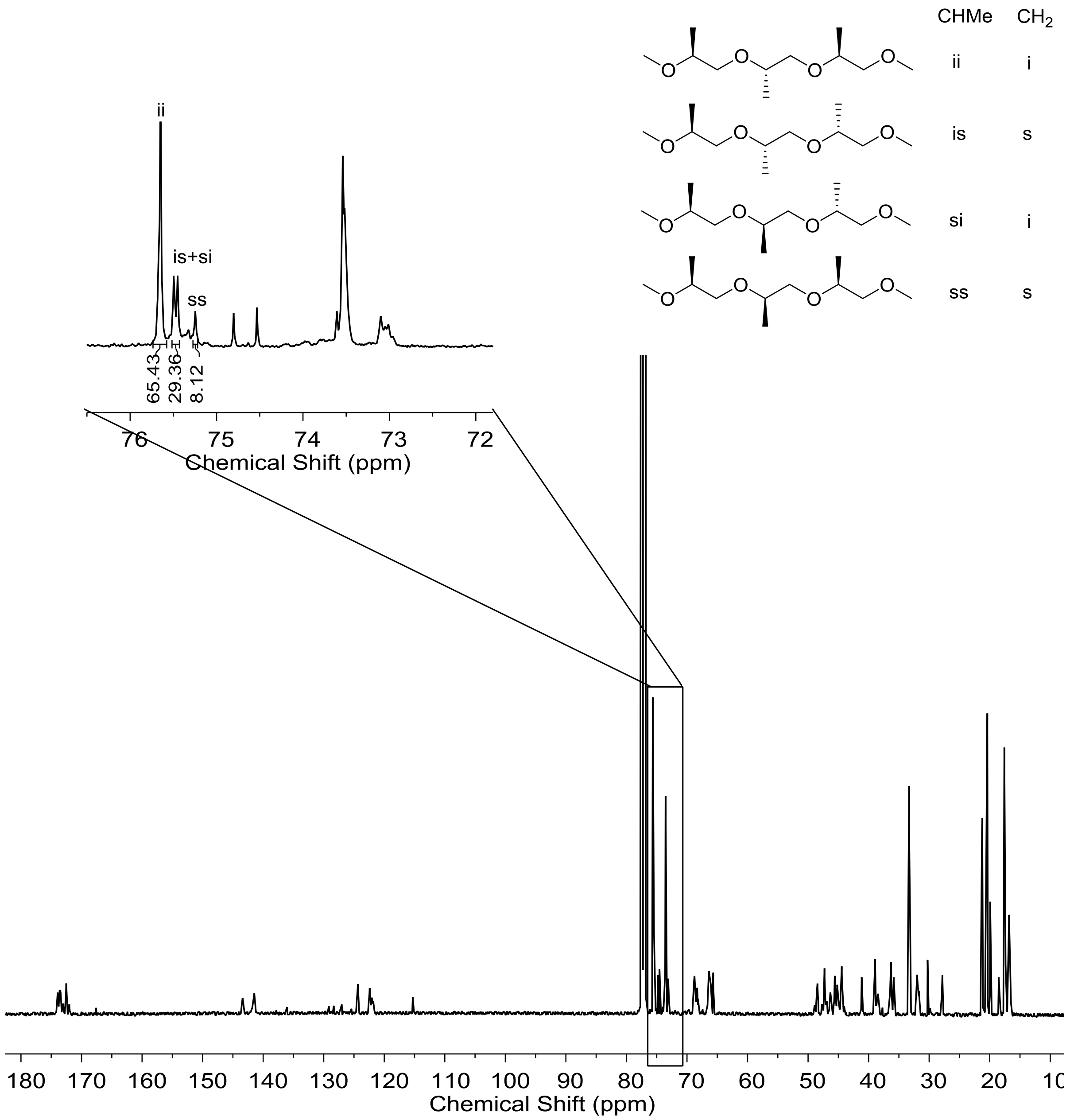

Supplementary Figure 11 – $^{13}C\{^1H\}$ NMR Spectrum of polymer obtained from TCA1/PO (top) and extended region used for the analysis of PPO (bottom).[4]

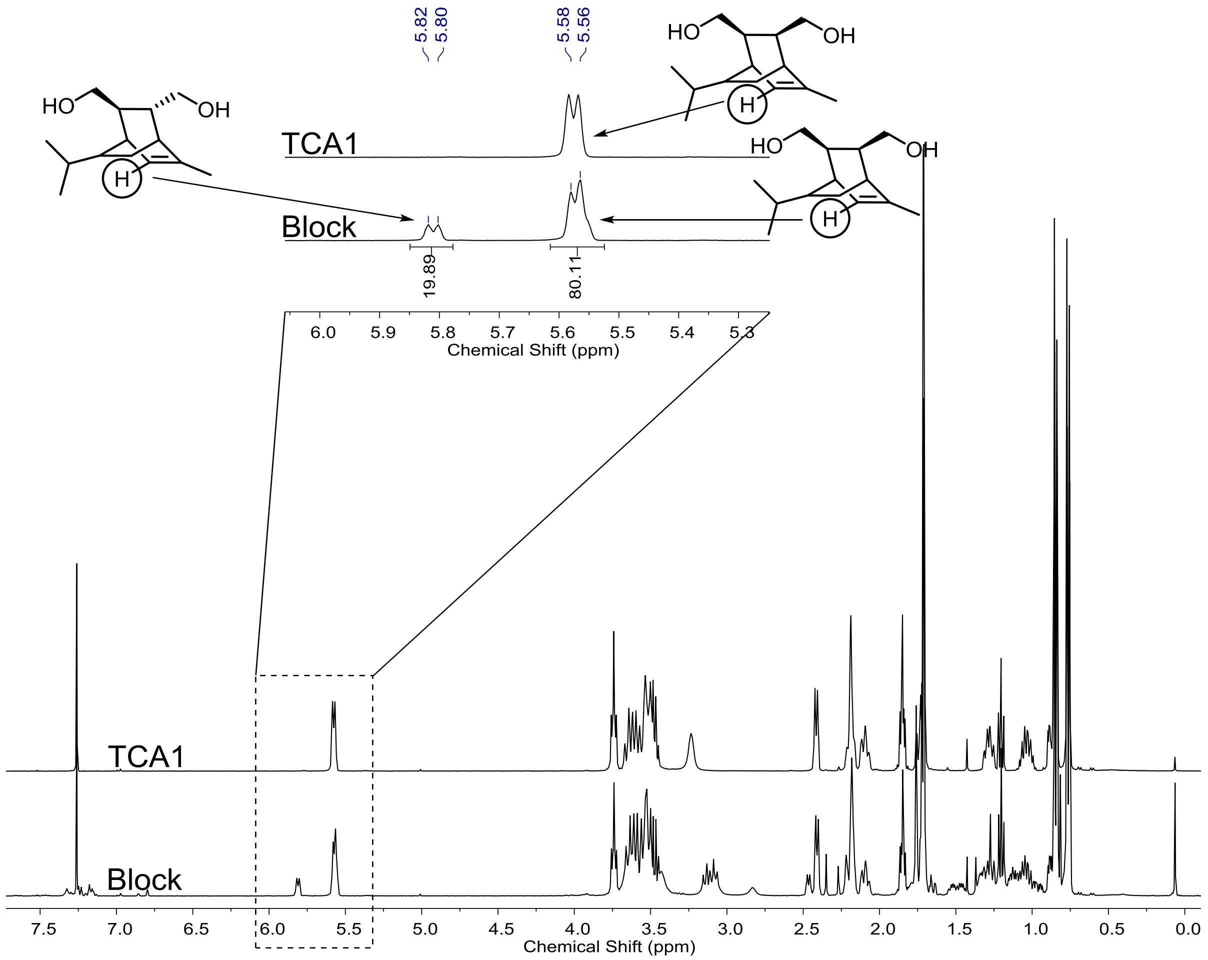

Supplementary Figure 12 – ^{1}H NMR Spectra of degradation products from TCA1 (top left) and the polymer obtained from TCA1/PO (bottom left).

Supplementary Table 3 – Epoxide Scope.

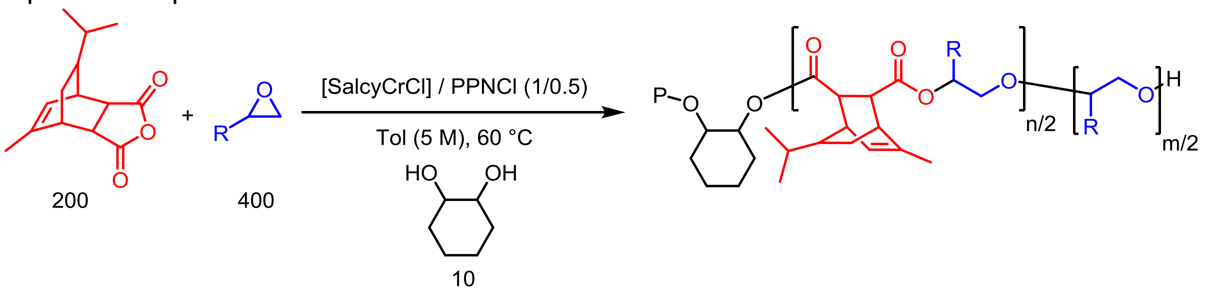

Epoxide	Shift Epoxide | Shift TCA1 (¹H NMR)	Conversion Epoxide | TCA1	M_n (*Đ*)	T_g (°C)
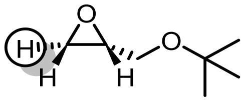 tert-Butyl glycidyl ether	2.64-2.60 (m) | 1.04-1.00 (m)	3 d: 50 % | 99 %<br>6 d: > 95 % | 99 %	3d: 6,700 (1.10)<br>6d: 7,900 (1.11)	60 °C
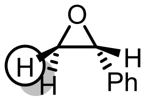 Styrene oxide	3.16-3.03 (m) | 3.07-3.00 (m)	3 d: 73 % | 99 %<br>6 d: 83 % | 99 %	3d: 5,700 (1.14)<br>6d: 5,900 (1.15)	83 °C
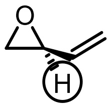 2-Vinyloxirane	3.37-3.27 (m) | 3.21-3.16 (m)	3 d: > 95 % | 99 %	6,400 (1.14)	44 °C
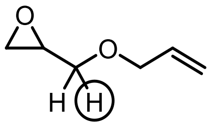 Allyl glycidyl ether	3.45-3.35 (m) | 1.04-1.00 (m)	3 d: > 95 % | 99 %	8,100 (1.12)	N. D.*
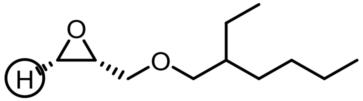 2-Ethylhexyl glycidyl ether	2.64-2.59 (dd) or 2.88-2.75 (dd) | 1.04-1.00 (m)	5 d: > 95 % | 99 %	5 d: 7,100 (1.10)	-17 °C
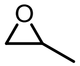	3.74-3.25 (dd) | 1.04-1.00 (m)	1 d: 83 % | 99 %	6,700 (1.13)	41 °C

Propylene oxide				

# Soluble in pentane (not isolated).
* Insoluble material obtained after isolation.

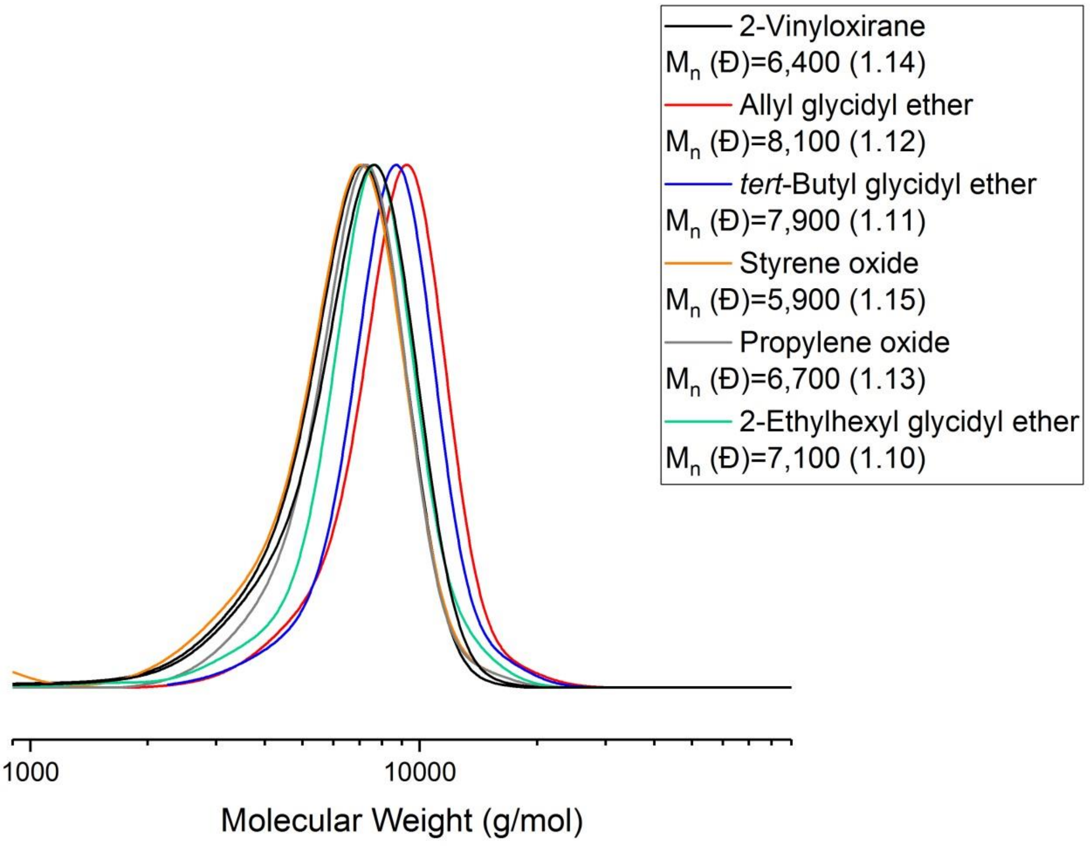

Supplementary Figure 13 –GPC traces of substrates shown in Supplementary Table 3.

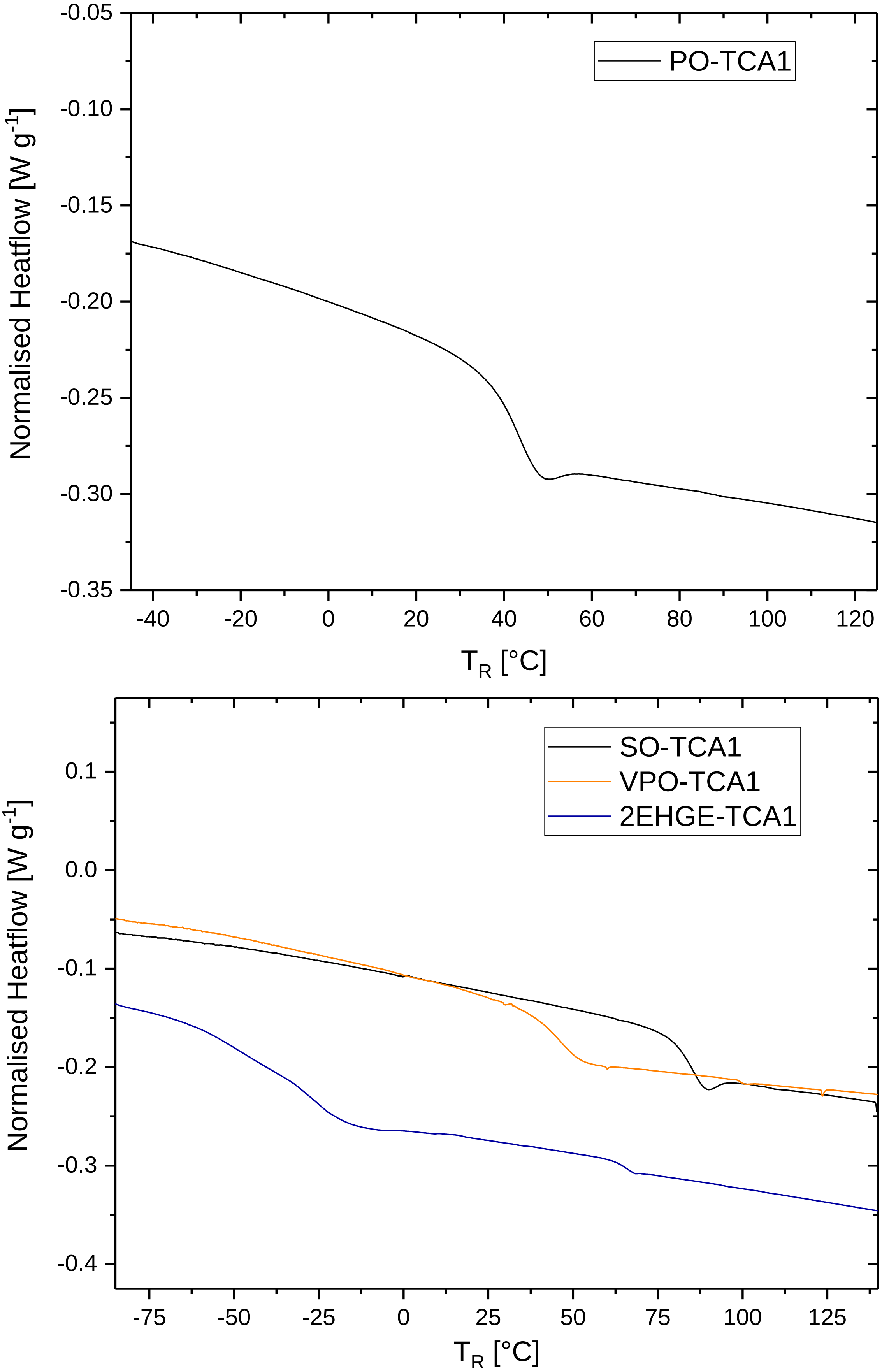

Supplementary Figure 14 – DSC thermograms for selected epoxides substrates shown in Supplementary Table 3.

Supplementary Table 4 – Synthesis of Homo- and Block Copolymers for Selected Epoxides.

**Epoxide**	**Starting Material**	**Shift Epoxide<br>Shift TCA1 (^{1}H NMR)**	**Conversion (Epoxide | Anhydride)**	M_n (**Đ**)
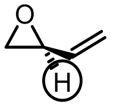<br>2-Vinyloxirane	vPO (200 equiv.)	3.37-3.27 (m)<br>2.83-2.56 (m)	99 % (5d)	1,350 (1.17)
	vPO + TCA1 (200 : 200)		> 95 % | > 95 % (3d)	4,600 (1.12)
	vPO + TCA1 (400 : 200)		> 95 % | > 95 % (3d)	6,000 (1.12)
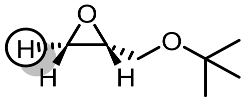<br>tert-Butyl glycidyl ether	BGE (200 equiv.)	2.64-2.60 (m)<br>1.04-1.00 (m)	83 % (5 d)	1,700 (1.08)
	BGE + TCA1 (200 : 200)		81 % | 83 % (3d)	5,000 (1.11)
	BGE + TCA1 (400 : 200)		> 95 % | > 95 % (5 d)	6,600 (1.10)
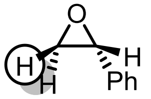<br>Styrene oxide	SO (200 equiv.)	3.16-3.03 (m)<br>3.07-3.00 (m)	27 % (5 d)*	400 (1.06)
	SO + TCA1 (200 : 200)		95 % | 93 % (3 d)	4,800 (1.12)
			90 % | > 95 % (5 d)	5,200 (1.11)
	SO + TCA1 (400 : 200)		50 % | > 95 % (5 d)*	5,200 (1.14)
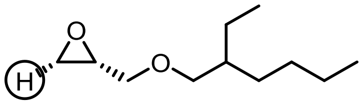<br>2-Ethylhexyl glycidyl ether	EHGE (200 equiv.)	2.64-2.59 (dd) or 2.88-2.75 (dd)<br>1.04-1.00 (m)	> 95 % (5d)	3,000 (1.08)
	EHGE + TCA1 (200 : 200)		> 95 % | > 95 % (3d)	6,800 (1.11)
	EHGE + TCA1 (400 : 200)		> 95 % | > 95 % (5 d)	10,100 (1.11)
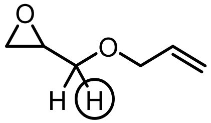<br>Allyl glycidyl ether	AGE + TCA1 (200 equiv.)	3.45-3.35 (m)<br>1.04-1.00 (m)	> 95 % (5d)	2,000 (1.07)
	AGE + TCA1 (200 : 200)		> 95 % | > 95 % (3d)	5,000 (1.12)
	AGE + TCA1 (400 : 200)		> 95 % | > 95 % (5d)	7,600 (1.11)
PO	PO + TCA1 (200 equiv.)	3.25-3.14<br>2.79-2.73	> 95 % (5d)	1,400 (1.10)
	PO + TCA1 (200 : 200)		93 % | > 95 % (3d)	4,700 (1.10)
	PO + TCA1 (400 : 200)		> 95 % | > 95 % (5 d)	6,000 (1.11)

*Low conversion is sometimes observed for styrene oxide due to catalyst deactivation.

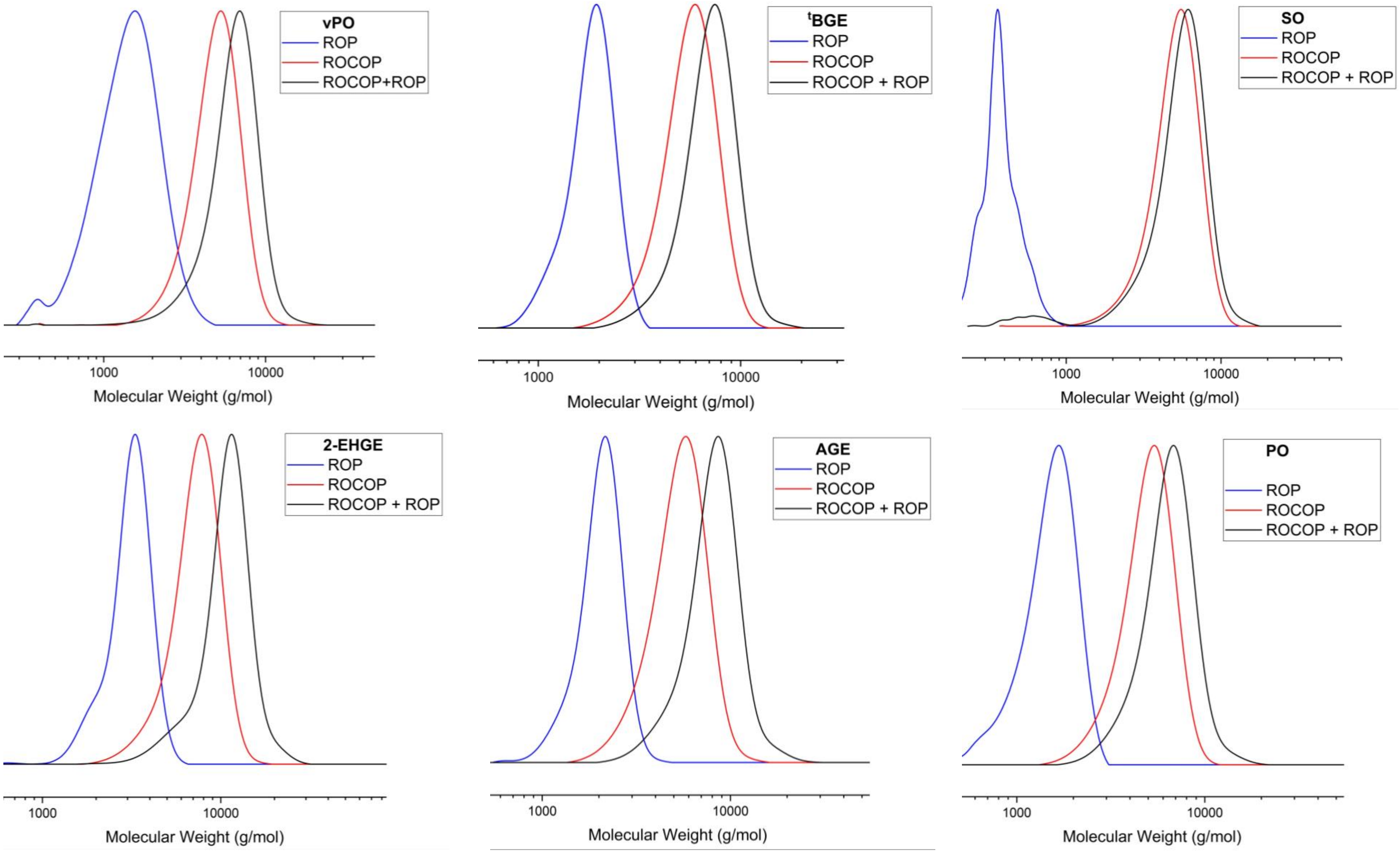

Supplementary Figure 15 – GPC traces of polyethers (blue), polyesters (Red) and polyester-*b*-polyethers (black) with different PO derivatives.

Supplementary Table 5 – Anhydride Scope.

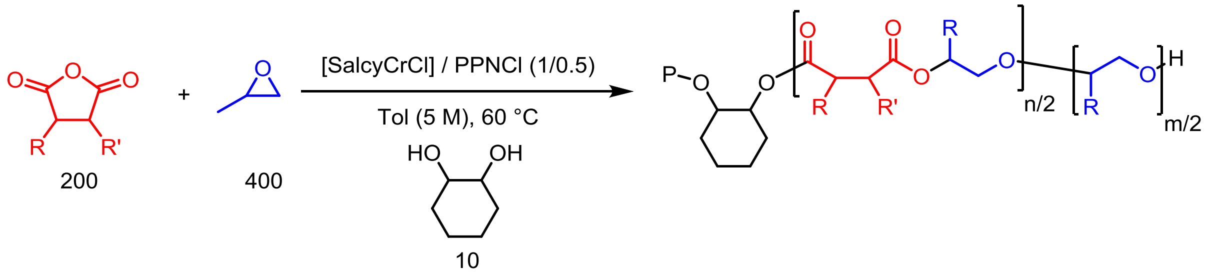

Anhydride	Shift Anhydride (^{1}H NMR)	Time (d)	Conversion		M_n ($Đ$)
			Anhydride	PO[b]	
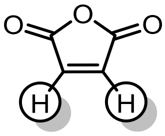	7.05 – 6.95 ppm	3 d	99 %	63 %	5,800 (3.11)
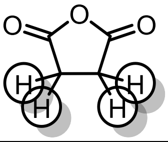	3.07 – 2.91 ppm	3 d	99 %	> 95 %	5,400 (1.85)
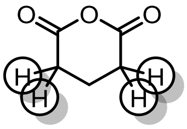	2.05 – 2.00 ppm*	3 d	> 95 %	> 95 %	1,700 (1.54)
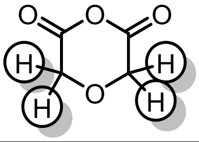	4.48 – 4.35 ppm	3 d	99 %	> 95 %	2,100 (2.14)
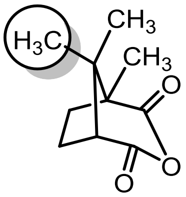	1.02 – 0.98 ppm	3 d	82 %	> 95 %	6,100 (1.18)
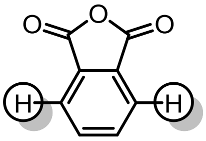	8.05 – 7.99 ppm	3 d	99 %	> 95 %	7,800 (1.54)
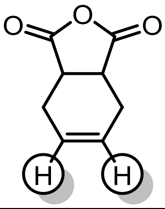	6.04 – 5.92 ppm#	3 d	99 %	> 95 %	5,100 (1.34)

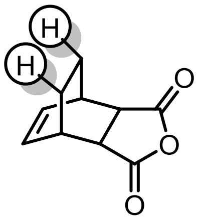	1.46 – 1.41 ppm	3 d	> 95 %	> 95 %	5,700 (1.20)
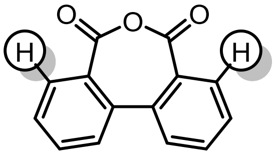	7.77 – 7.68 ppm[#]	3 d	99 %	> 95 %	5,800 (1.19)

[b] Conversion based on integrals in ^{1}H NMR spectrum at δ = 3.03 – 2.97 ppm of crude mixture and given with respect to the formation of polyether (i. e. 100 * 1 –Integral (t=x) / (Integral (t=0) * 0.5))

* Partial overlap of anhydride and polyester signals.
# Integral between δ = 2.80 – 2.73 ppm used for PO conversion.

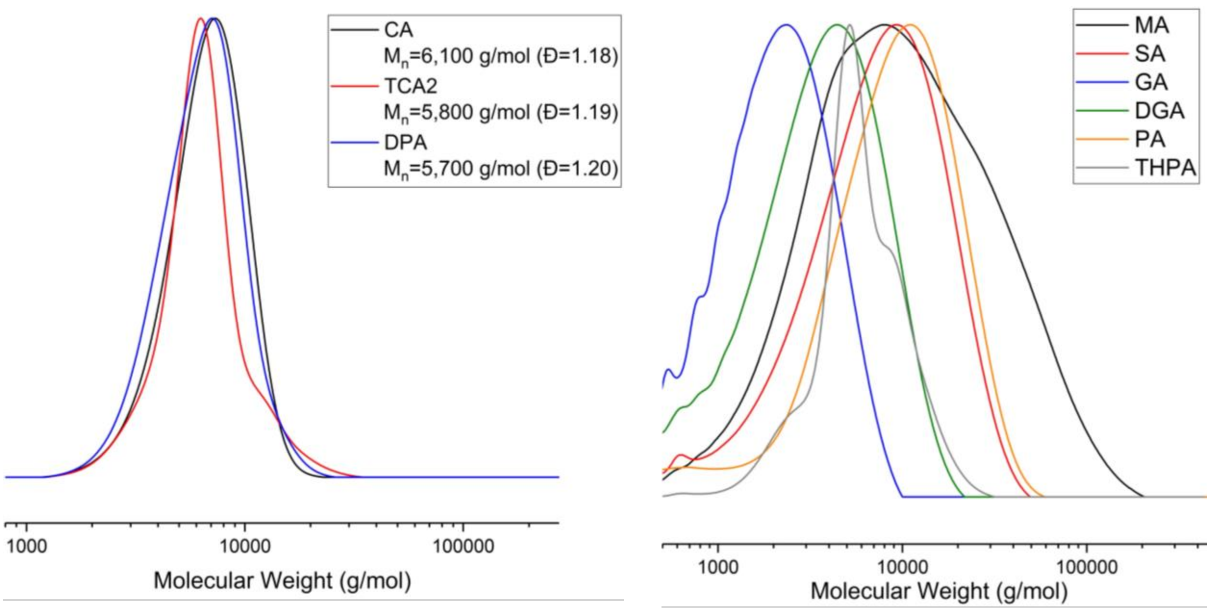

Supplementary Figure 16 – GPC traces of the one-pot polymerization of different anhydrides with PO.

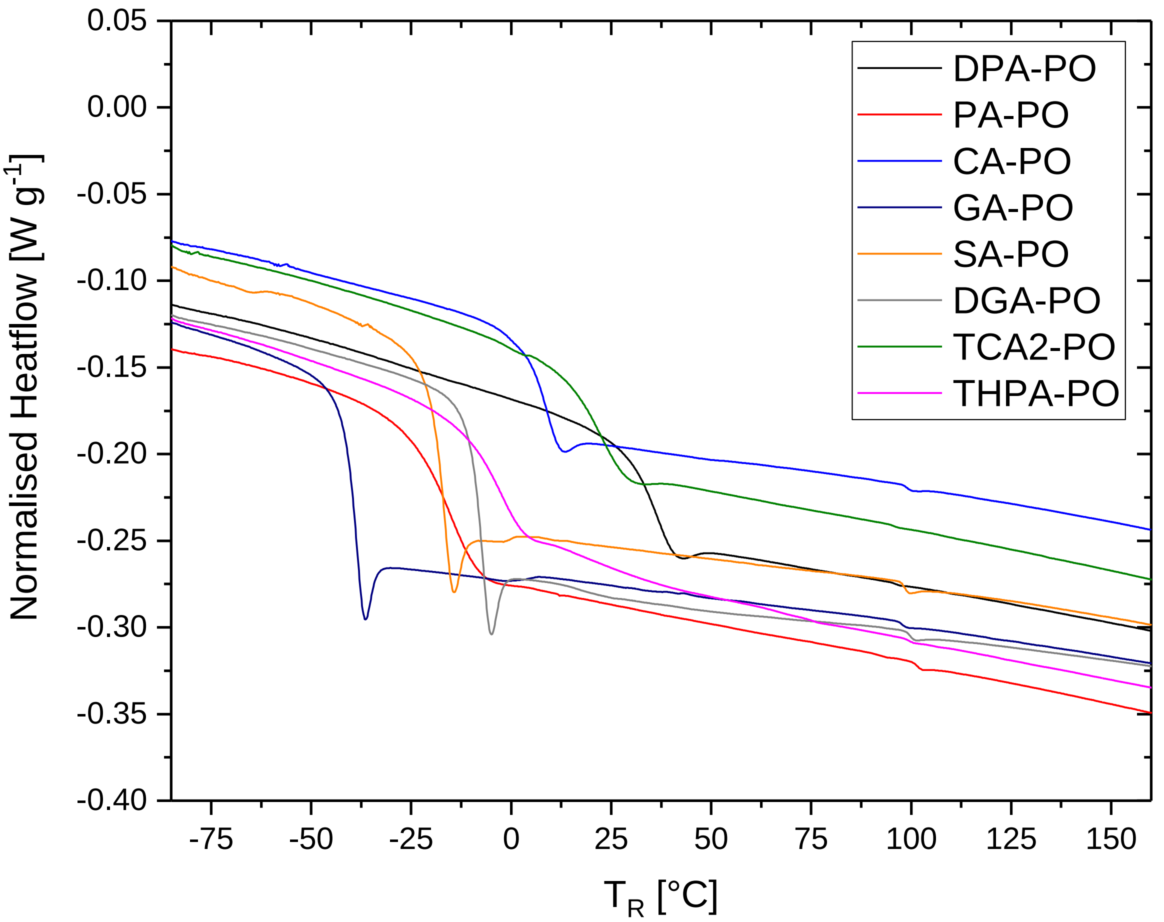

Supplementary Figure 17 – DSC thermograms for polymers based on different anhydrides and PO.

Supplementary Table 6 – Switch catalysis with PO and TCA1 in the presence of different chain-transfer agents.

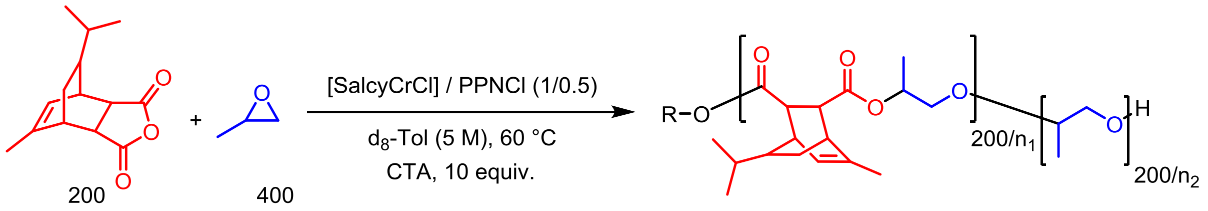

CTA (10 equiv.)[1]	Rct-time	Conversion[%][2] PO | TCA1	PE:PPO [mol%][3]	M_n ($Đ$) [g mol^{-1}][4]	T_g [°C][5]
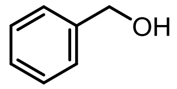 Benzyl alcohol	3d	88 % | 99 %	53:47	5,900 (1.08)	33 °C
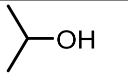 *iso*-Propanol	3d	89 % | 99 %	53:47	7,000 (1.07)	21 °C
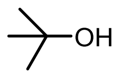 *tert*-Butanol	3d	97 % | 99 %	68:32	59,000 (1.02) 26,000 (1.09)	61 °C

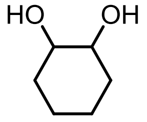 *trans*-1,2-Cyclohexanediol	5d	90 % | 99 %	53:47	6,700 (1.12)	41 °C
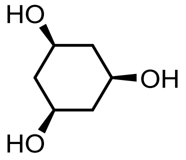 *cis*,*cis*-1,3,5-Cyclohexanetriol	3d	83 % | 99 %	55:45	6,600 (1.14)	42 °C
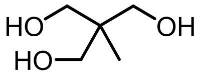 1,1,1-Tris(hydroxymethyl)ethane	3d	87 % | 99 %	53:47	7,100 (1.11)	34 °C
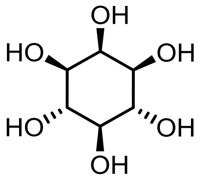 *myo*-Inositol	3d	83 % | 99 %	75:25	26,800 (1.09) 10,100 (1.07)	63 °C

[1]Molar ratio with respect to catalyst. [2]Based on ^{1}H NMR spectroscopy. The following chemical shifts were used: 1.04-1.00 ppm (TCA1), 3.72-3.25 ppm (PPO; the conversion was calculated based on the theoretical maximum conversion of PO). [3]Based on ^{1}H NMR spectroscopy of crude sample. PE content was calculated based on shifts for PE (5.85-5.55, HC=C) and PPO (3.72-3.25 ppm), i. e. Integral PE/(Integral PE+Integral PPO/3). [4]Measured by GPC (30°C, THF) with narrow polystyrene standards. [5]Measured by DSC, 3rd heating cycle.

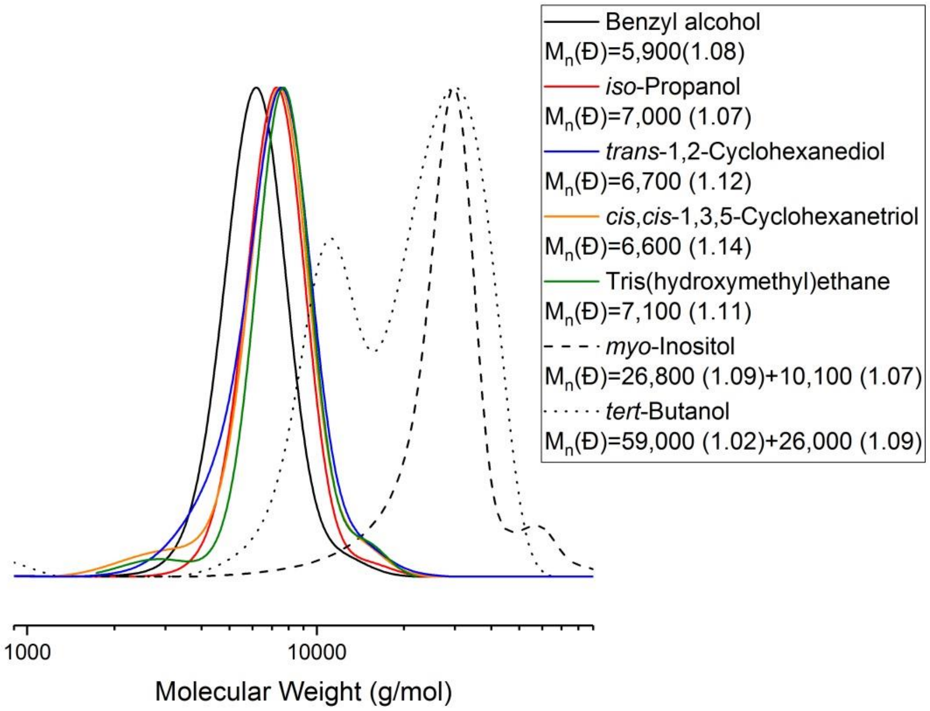

Supplementary Figure 18 – GPC traces of TCA1/PO switch catalysis with selected alcohols.

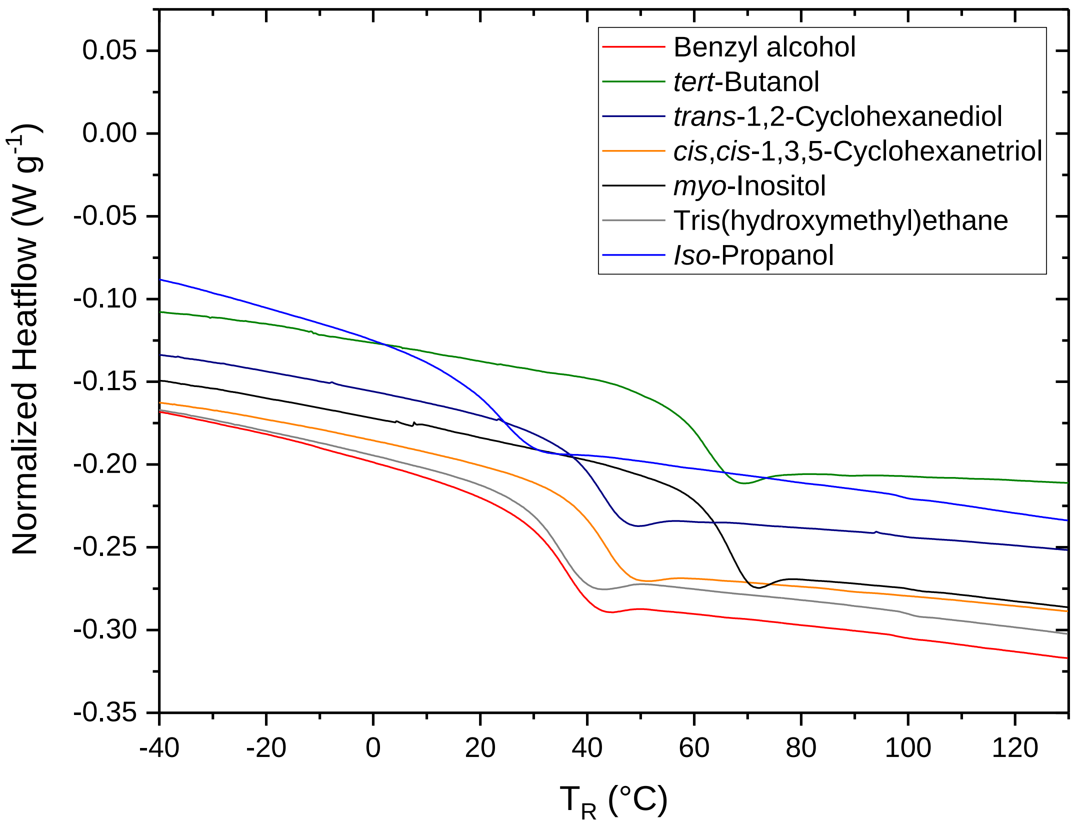

Supplementary Figure 19 – DSC thermograms of TCA1/PO switch catalysis with selected alcohols.

Supplementary Table 7 – Multiple Monomer Additions.

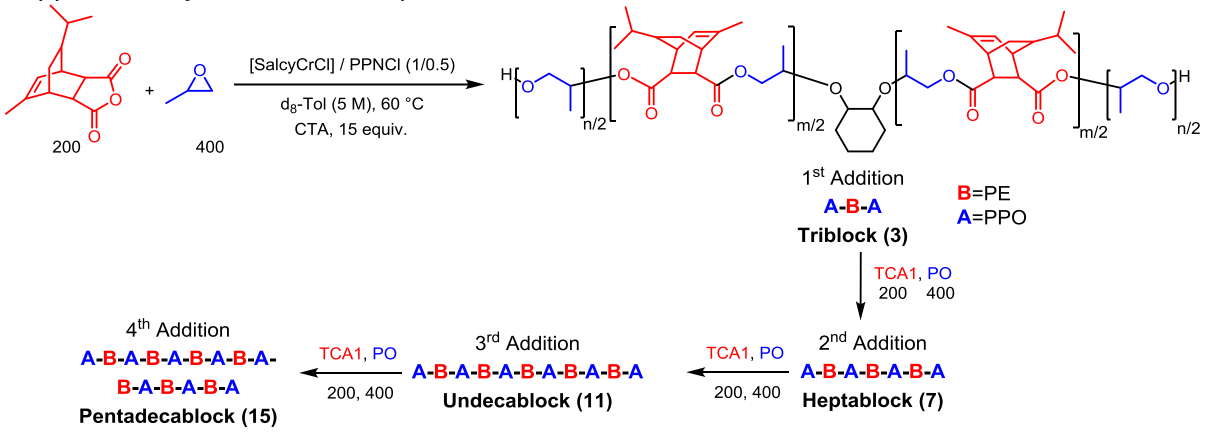

No of addition of TCA1/PO	No of Blocks	Rct-time	Conversion[%][2] PO | TCA1	M_n ($Đ$) [g mol^{-1}][4]	T_g [°C]
1	3	3 d	93 % | 99 %	4,900 (1.12)	N. D.
2*	7	3 d	> 95 % | 99 %	7,900 (1.15)	N. D.
3	11	5 d	> 95 % | 99 %	13,300 (1.26)	N. D.
4	15	5 d	> 95 % | 99 %	17,800 (1.38)	24 °C

* 0.5 mL of toluene added.

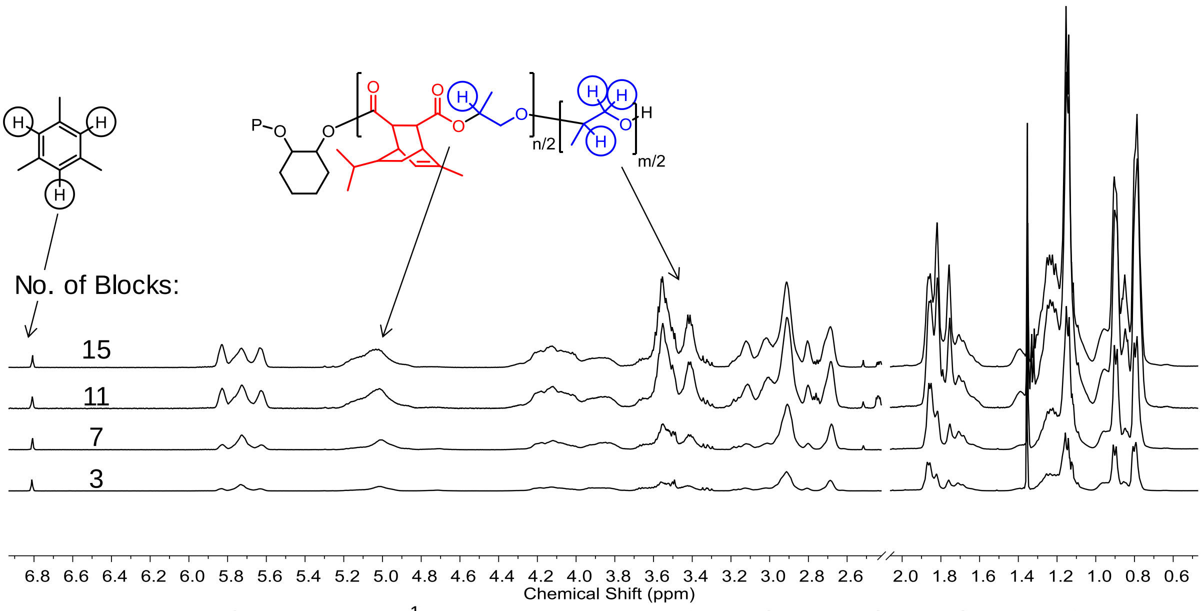

Supplementary Figure 20 – ^{1}H NMR spectra of crude reaction mixture of one-pot polymerization of TCA1/PO after each addition of the monomer mixture.

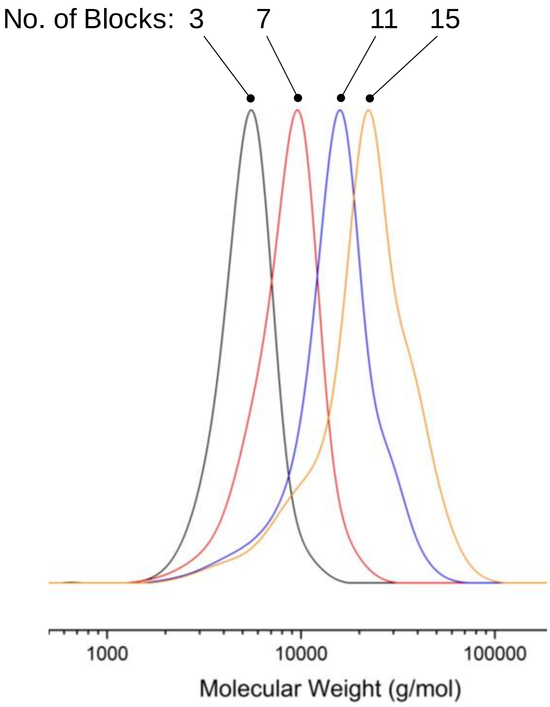

Supplementary Figure 21 – GPC traces of crude reaction mixture of one-pot polymerization of TCA1/PO after each addition of the monomer mixture.

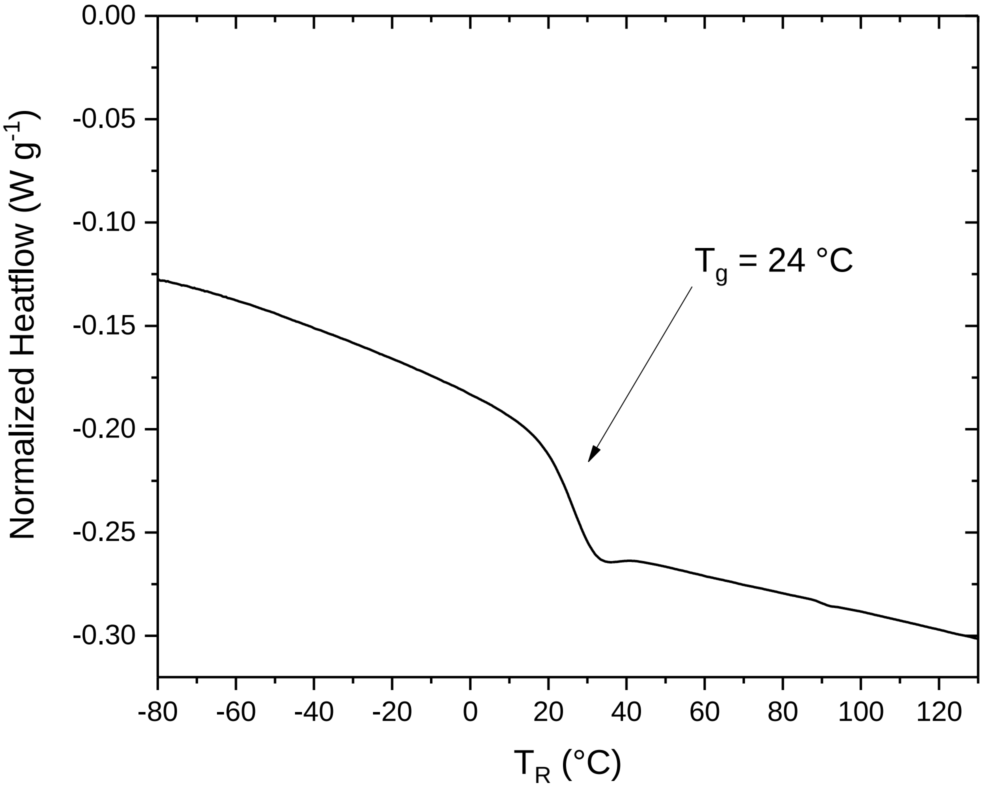

Supplementary Figure 22 – DSC thermogram of multiblock copolymer based on TCA1/PO.

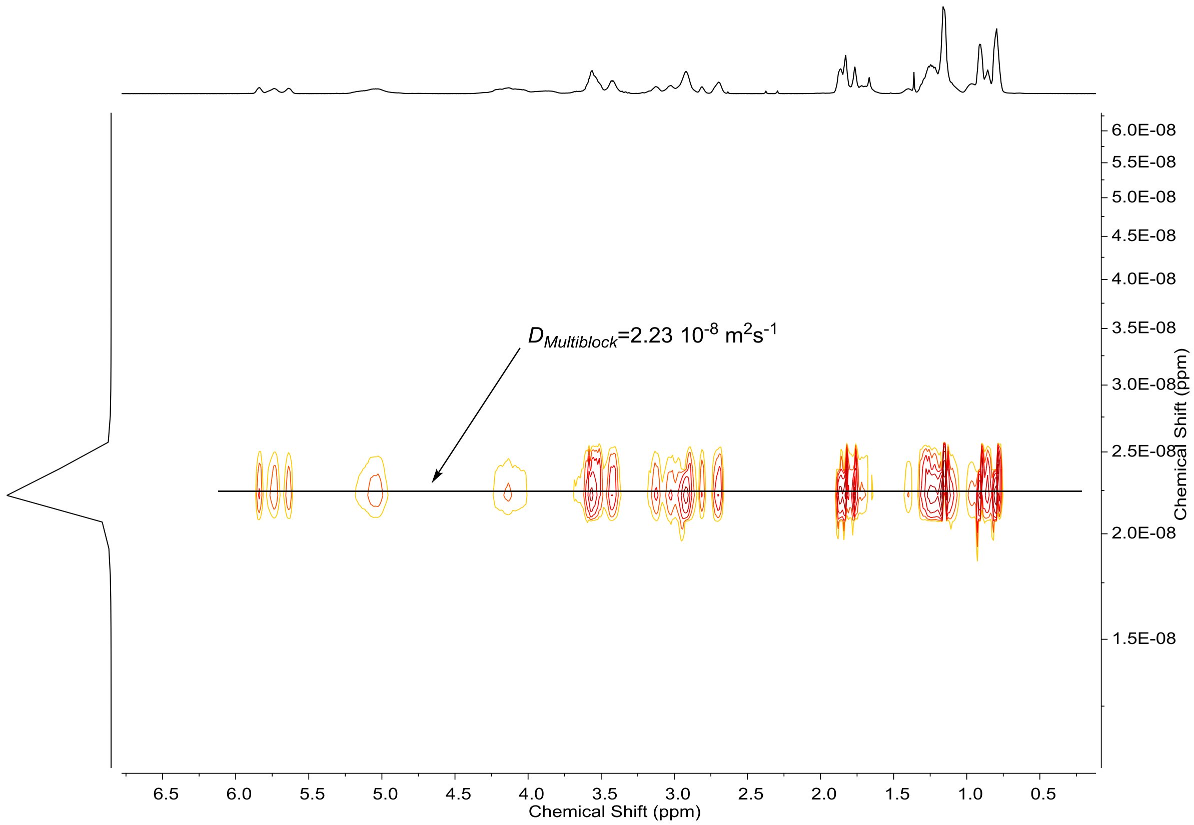

Supplementary Figure 23 – ^{1}H DOSY NMR Spectrum of the multiblock copolymer after purification. Only one diffusion coefficient was found, indicating that all blocks are attached.

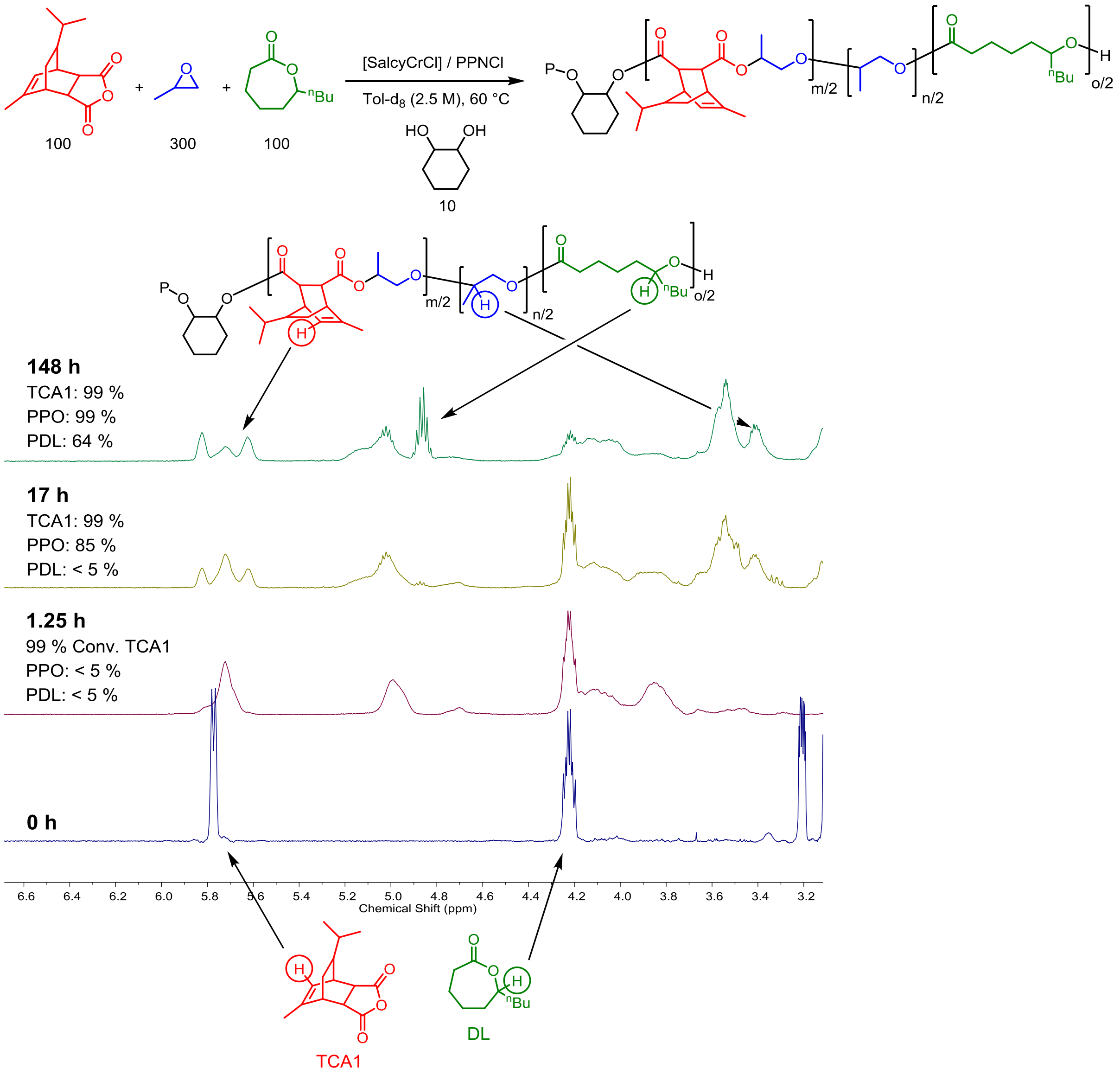

Supplementary Figure 24 – ^{1}H NMR spectra at selected points throughout the one-pot polymerization of TCA1/PO/DL.

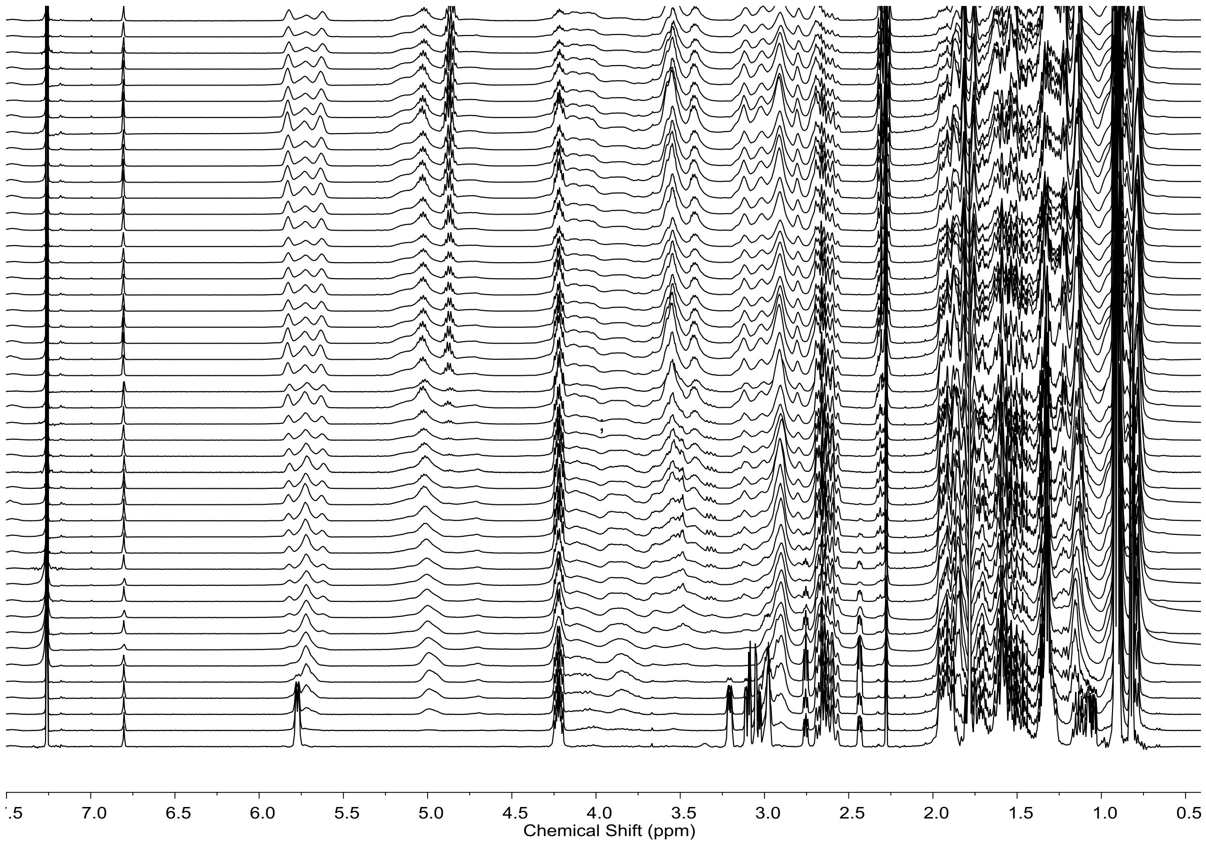

Supplementary Figure 25 – Full list of ^{1}H NMR spectra used for monitoring of TCA1/PO/DL.

Supplementary Table 8 – Kinetic data for the one-pot polymerisation of TCA1/PO/DL.

Time [h]	Integral TCA1[1]	Integral PPO[2]	Integral PDL[3]	Integral DL[4]	TCA1 Remaining [%]	PPO Formed [%]	DL Remaining [%][5]
0	13.3	0	0	14.5	100	0.0	100
0.25	11.6	0	0	13.5	88	0.0	100
0.5	10.2	0	0	13.6	77	0.0	100
0.75	8.8	0	0	11.9	66	0.0	100
1	7.7	3	0	14.8	58	5.4	100
1.5	4.7	3	0	15.6	35	6.1	100
2	2.6	5	0	15.4	20	9.5	100
2.5	1.1	6	0	15.7	8	12.5	100
3	0.2	8	0	15.9	2	17.2	100
5	0.1	29	0	16.9	1	59.6	100
7	0.0	38	0	16.8	0	77.4	100
8	0.0	40	0	15.3	0	81.7	100
9	0.0	39	0.73	14.2	0	79.7	95
10	0.0	41	0.78	14.0	0	82.8	95
11	0.0	41	0.82	14.0	0	82.6	94
12	0.0	40	0.78	13.4	0	81.6	94
13	0.0	42	0.93	13.5	0	85.2	94
16	0.0	--	1.21	16.0	0	--	93
19	0.0	46	1.59	14.0	0	94.4	90
35	0.0	46	3.23	10.4	0	93.6	76
41.3	0.0	46	4.2	9.5	0	93.9	69
55.3	0.0	48	5.94	7.2	0	97.6	55
63.5	0.0	45	6.21	6.5	0	91.2	51
78.1	0.0	48	8.31	5.2	0	97.1	38
88.5	0.0	45	8.52	4.3	0	92.3	33
102.2	0.0	45	9.42	3.5	0	92.1	27
111.5	0.0	45	9.52	3.2	0	91.8	25
126.9	0.0	48	10.35	3.1	0	98.0	23
148.6	0.0	47	10.55	2.7	0	95.0	20
159	0.0	49	11.21	2.7	0	100.0	19
177	0.0	44	10.11	2.2	0	89.4	18

[1] 3.22-3.15 ppm; [2] 3.68-3.25 ppm; [3] 4.89-4.79 ppm; [4] 4.24-4.16 ppm; [5] Partial overlap of signals during 1.25-5 h.

Supplementary note 1: Reactivity Ratios

Reactivity ratios were estimated by the Fineman-Ross method.[5]

$$G_{PO} = H_{PO} r_{PO} - r_{DL} \quad G = \frac{f_{PO}(2F_{PO}-1)}{(1-f_{PO})F_{PO}} \quad H = \frac{f_{PO}^2(1-F_{PO})}{(1-f_{PO})^2 F_{PO}} \qquad (1)$$

Where f_{PO} is the initial feed ratio of PO in the reaction mixture and F_{PO} is the composition of PO in the copolymer. At all feed ratios, the conversion of DL was <10% and PO <20%.

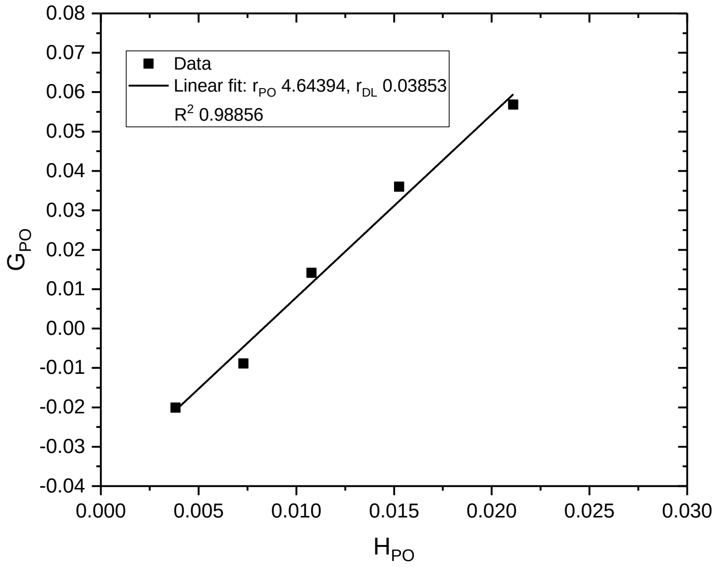

Supplementary Figure 26 – Estimation of reactivity ratios for PO and DL by Fineman-Ross method.

The Meyer-Lowry model was also used to estimate the reactivity ratios at conversions <30% by fitting the compositional drift in f_{PO} to the total conversion X_n.[6]

$$\chi_n = 1 - \left(\frac{f_1}{f_{10}}\right)^{\alpha} \left(\frac{f_2}{f_{20}}\right)^{\beta} \left(\frac{f_{10}-\delta}{f_1-\delta}\right)^{\gamma} \qquad (2)$$

$$\alpha = \frac{r_2}{(1-r_2)};\ \beta = \frac{r_1}{(1-r_1)};\ \gamma = \frac{1-r_1 r_2}{(1-r_1)(1-r_2)};\ \delta = \frac{(1-r_2)}{(2-r_1-r_2)} \qquad (3)$$

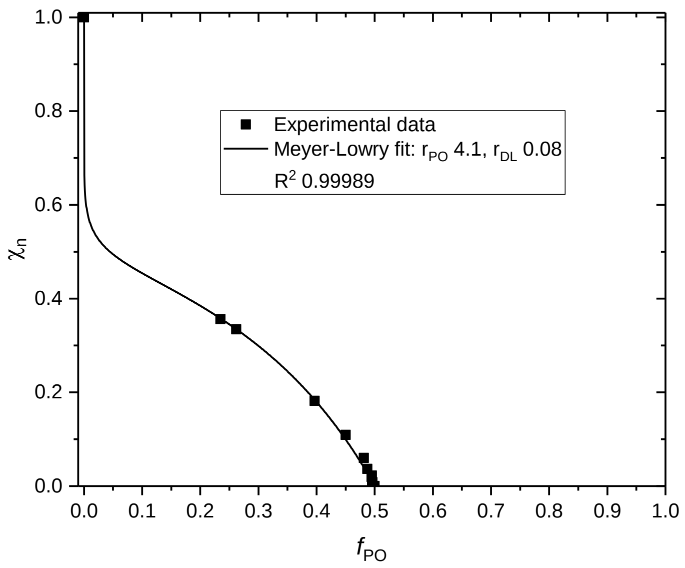

Supplementary Figure 27 – Meyer-Lowry fit to PO/DL copolymerisation data.

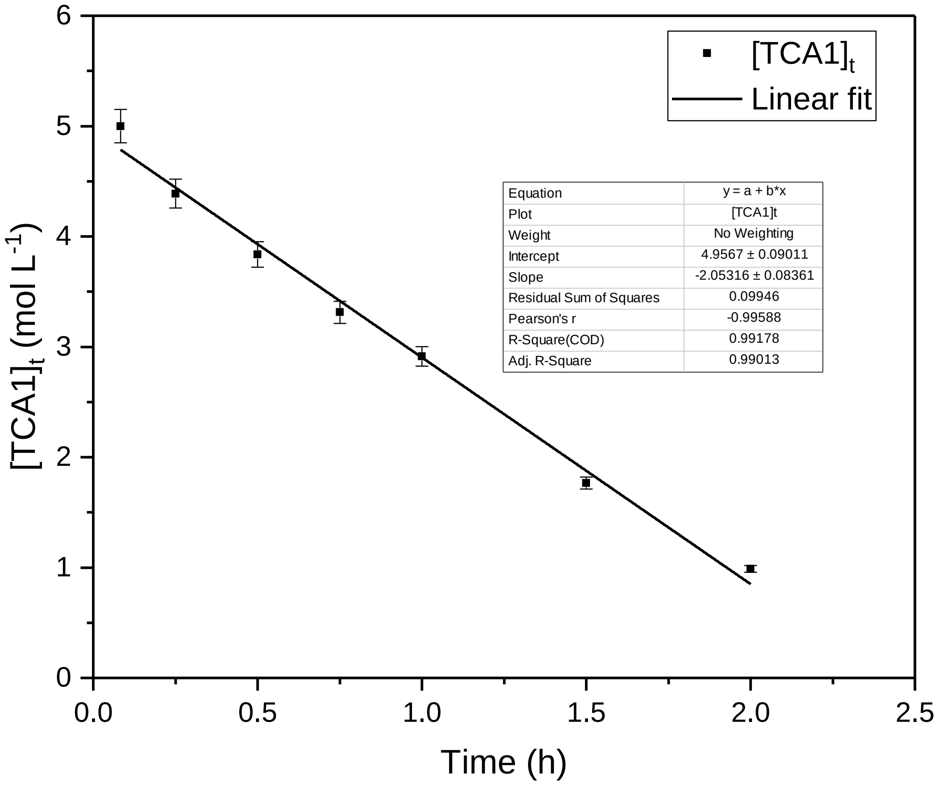

Supplementary Figure 28 – Estimation of rate coefficient for TCA1/PO ROCOP. N.B. The estimated rate constants are different from the previous switch catalysis with TCA1/PO (Supplementary Figures 4 and 5) due to different reaction conditions.

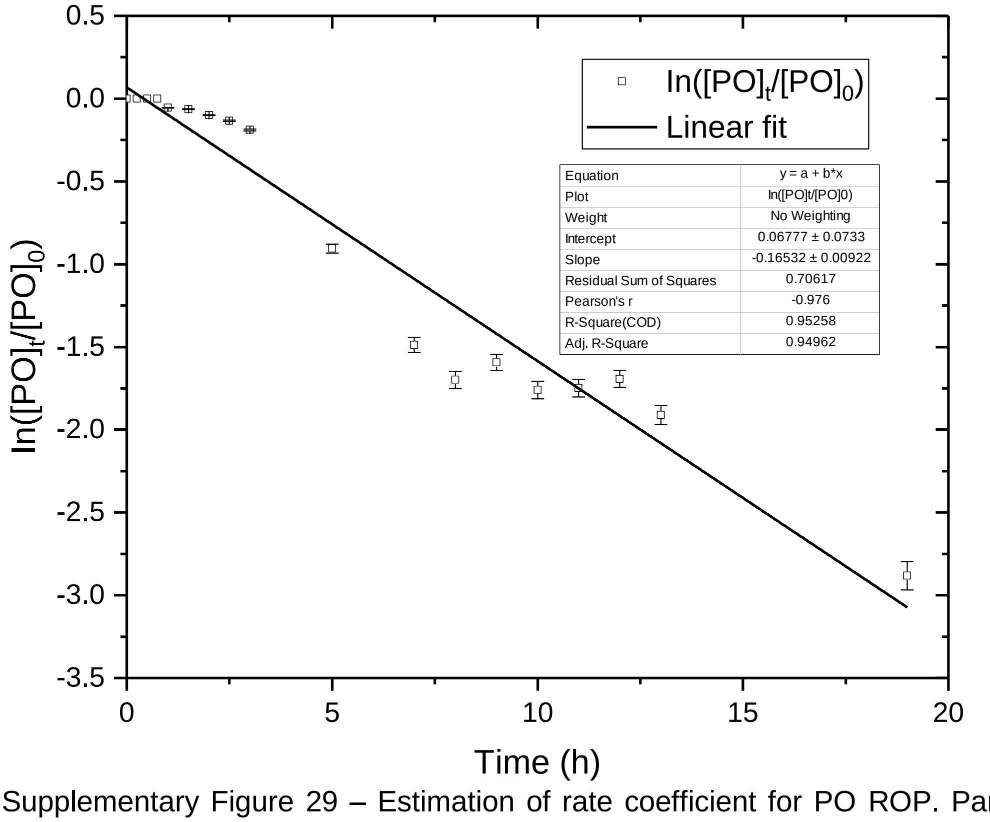

Supplementary Figure 29 – Estimation of rate coefficient for PO ROP. Partial overlap with DL reduced the quality of fit up to 5 hours, which is why only a rough estimate can be provided here. For kinetic analysis of PO ROP, see Supplementary Figure 5.

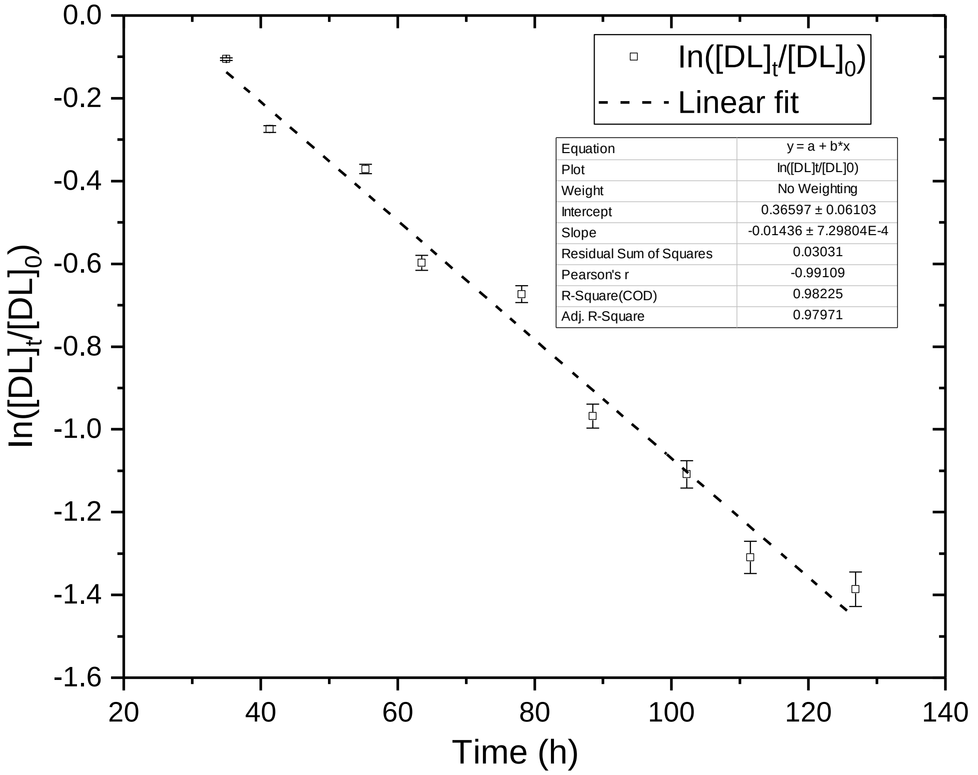

Supplementary Figure 30 – Estimation of rate coefficient for DL ROP.

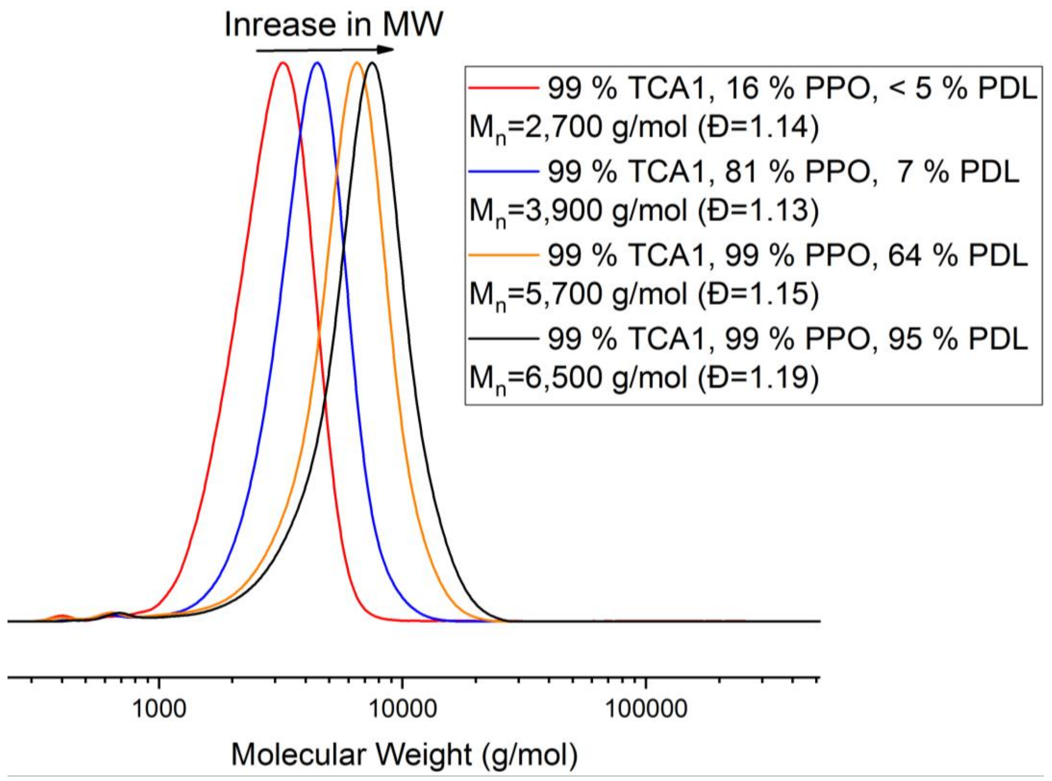

Supplementary Figure 31 – Analysis of aliquots from one-pot polymerization of TCA1/PO/DL by GPC.

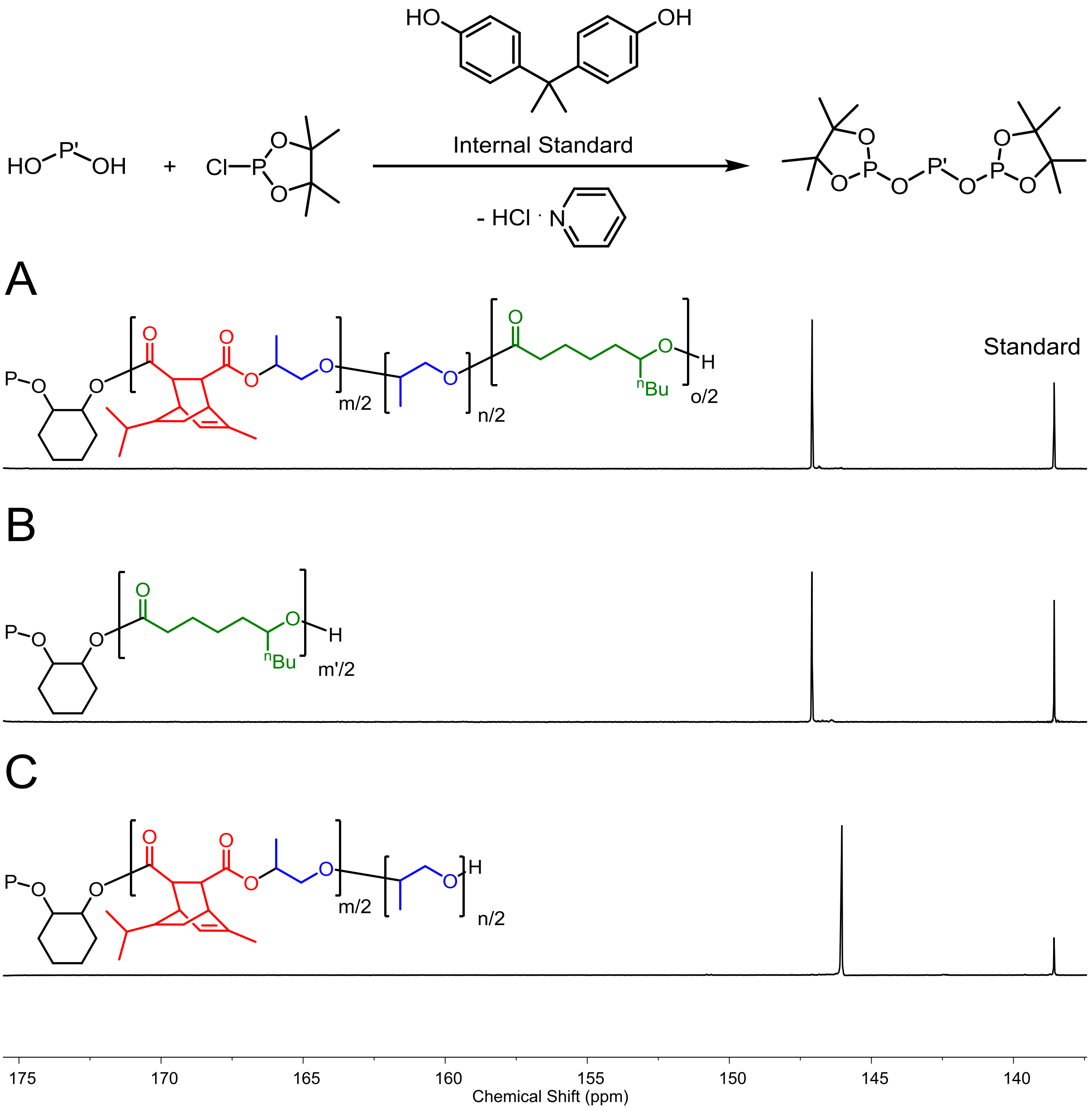

Supplementary Figure 32 – $^{31}P\{^1H\}$ NMR Spectrum of proposed pentablock copolymer obtained from the one-pot polymerization of TCA1, PO and DL (A, top), PDL (B, middle) and the triblock copolymer obtained from TCA1 and PO (C, bottom) the after the reaction with 2-chloro-4,4,5,5-tetramethyl-1,3,2-dioxaphospholane. For A, only one peak at 147.1 ppm was found, which was in agreement with the PDL sample.

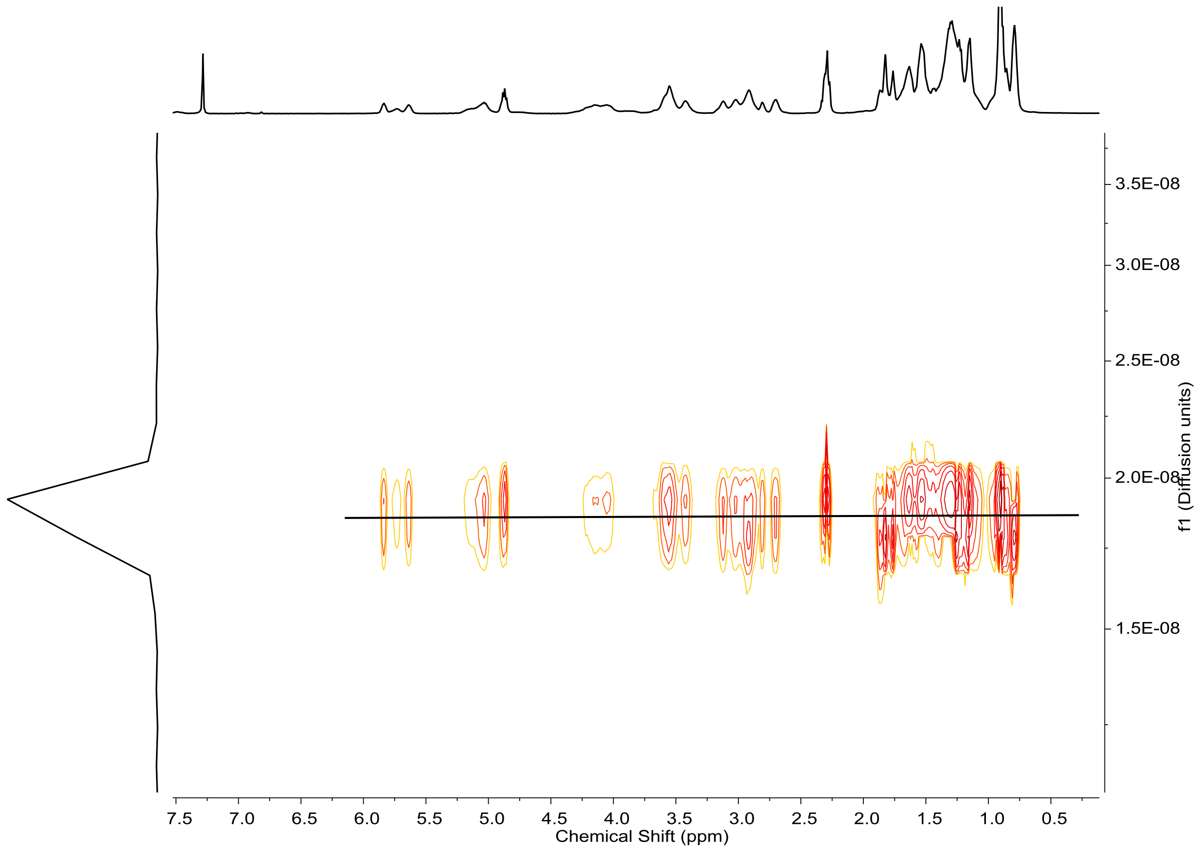

Supplementary Figure 33 – DOSY NMR spectrum of block copolymer obtained from TCA1, PO and DL.

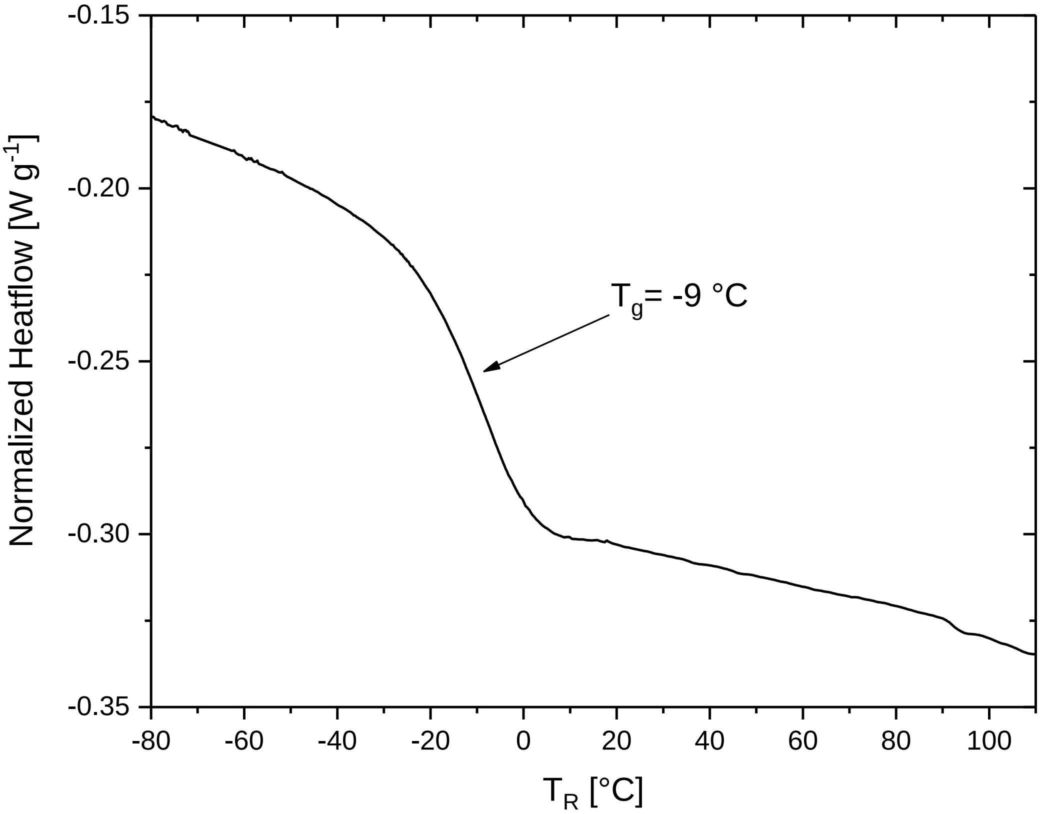

Supplementary Figure 34 – DSC thermogram of polymer obtained from PO/TCA1/DL.

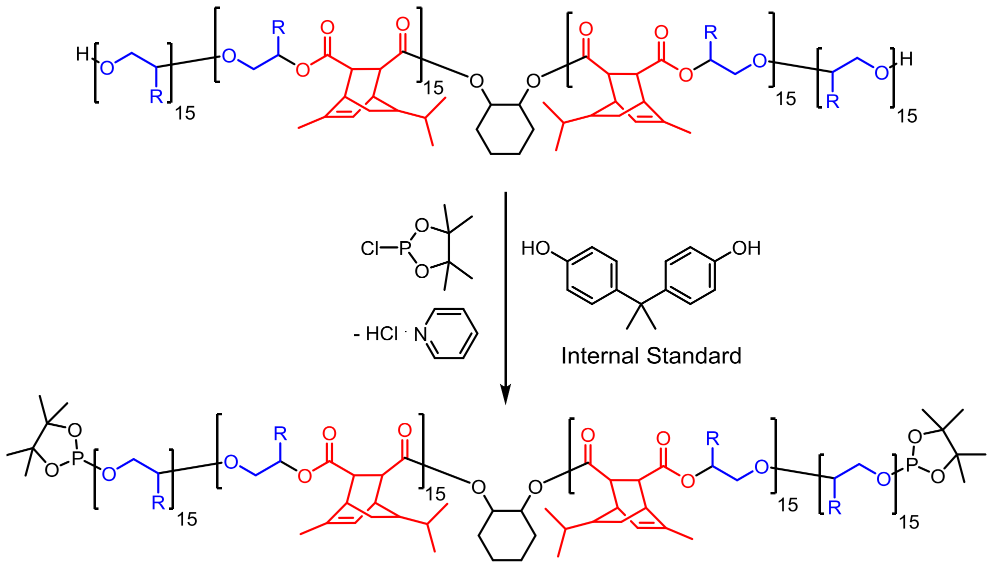

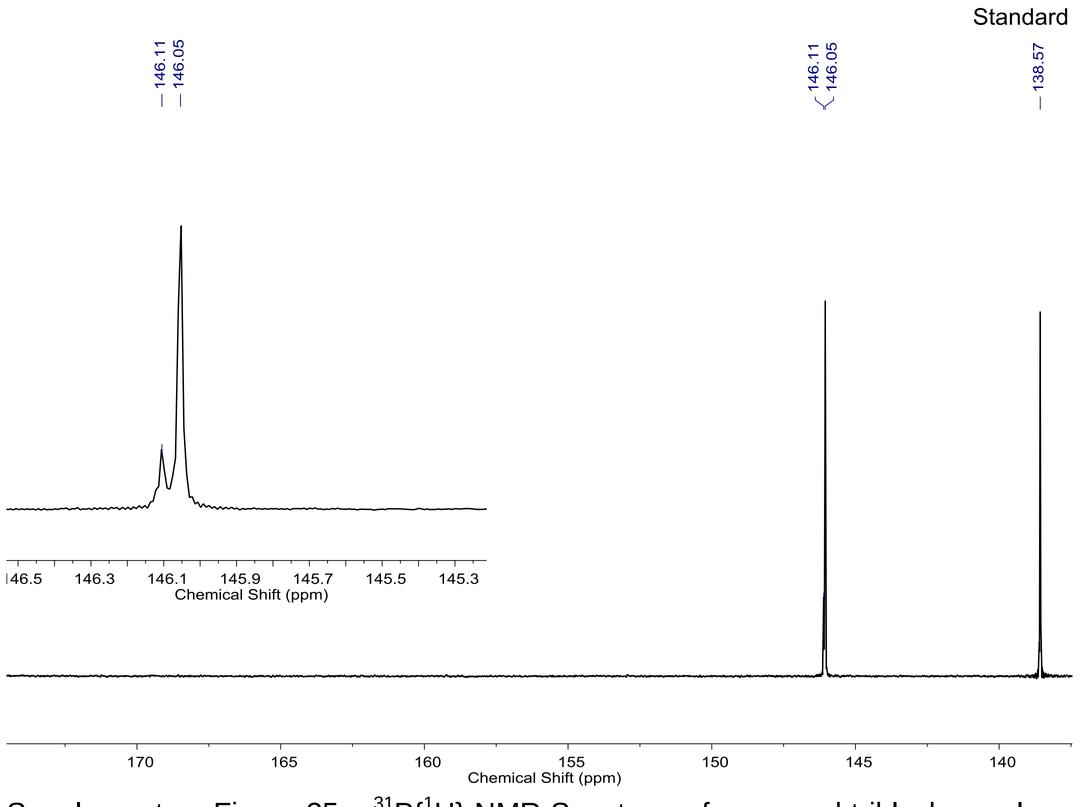

Supplementary Figure 35 – $^{31}P\{^{1}H\}$ NMR Spectrum of proposed triblock copolymer obtained from the one-pot polymerization of TCA1 and PO after the reaction with 2-chloro-4,4,5,5-tetramethyl-1,3,2-dioxaphospholane.

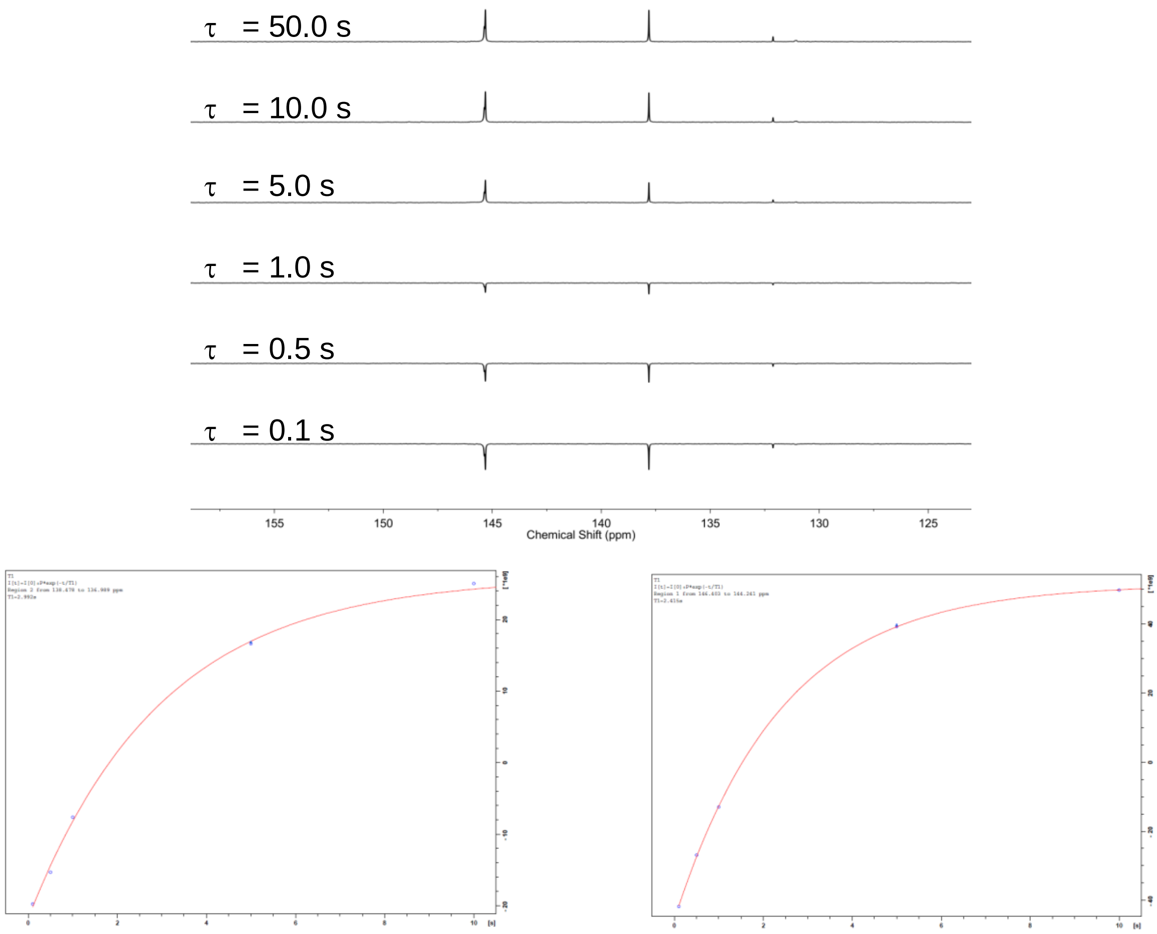

Supplementary Figure 36 – T_1-Inversion recovery experiment for peaks at 138 and 146 ppm in $^{31}P\{^1H\}$ NMR spectrum. T_1 values were determined as 2.992 s and 2.415 s after exponential fitting (I[t]=I[0]+P*exp(-t/T1)) for 138 and 144 ppm, respectively.

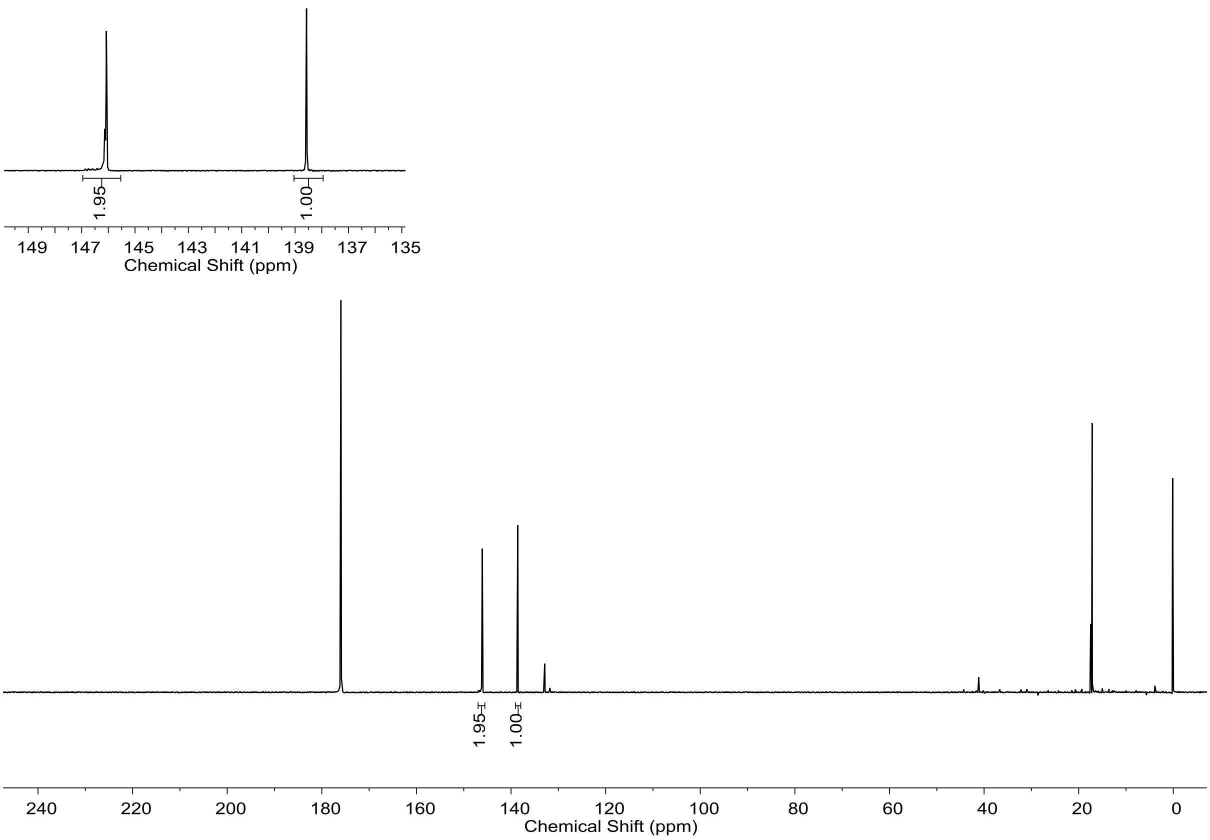

Supplementary Figure 37 – $^{31}P\{^{1}H\}$ NMR spectrum of block copolymer with D_1 = 5 * 2.992 s (as determined Supplementary Figure 36; 128 scans). Based on this integration, 27.3 µmol [OH] / 50.0 mg [Polymer] are present (calculated from standard, the integral 1.00 corresponds to 40 µL of a stock solution of 40 mg / mL (1.6 mg), which, in turn is equivalent to 14.0 µmol of [OH]).

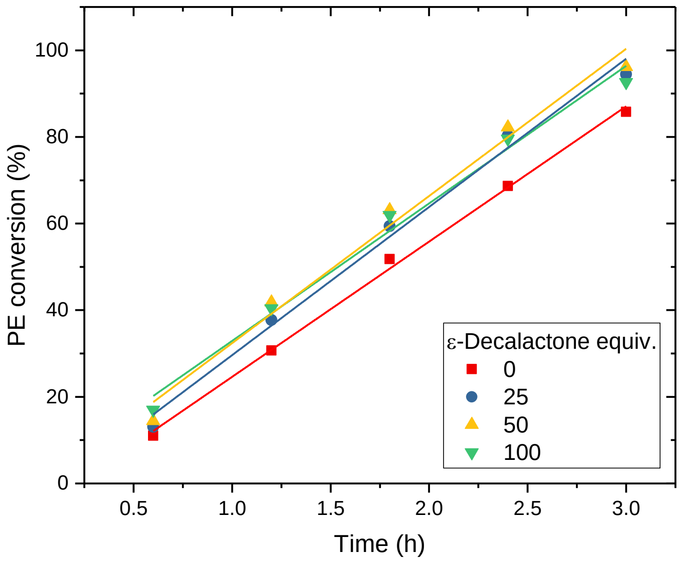

Supplementary Figure 38 – Plot of polyester (TCA1/PO copolymer) conversion against time as a function of ε-decalactone equivalents.*

*A minimal solvent effect is observed, with the presence of ε-decalatone only slightly enhancing the rate of TCA1/PO copolymerization. The data do not provide evidence for the rate enhancement observed and this is instead attributed to catalyst concentration. However, it should be noted that solvent effects have been observed in other polymerisation systems.[7]

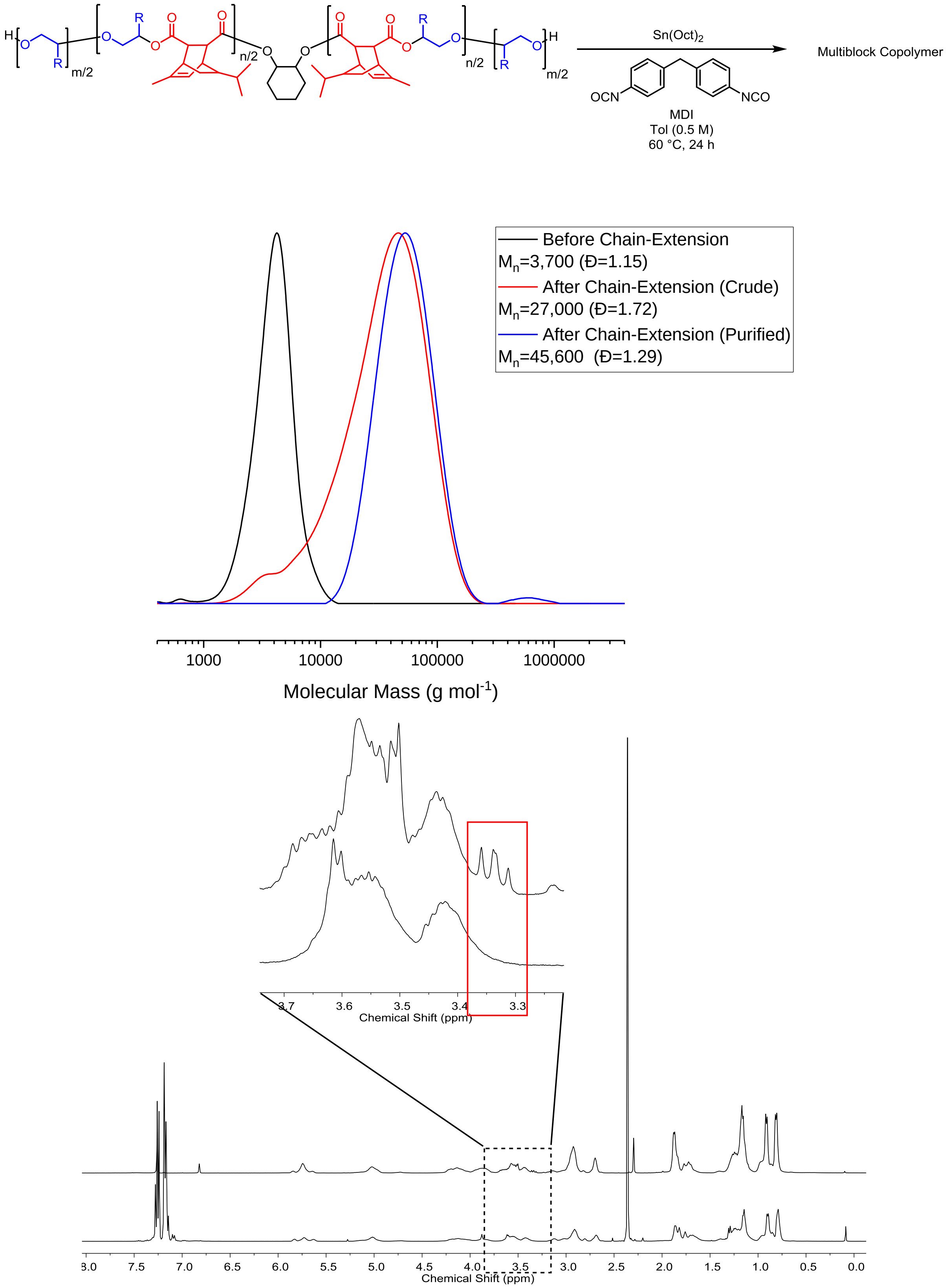

Supplementary Figure 39 – GPC traces (top) and ^{1}H NMR spectra before and after chain-extension.

Supplementary Table 9 – Block Copolymers based on TCA1/PO with different block ratios.

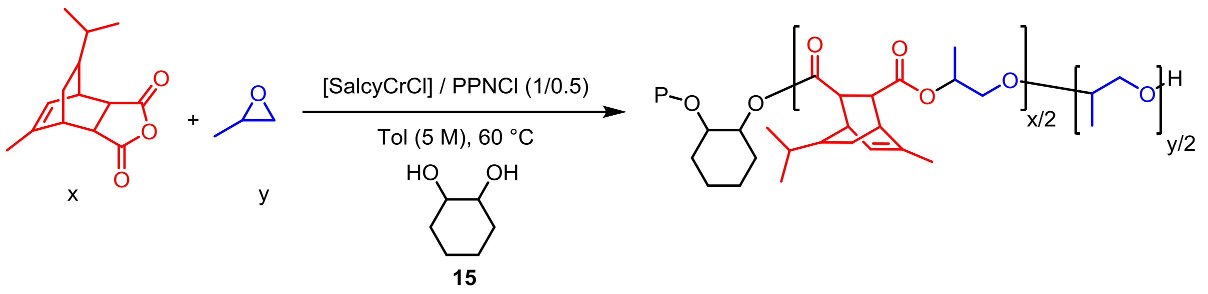

TCA1:PO (equiv.)	Rct-time	Conversion[2] PO | TCA1	PE:PPO [wt%][3]	M_n ($Đ$) [g mol^{-1}][4]	T_g [°C][5]
1026:1020	10 d	> 95 % | 99 %	99	40,900 (1.02) 18,300 (1.07)	73
821:1854	10 d	> 95 % | 99 %	80	22,800 (1.49)	34
718:2268	10 d	> 95 % | 99 %	70	24,100 (1.56)	-3
615:2682	10 d	> 95 % | 99 %	60	18,600 (1.56)	-1
513:3096	10 d	> 95 % | 99 %	50	17,200 (1.55)	-12
410:3510	10 d	> 95 % | 99 %	40	15,100 (1.57)	-42
308:3924	10 d	82 % | 99 %	27	14,700 (1.50)	N. D.
	15 d	87 % | 99 %		15,900 (1.49)	-55 °C
205:4337	10 d	65 % | 99 %	16	12,600 (1.49)	N. D.
	15 d	85 % | 99 %		16,100 (1.36)	-61 °C
103:4751	10 d	70 % | 99 %	6	13,600 (1.28)	N. D.
	15 d	88 % | 99 %		16,500 (1.31)	-66 °C
50:5124	10 d	82 % | 99 %	1	18,800 (1.04) 7,200 (1.07)	N. D.
	15 d	85 % | 99 %		20,300 (1.04) 7,200 (1.11)	-68 °C

[1]Molar ratio with respect to catalyst. [2]Based on ^{1}H NMR spectroscopy. The following chemical shifts were used: 1.04-1.00 ppm (TCA1), 3.72-3.25 ppm (PPO; the conversion was calculated based on the theoretical maximum conversion of PO). [3]Based on ^{1}H NMR spectroscopy of crude sample. PE content was calculated based on shifts for PE (5.85-5.55, HC=C) and PPO (3.72-3.25 ppm), i. e. Integral PE/(Integral PE+Integral PPO/3). [4]Measured by GPC (30°C, THF) with narrow polystyrene standards. [5]Measured by DSC, 2nd or 3rd heating cycle. [6]Determined by ratio of PO:PPO (3.02-2.97 ppm, H^{PO}, vs. 3.73-3.20 ppm, HPPO; i. e. Integral H^{PPO}/(H^{PPO}+H^{PO}*3).

* Weak Transition.
# Soluble in Pentane (not isolated).

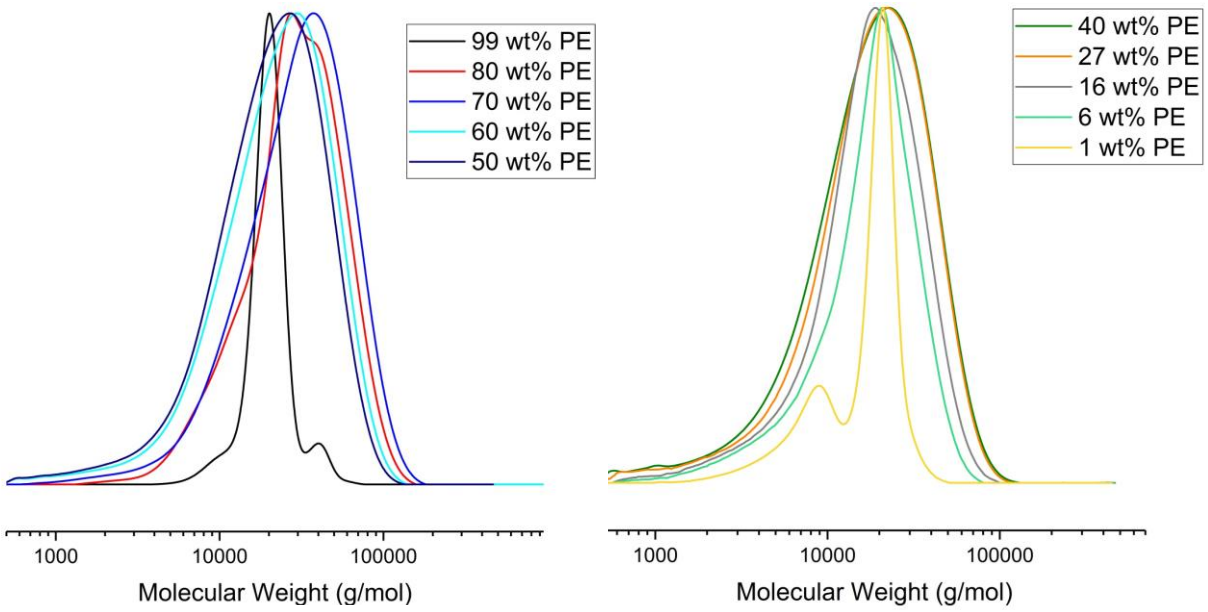

Supplementary Figure 40 – GPC traces of polymers based on TCA1/PO with different block ratios. Bimodality is attributed to 1) the presence of Cl initiating groups from the PPNCl co-catalyst; 2) feedstock impurities which become more evident at higher monomer loadings.

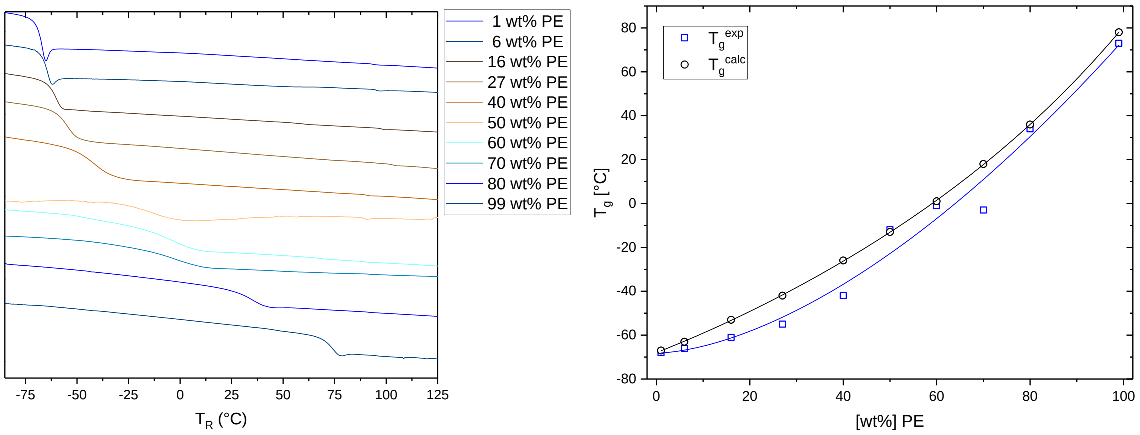

Supplementary Figure 41 – Thermal analysis of synthesised block copolymers. The recorded DSC thermograms are shown on the right and the obtained T_g values are compared with theoretical values (Fox equation for miscible polymers) on the right.

Supplementary Table 10 – TCA1/PO switch catalysis with varying monomer:catalyst loading.

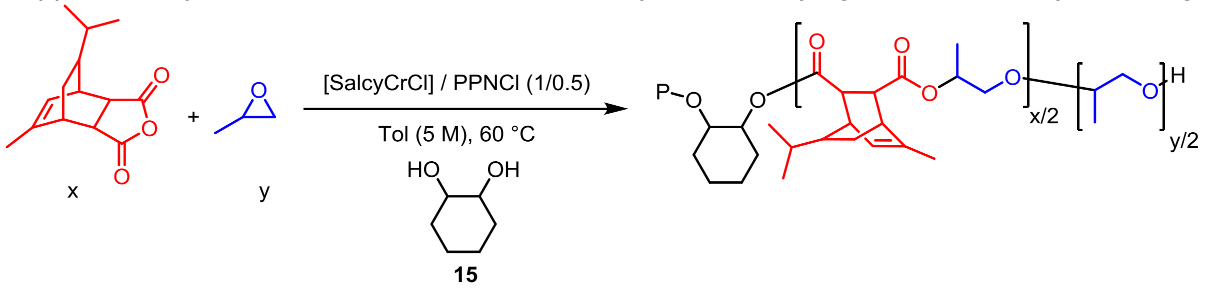

TCA1:PO[1]	Rct-time	Conversion[%][2] PO | TCA1	PE:PPO [wt%][3]	M_n ($Đ$) [g mol^{-1}][4]	T_g [°C][5]
100:200	2 d	95 | 99	51:49	3,400 (1.12)	N. D.#
200:400	5 d	90 | 99	53:47	6,700 (1.12)	41 °C
300:600	5 d	> 95 | 99	83:17	7,600 (1.13)	34 °C
400:800	5 d	> 95 | 99	83:17	9,900 (1.11)	37 °C
500:1000	5 d	94 | 99	83:17	26,200 (1.05)<br>11,800 (1.10)	34 °C
600:1200	10 d	> 95 | 99	83:17	38,500 (1.06)<br>13,700 (1.23)	21 °C
700:1400	10 d	> 95 | 99	83:17	38,900 (1.06)<br>14,600 (1.21)	24 °C
800:1600	10 d	> 95 | 99	83:17	38,500 (1.07)<br>14,400 (1.21)	35 °C
900:1800	10 d	> 95 | 99	83:17	21,500 (1.61)	19 °C
1000:2000	10 d	> 95 | 99	83:17	54,200 (1.09)<br>18,200 (1.25)	N. D.

[1]Molar ratio with respect to catalyst. [2]Based on ^{1}H NMR spectroscopy. The following chemical shifts were used: 1.04-1.00 ppm (TCA1), 3.72-3.25 ppm (PPO; the conversion was calculated based on the theoretical maximum conversion of PO). [3]Based on ^{1}H NMR spectroscopy of crude sample. PE content was calculated based on shifts for PE (5.85-5.55, HC=C) and PPO (3.72-3.25 ppm), i. e. Integral PE/(Integral PE+Integral PPO/3). [4]Measured by GPC (30°C, THF) with narrow polystyrene standards. [5]Measured by DSC, 3rd heating cycle.

Soluble in Pentane (not isolated).

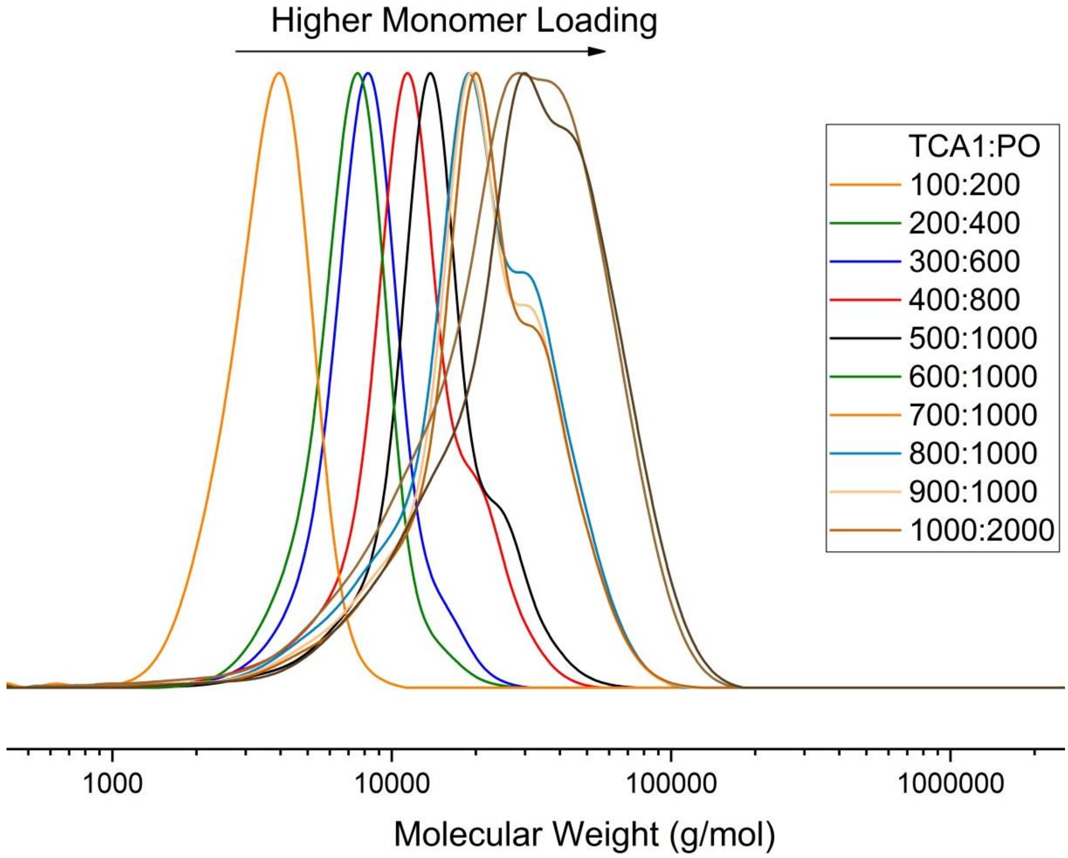

Supplementary Figure 42 – GPC traces for polymers with different monomer loadings. Bimodality is attributed to 1) the presence of Cl initiating groups from the PPNCl co-catalyst; 2) feedstock impurities which become more evident at higher monomer loadings times.

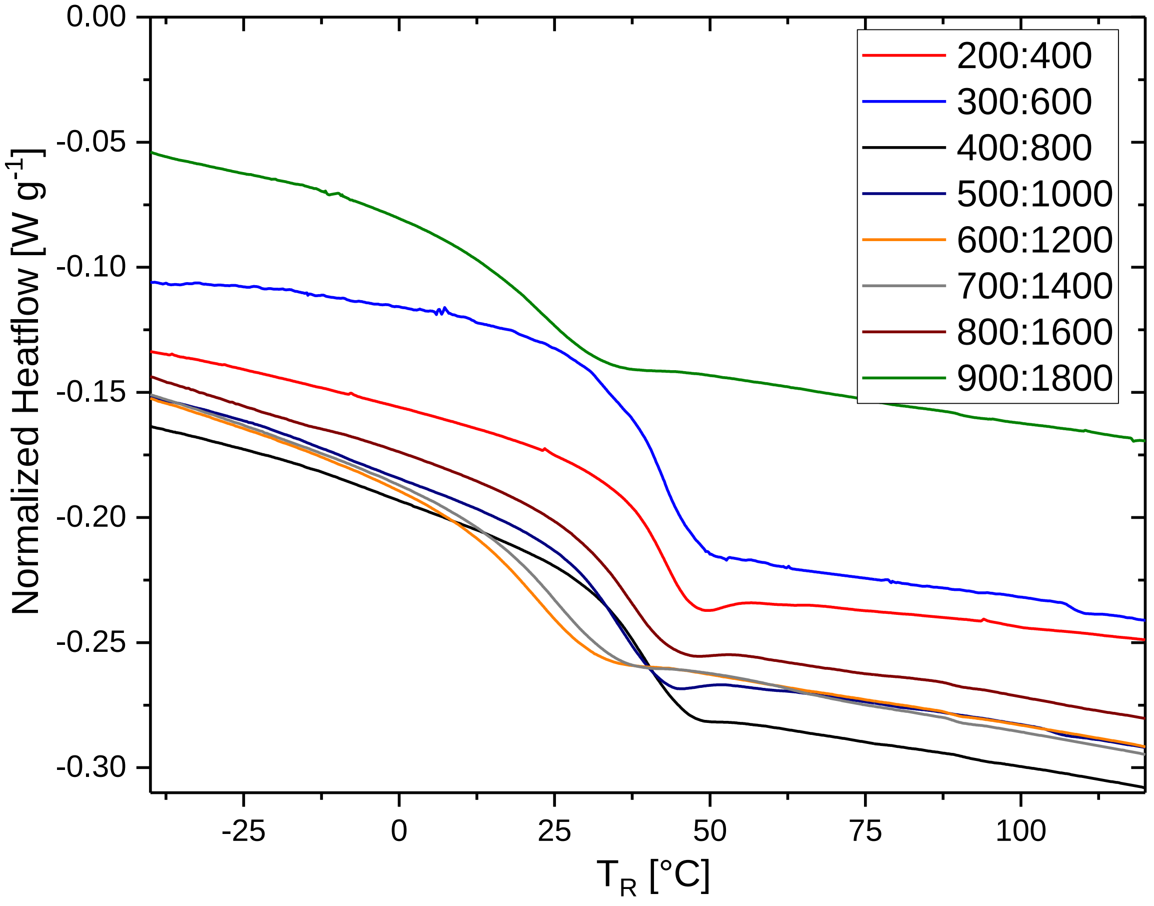

Supplementary Figure 43 – DSC thermograms of polymer obtained from TCA1/PO with different monomer:catalyst loadings.

Supplementary Table 11 – Epoxide scope with higher monomer loading for selected epoxides.

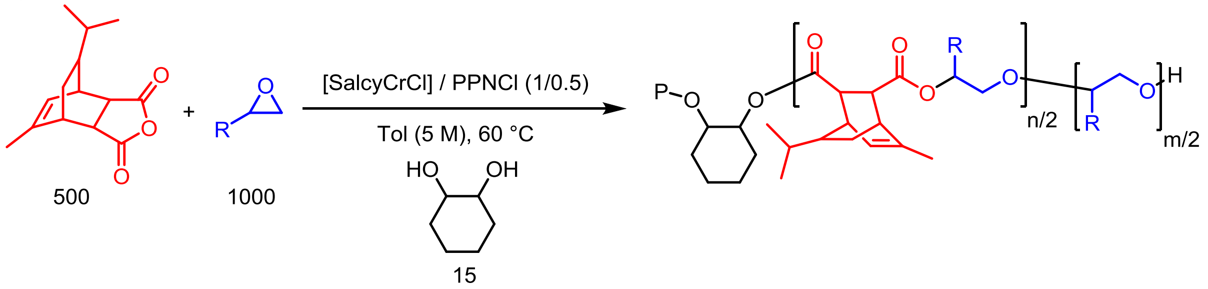

Epoxide	Conversion (Epoxide relative to Homopolymer)	M_n ($Đ$)
tert-Butyl glycidyl ether	4 d: 31 % | 99 %<br>10 d: 69 % | 99 %<br>15 d: 91 % | 99 %	4d: 11,400 (1.10)<br>15d: 14,300 (1.21)
Styrene oxide	4 d: <5 % | 99 %<br>10 d: 52 % | 99 %<br>15 d: 57 % | 99 %	4d: 8,500 (1.11)<br>15d: 8,300 (1.16)
2-Vinyloxirane	4 d: 64 % | 99 %<br>15 d: >95 % | 99 %	4d: 9,700 (1.10)<br>15d: 9,800 (1.10)
Allyl glycidyl ether	4 d: 80 % | 99 %<br>15 d: >95 % | 99 %	4d: 31,600 (1.03) + 13,700 (1.13)<br>15d: 14,900 (1.24)
2-Ethylhexyl glycidyl ether	4 d: 31 % | 99 %<br>15 d: >95 % | 99 %	4d: 13,900 (1.13)<br>15d: 15,900 (1.20)

§ An insoluble material, most likely due to cross-linking, was obtained after isolation.

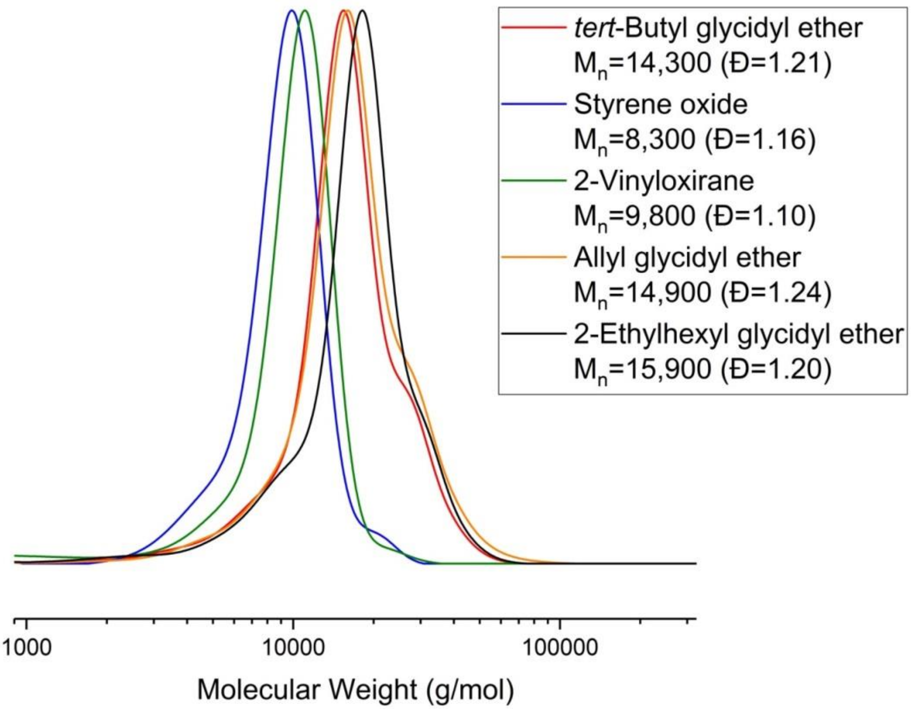

Supplementary Figure 44 – GPC traces of one-pot reactions of different epoxides with TCA1.

Supplementary Table 12 – One-pot polymerization of TCA1/PO with various amounts of *iso*-propanol as CTA.

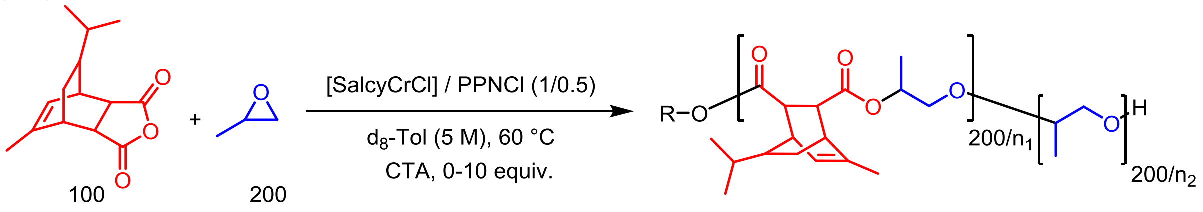

CTA (equiv.)	Rct-time	Conversion[%][2] PO | TCA1	M_n ($Đ$) [g mol^{-1}][4]	T_g [°C][5]
0	2d	89 % | 99 %	54,900 (1.04)<br>26,000 (1.04)	N. D.
0	2d	88 % | 99 %	48,200 (1.03)<br>23,200 (1.04)	43 °C
2	2d	78 % | 99 %	28,700 (1.02)<br>13,300 (1.04)	45 °C
4	2d	85 % | 99 %	20,100 (1.01)<br>8,800 (1.05)	42 °C
6	2d	74 % | 99 %	5,100 (1.08)	38 °C
8	2d	79 % | 99 %	4,500 (1.08)	29 °C
10	2d	90 % | 99 %	3,500 (1.09)	N. D.#

#Soluble in pentane, not isolated.

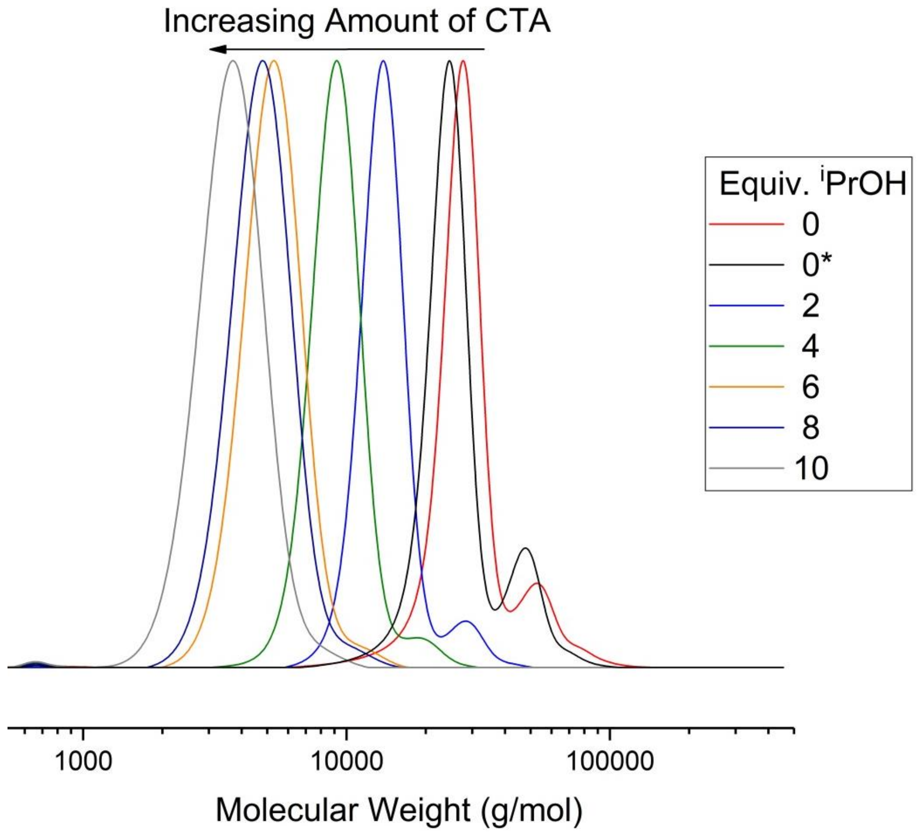

Supplementary Figure 45 – GPC traces of the one-pot polymerization of TCA1/PO with various amounts of CTA.

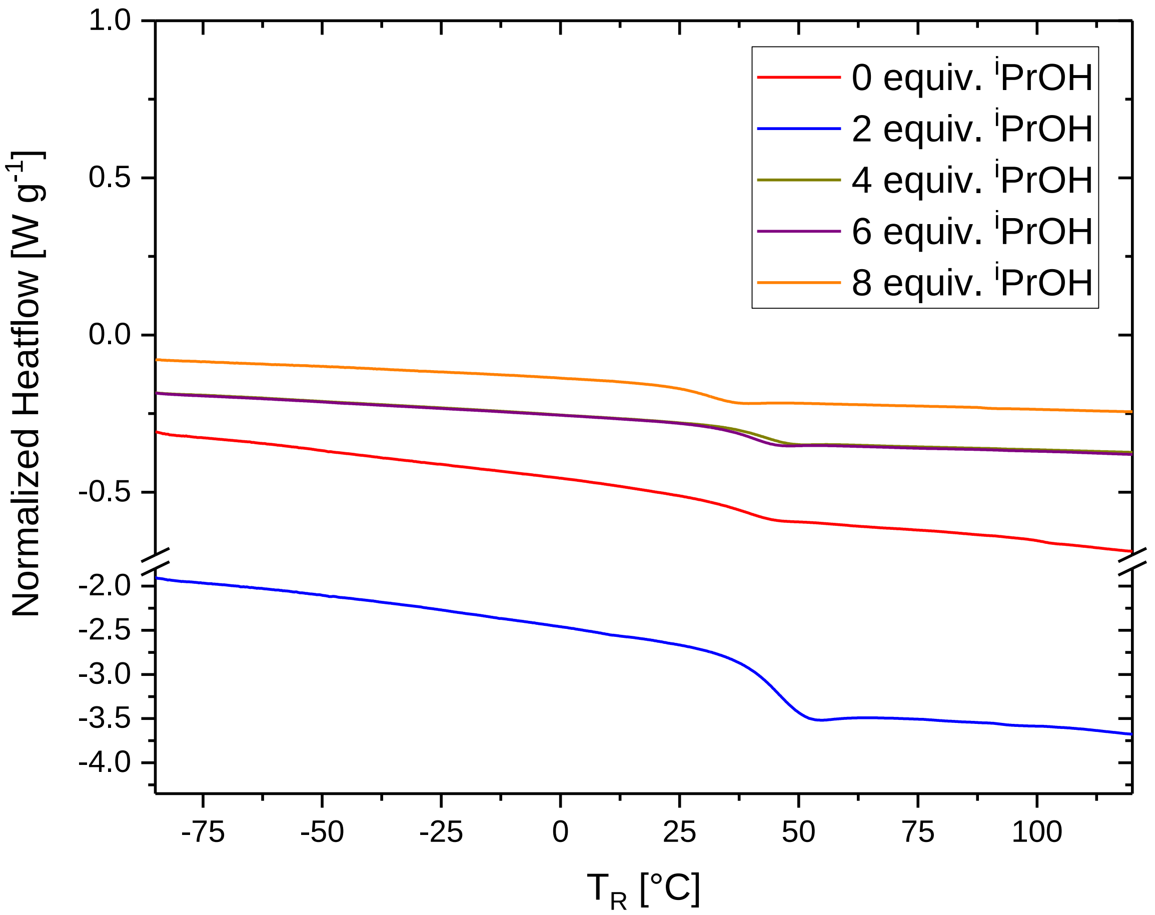

Supplementary Figure 46 – DSC thermograms of polymers based on TCA1/PO with varying amounts of ^{i}PrOH as CTA.

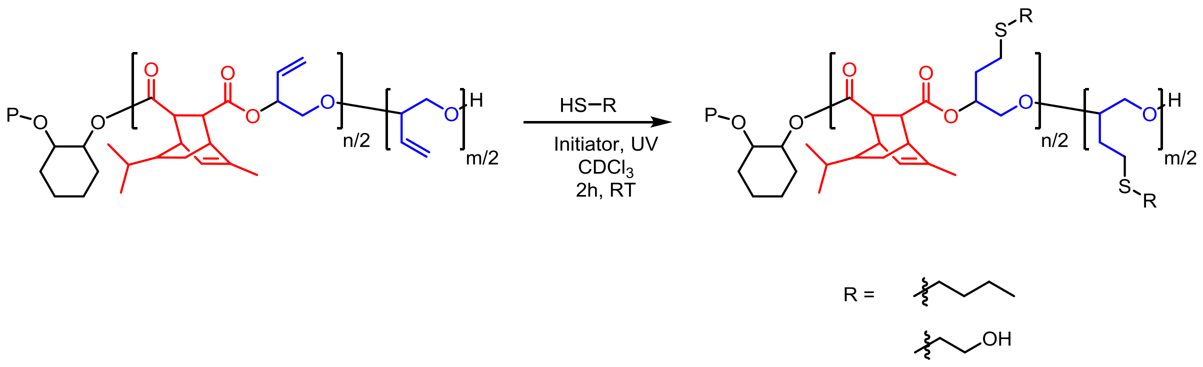

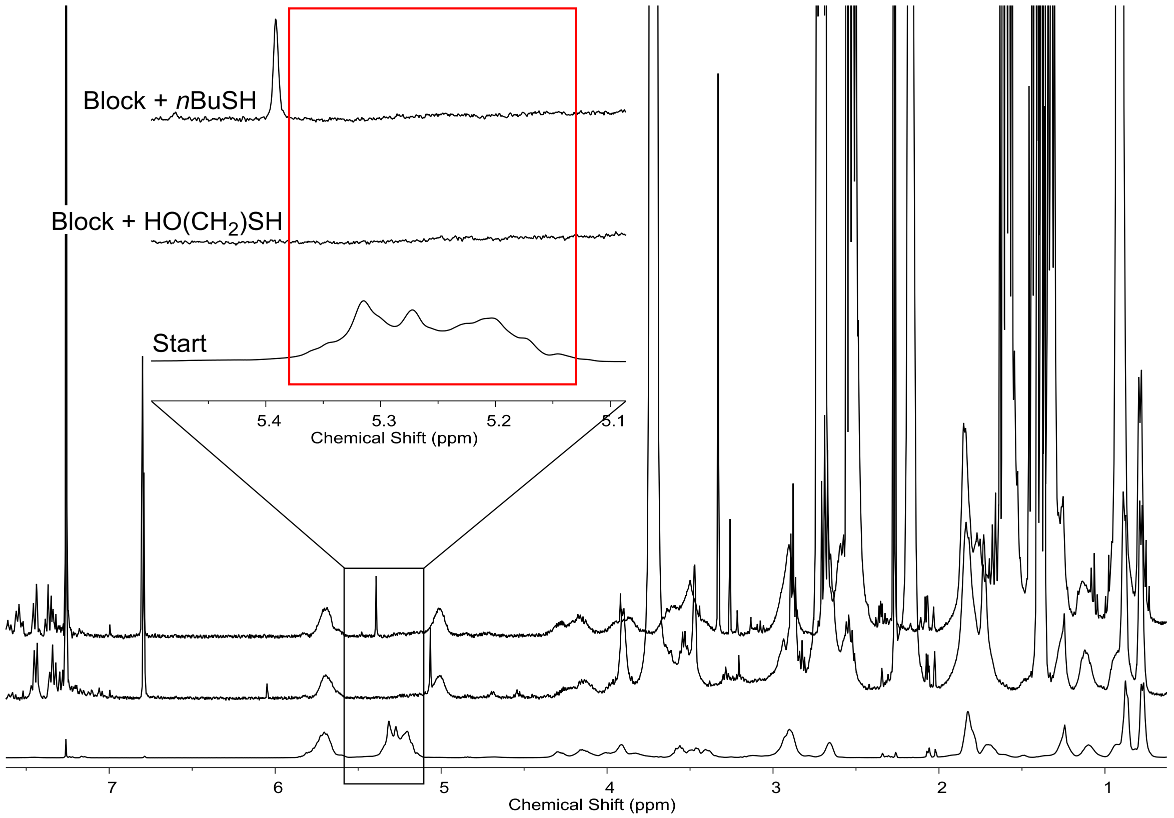

Supplementary Figure 47 – ^{1}H NMR Spectra of crude mixture after thiol-ene reactions. The relevant alkene region is enlarged (top left).

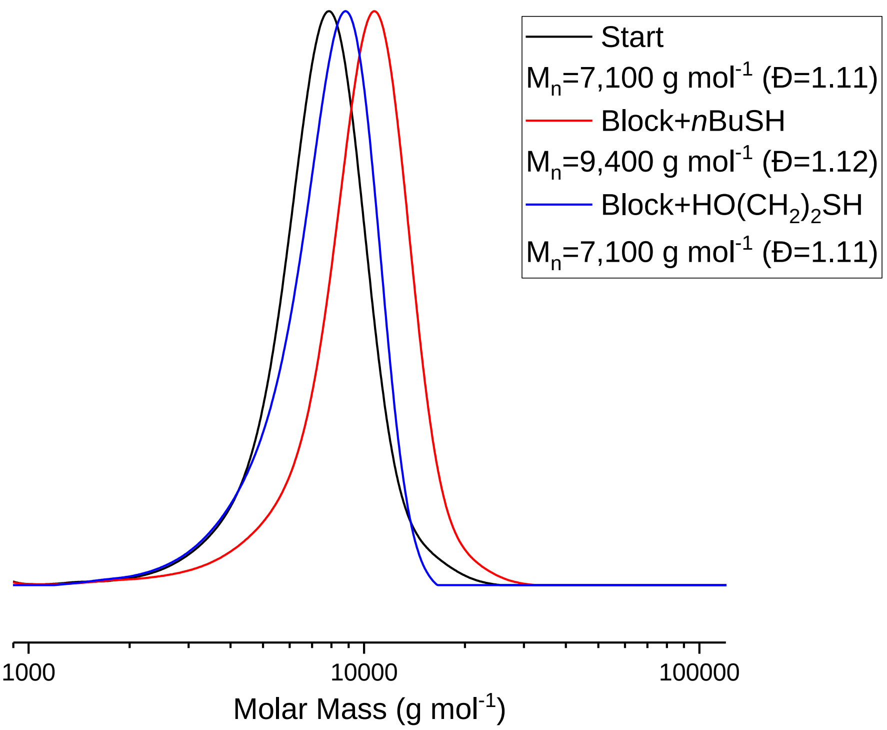

Supplementary Figure 48 – GPC traces before and after thiol-ene reactions.

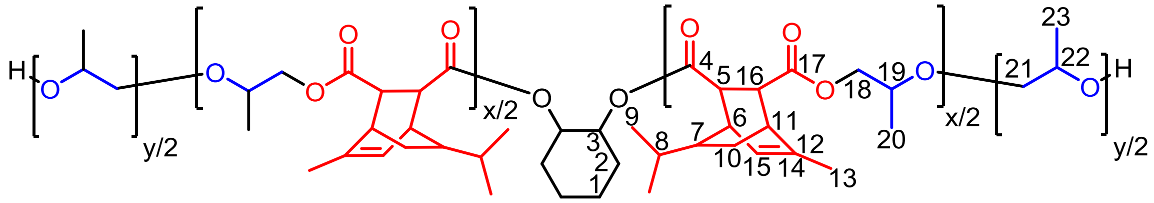

^{1}H NMR (400 MHz, $CDCl_3$) δ = 5.81-5.61 (H^{15}, m, 1.00 H), 5.21-4.84 (H^{19}, $H^{19\text{-Jct}}$, m, 0.94 H), 4.77-4.58 (H^3, m, 0.08 H), 4.31-3.71 (H^{18}, H18', $H^{23\text{-Endgrp}}$, m, 2.09 H), 3.67-3.27 (H^{21}, H^{22}, $H^{21\text{-Endgrp}}$, m, 2.23 H), 3.18-2.76 (H^5, H^{11}, H^{16}, m, 3.25 H), 2.72-2.55 (H^6, m, 0.90 H), 2.08-1.77 (H^{13}, m, 2.96 H), 1.76-1.53 (H^7, H^8, m, 1.66 H), 1.41-1.00 (H^1, H^2, H^{10}, H^{20}, H^{23}, $H^{20\text{-Jct}}$), 0.97-0.59 (H^9, m, 7.35 H).

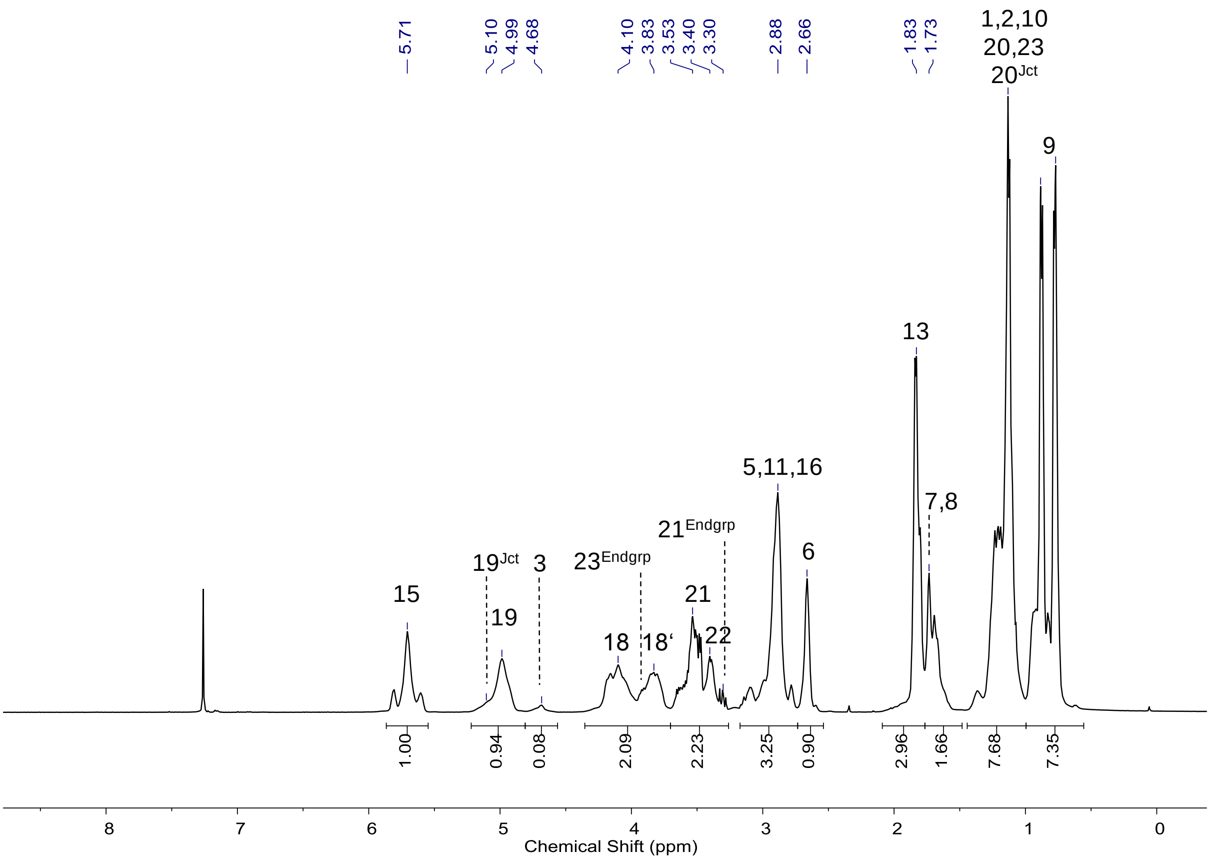

Supplementary Figure 49 – ^{1}H NMR Spectrum of block copolymer.

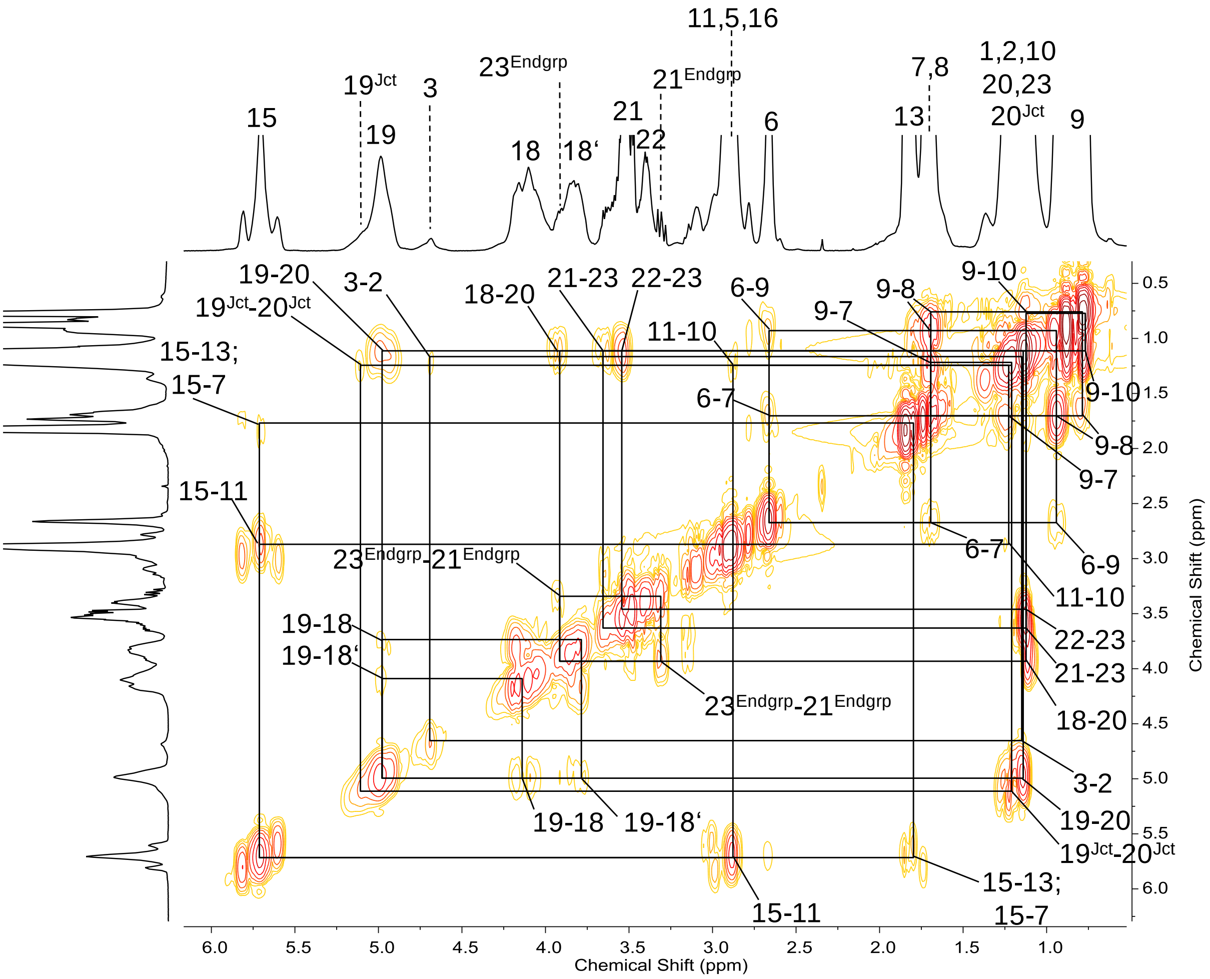

Supplementary Figure 50 – H, H COSY NMR Spectrum of block copolymer.

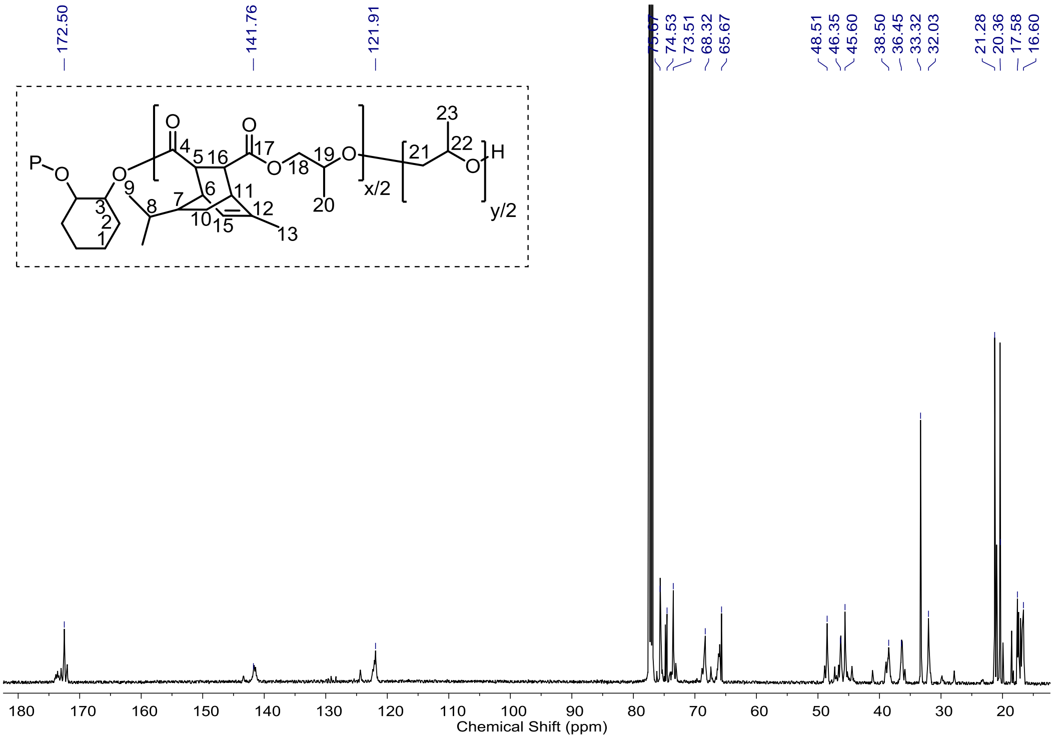

Supplementary Figure 51 – $^{13}C\{^{1}H\}$ NMR Spectrum of polymer obtained from TCA1/PO.

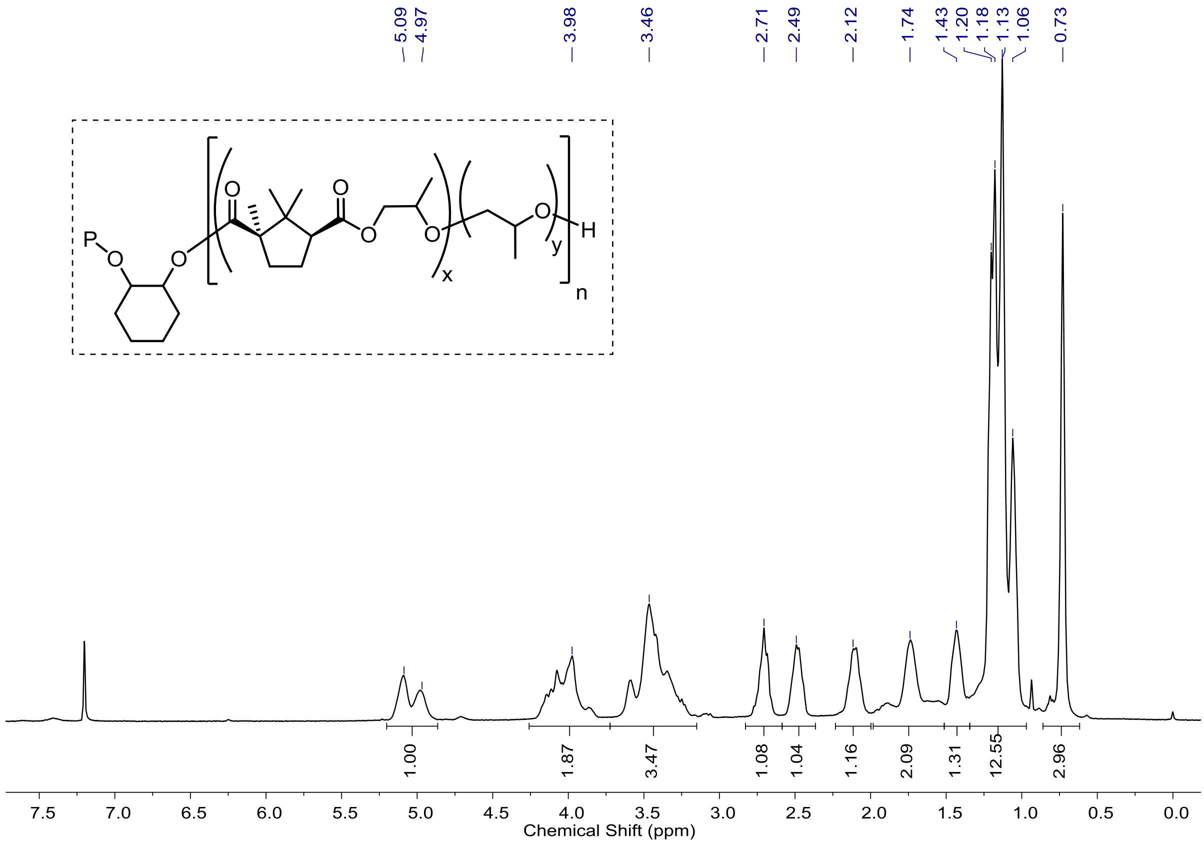

Supplementary Figure 52 – ^{1}H NMR Spectrum for polymer obtained from PO/CA.

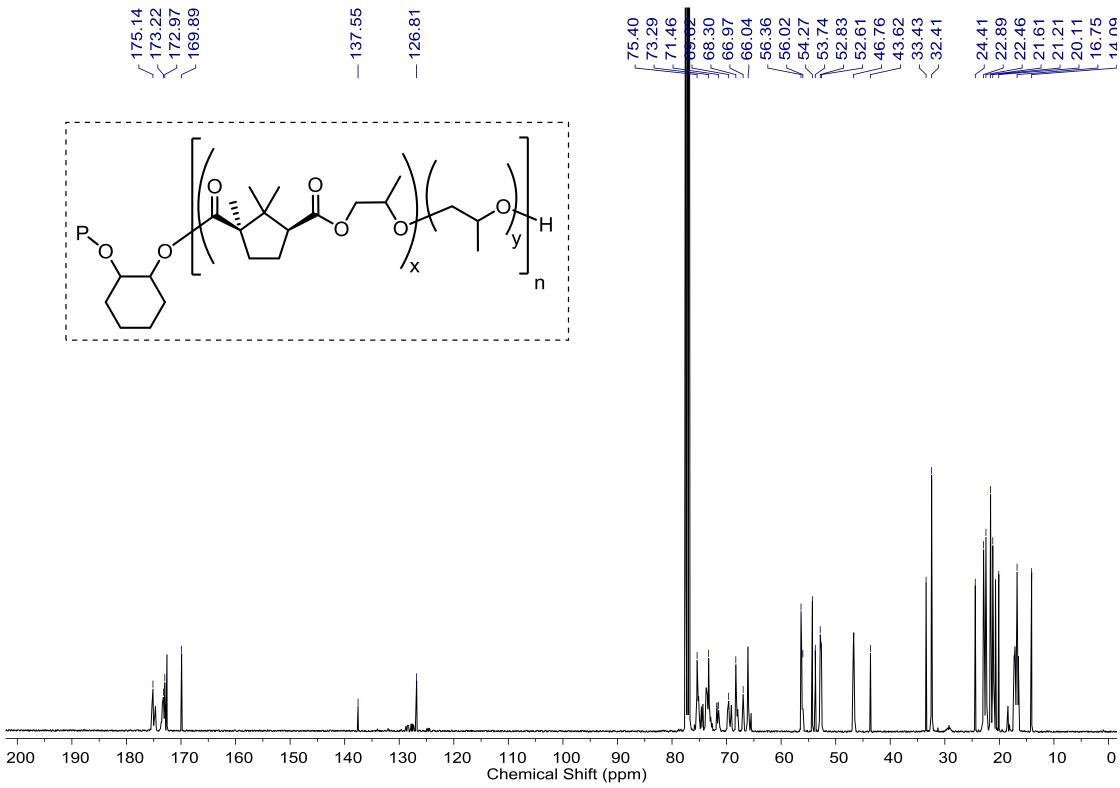

Supplementary Figure 53 – $^{13}C\{^{1}H\}$ NMR Spectrum for polymer obtained from PO/CA.

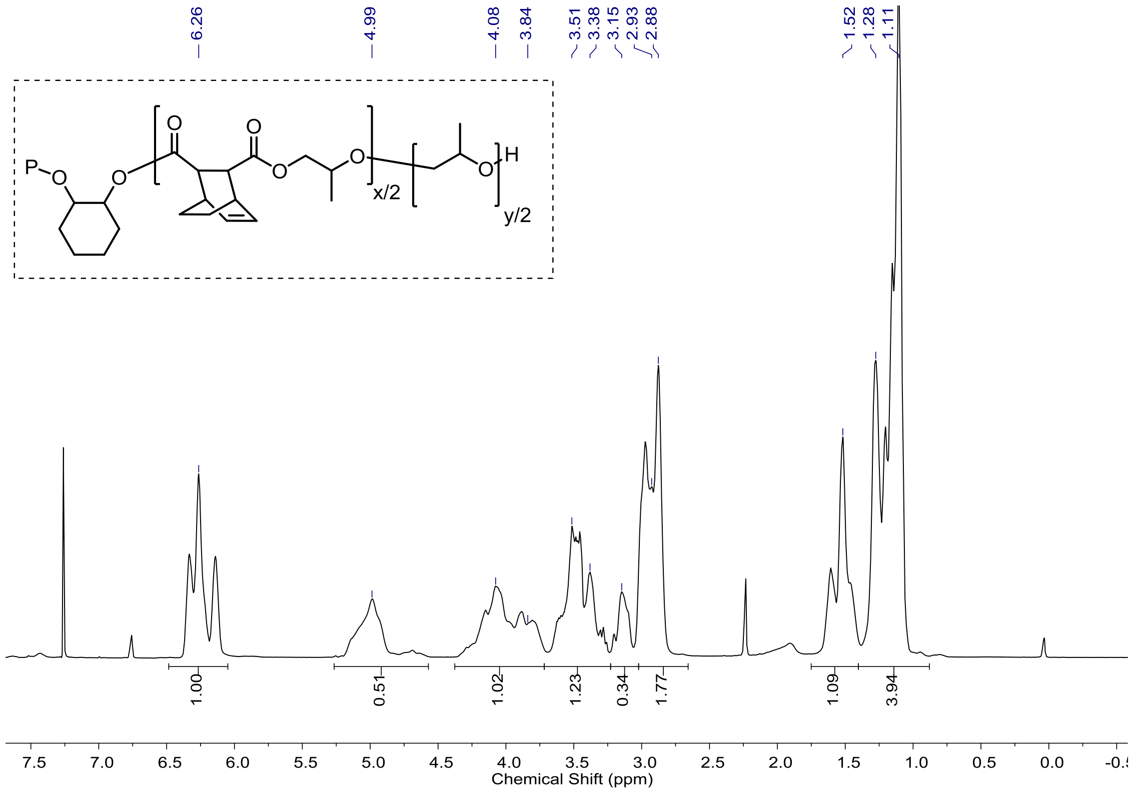

Supplementary Figure 54 – ^{1}H NMR Spectrum for polymer obtained from PO/TCA2. The signals at 6.78 and 2.26 ppm are attributed to residual amounts of mesitylene (< 3%).

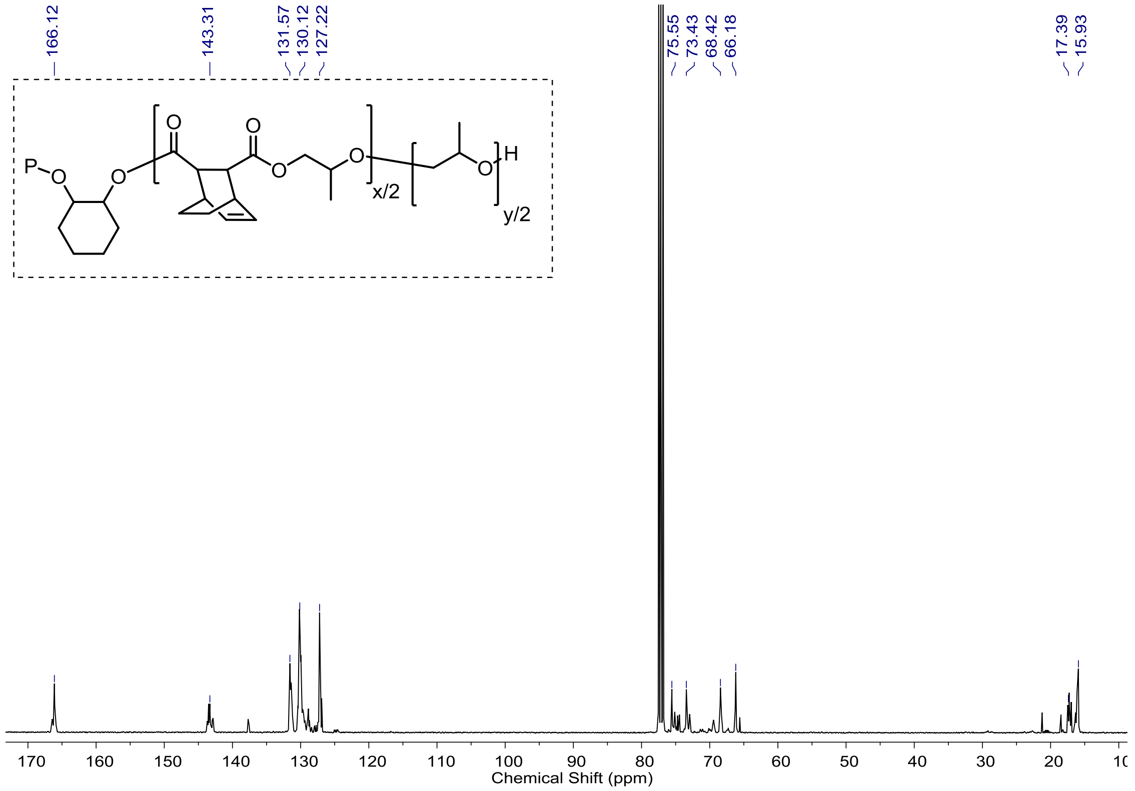

Supplementary Figure 55 – $^{13}C\{^{1}H\}$ NMR Spectrum for polymer obtained from PO/TCA2. The signals at 137.6 and 21 ppm are attributed to residual amounts of mesitylene (< 3%).

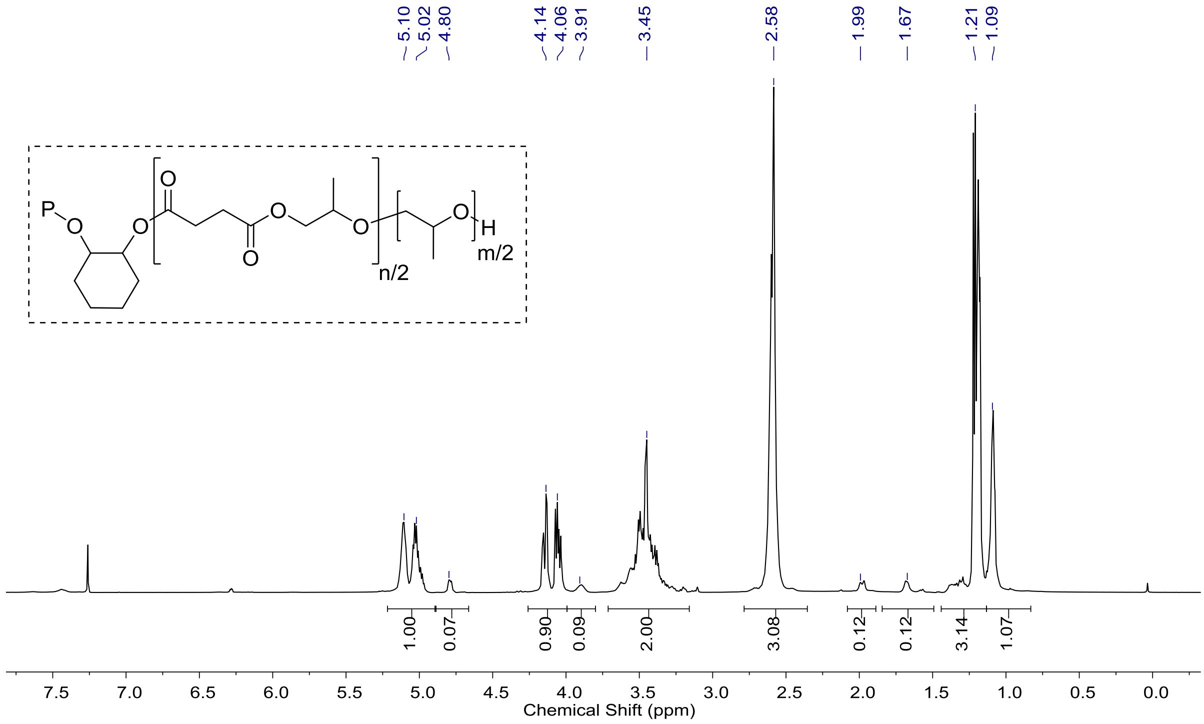

Supplementary Figure 56 – ^{1}H NMR Spectrum for polymer obtained from PO/SA.

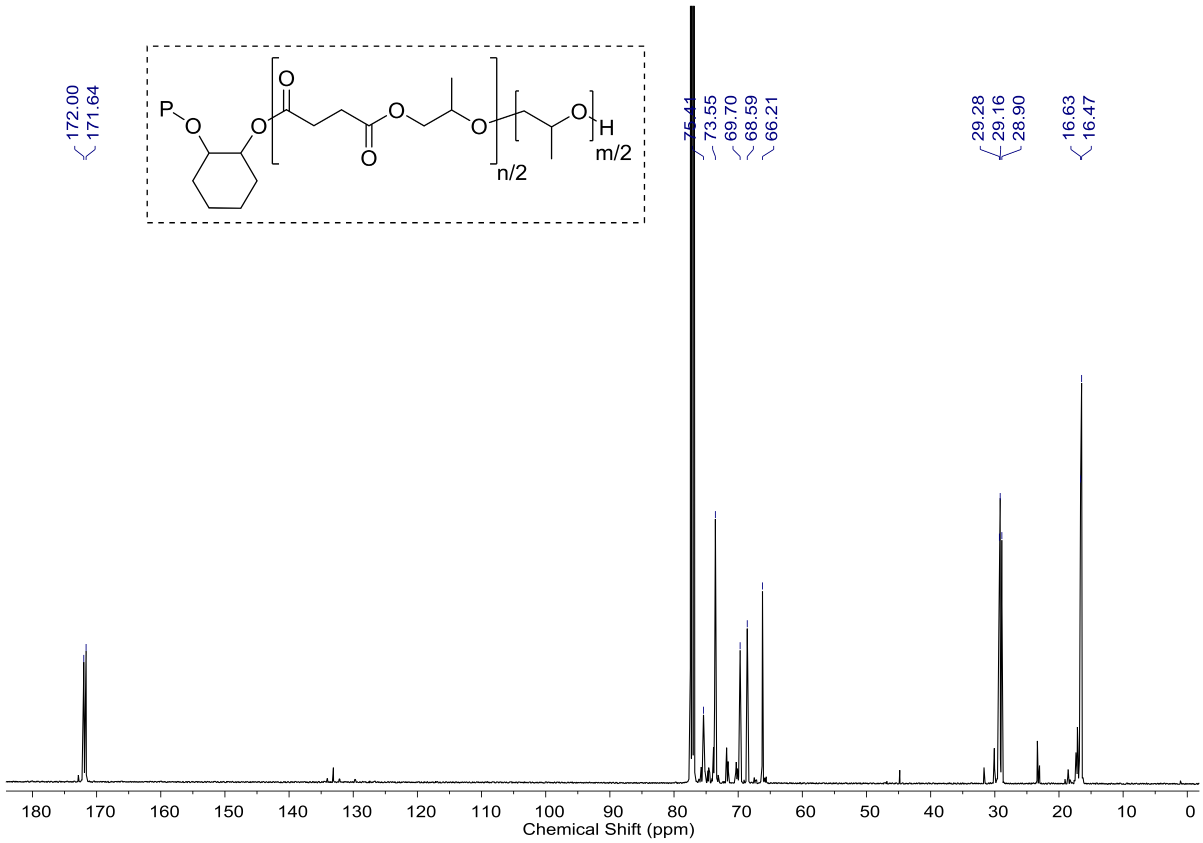

Supplementary Figure 57 – ^{13}C{^{1}H} NMR Spectrum for polymer obtained from PO/SA.

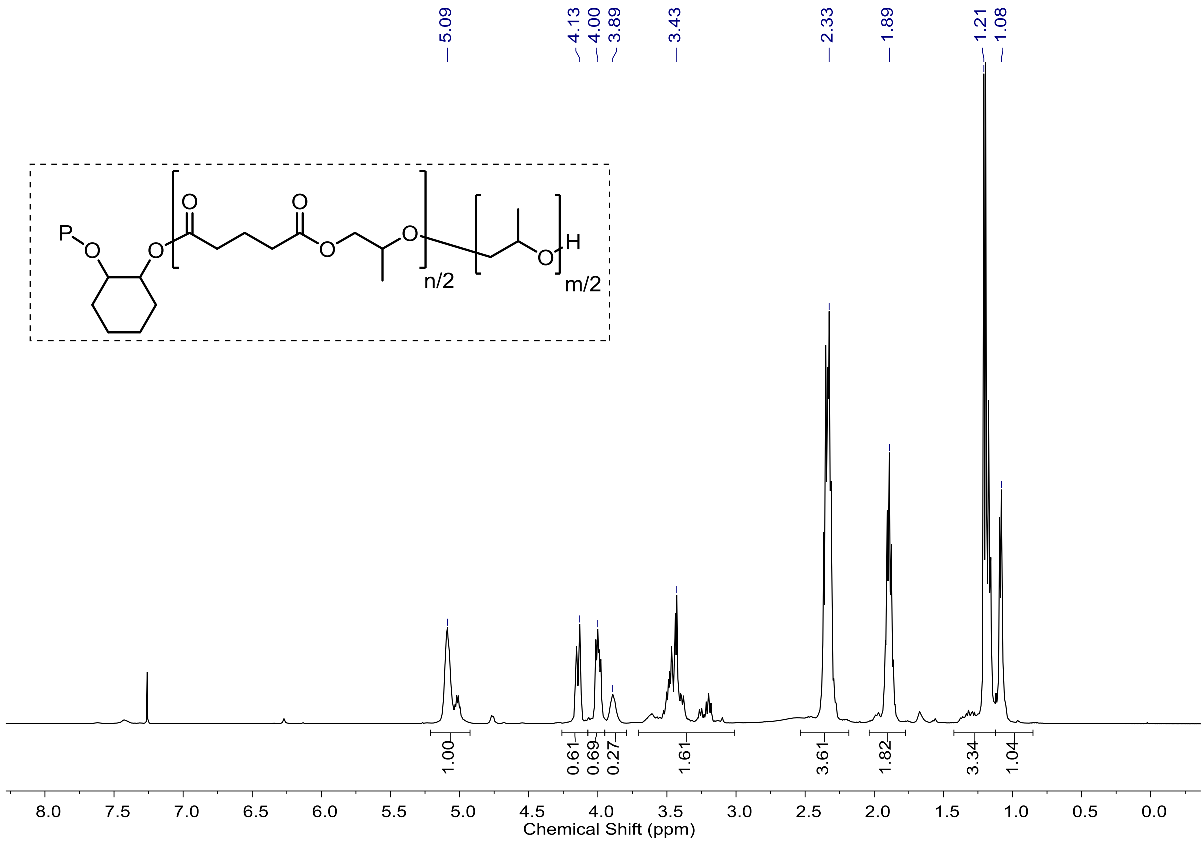

Supplementary Figure 58 – ^{1}H NMR Spectrum for polymer obtained from PO/GA.

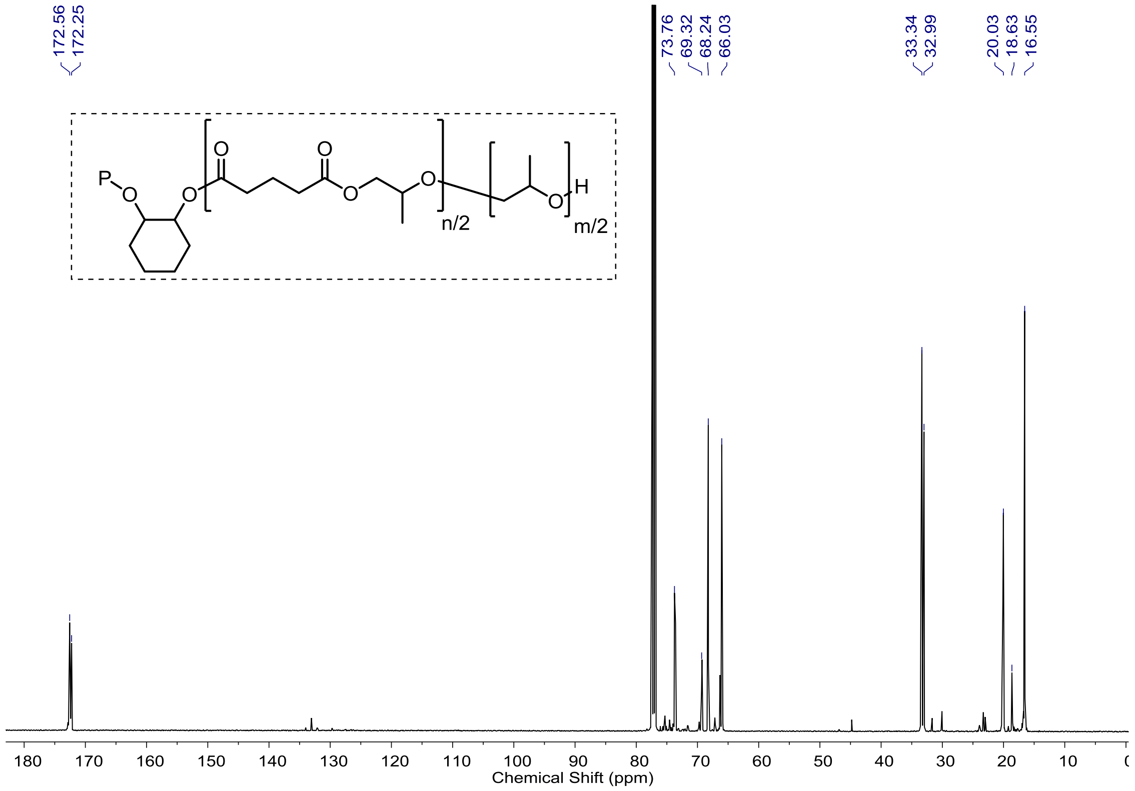

Supplementary Figure 59 – $^{13}C\{^{1}H\}$ NMR Spectrum for polymer obtained from PO/GA.

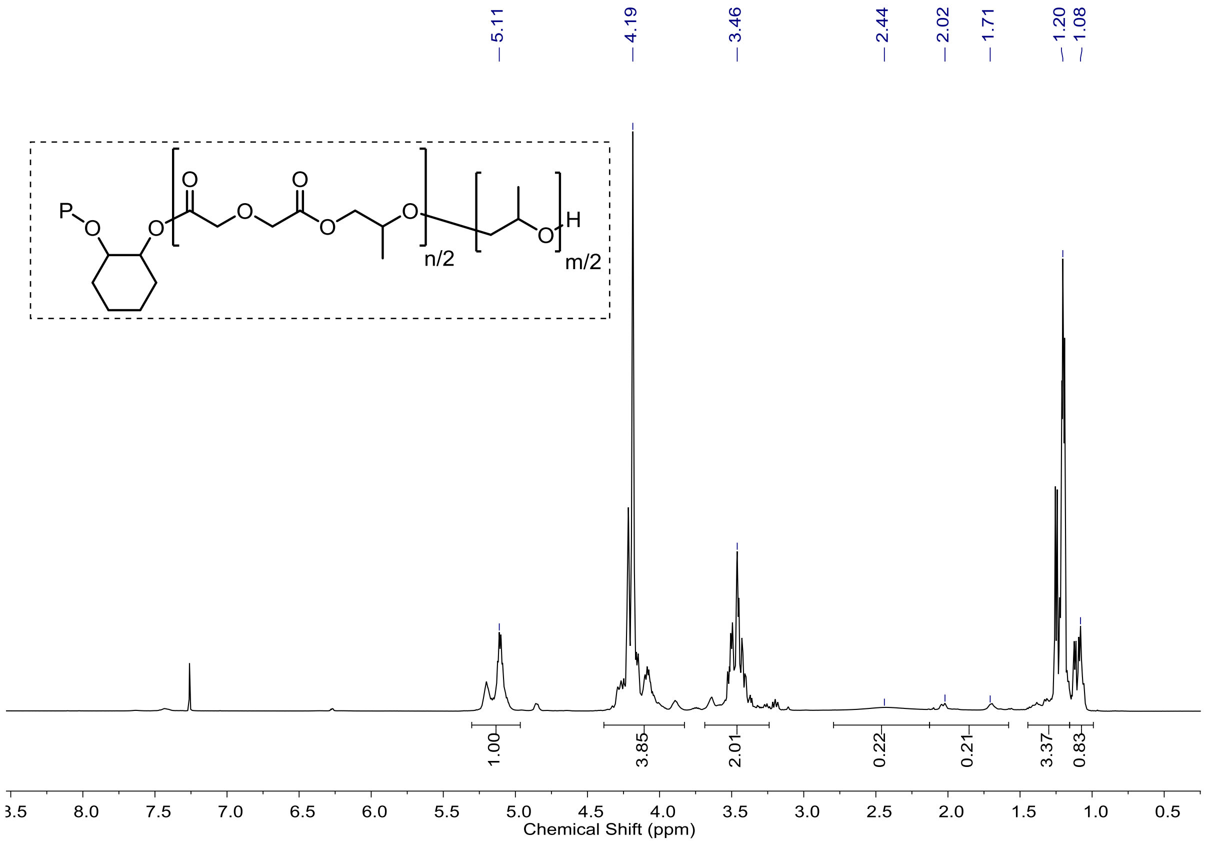

Supplementary Figure 60 – ^{1}H NMR Spectrum for polymer obtained from PO/DGA.

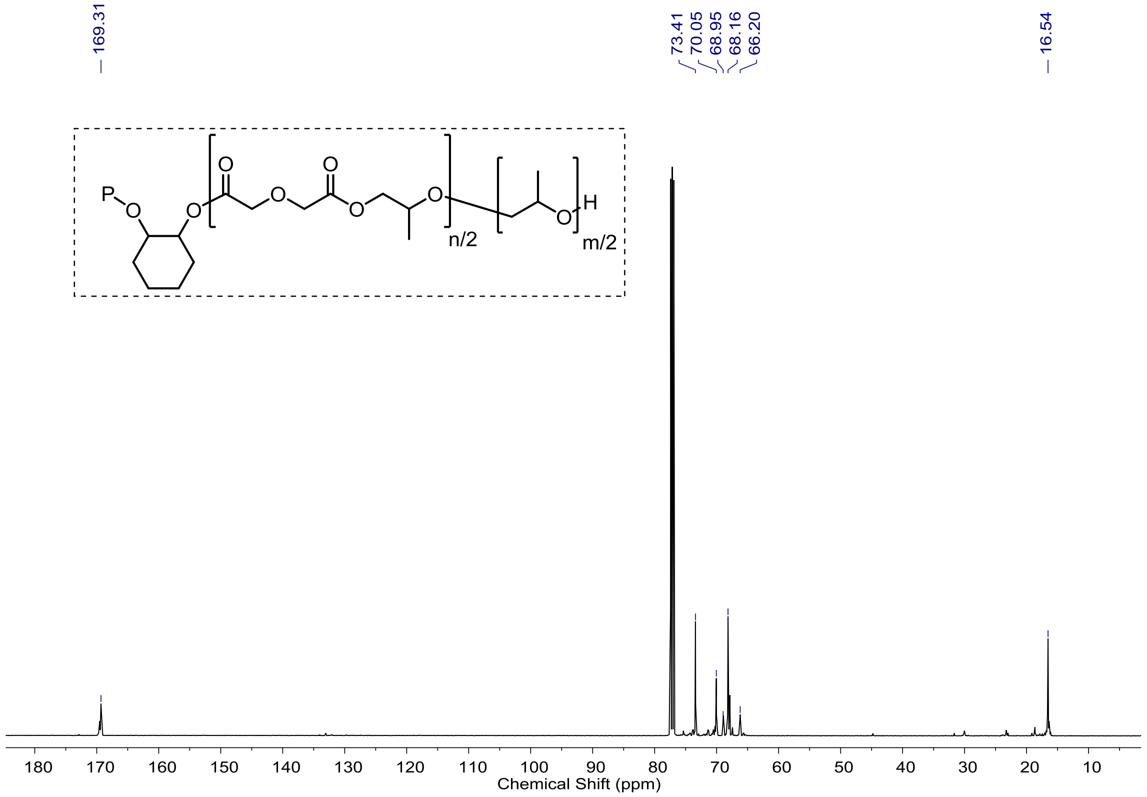

Supplementary Figure 61 – ^{13}C{^{1}H} NMR Spectrum for polymer obtained from PO/DGA.

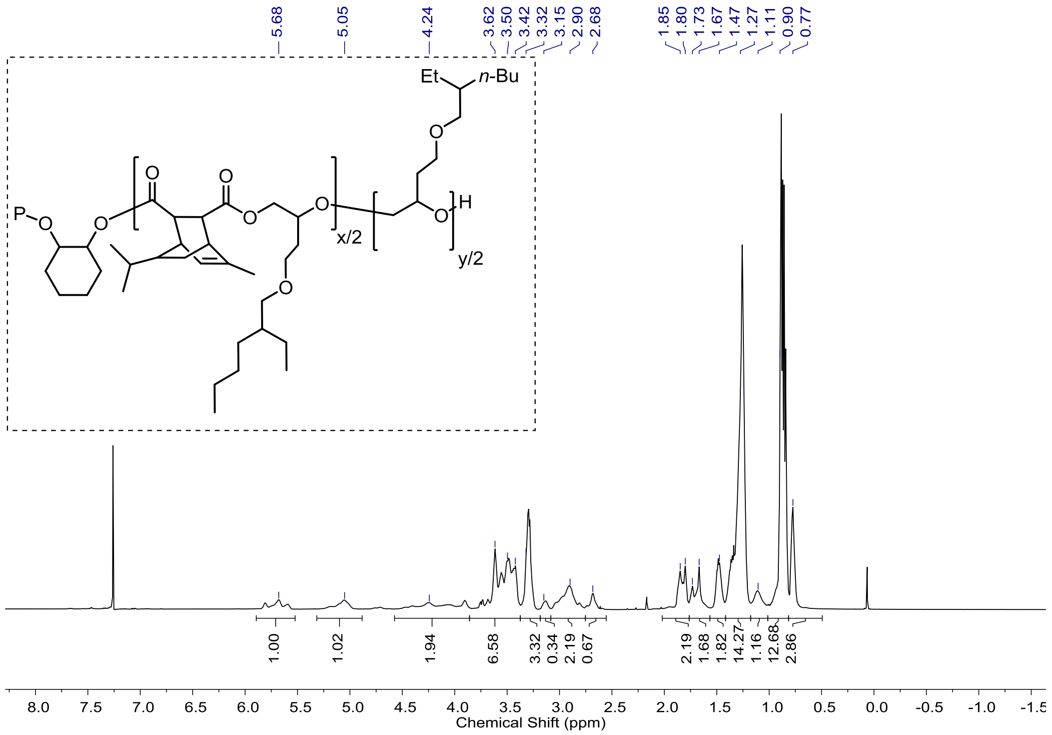

Supplementary Figure 62 – ^{1}H NMR Spectrum for polymer obtained from 2-EHGE/TCA1.

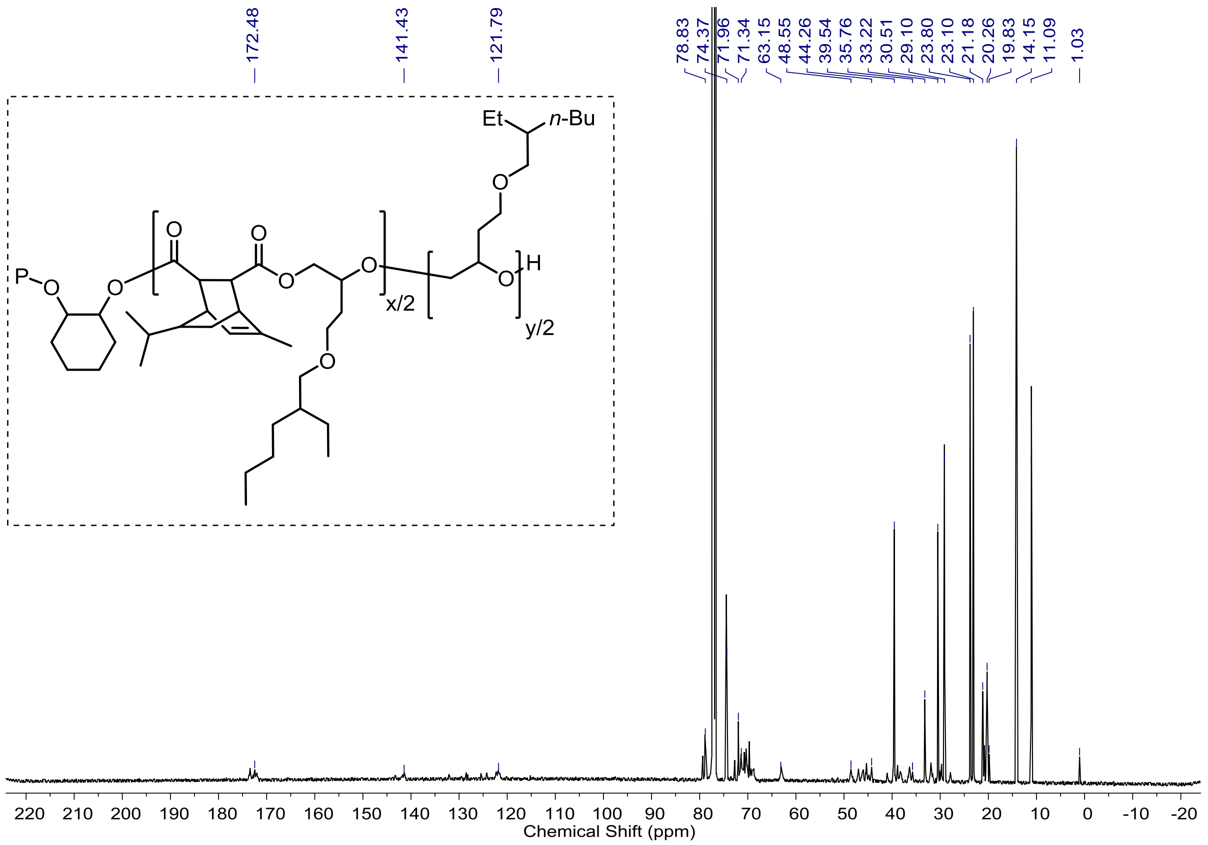

Supplementary Figure 63 – ^{13}C{^{1}H} NMR Spectrum for polymer obtained from 2-EHGE/TCA1.

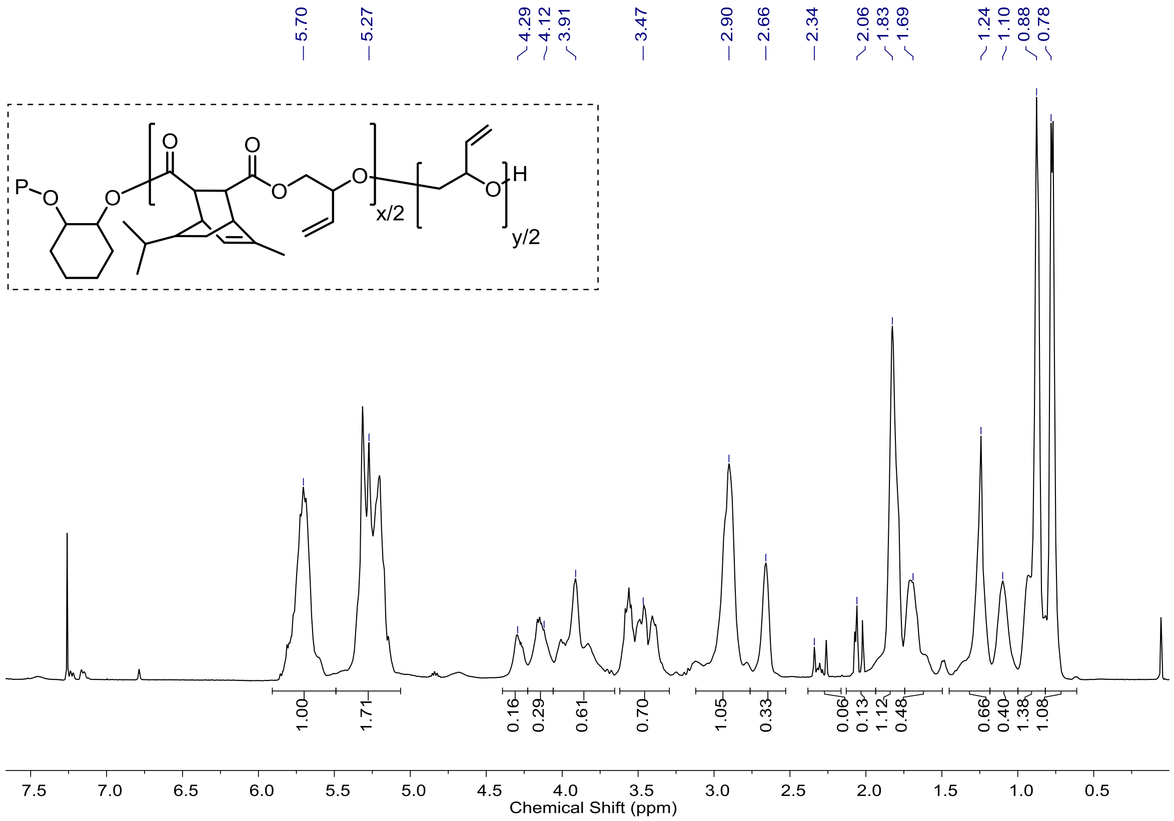

Supplementary Figure 64 – ^{1}H NMR Spectrum for polymer obtained from VPO /TCA1. The signals at 6.78 and 2.26 ppm are attributed to residual amounts of mesitylene (<1%).

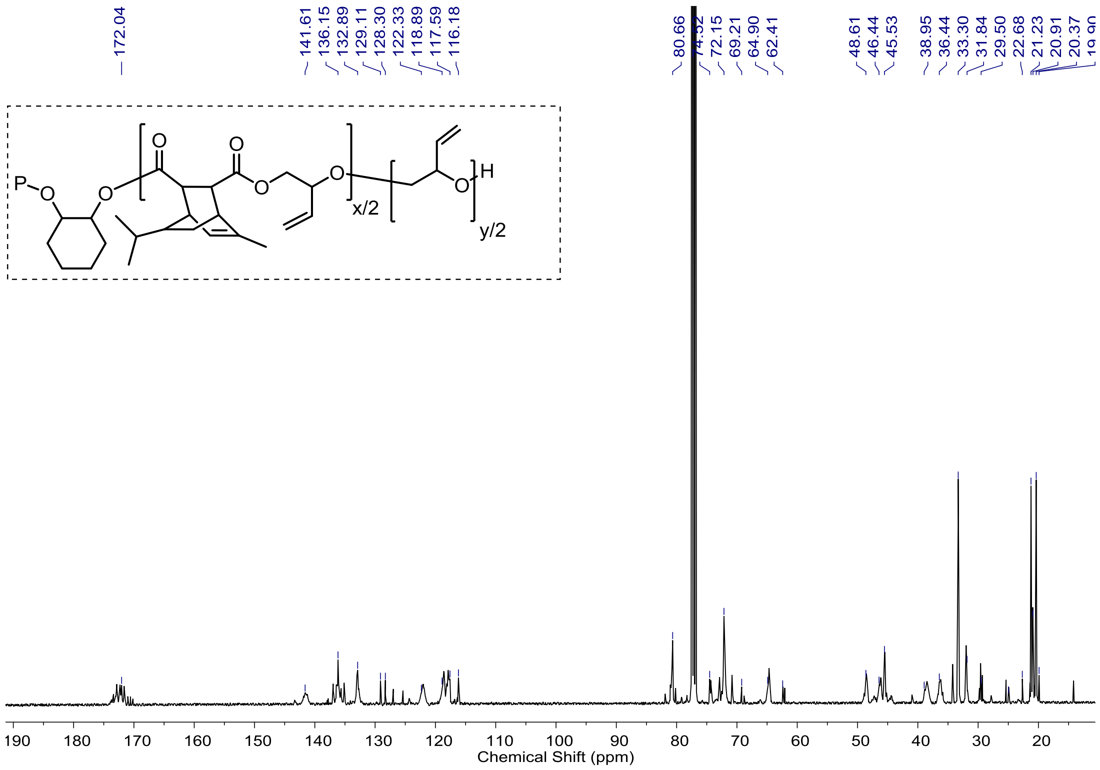

Supplementary Figure 65 – ^{13}C{^{1}H} NMR Spectrum for polymer obtained from VPO/TCA1.

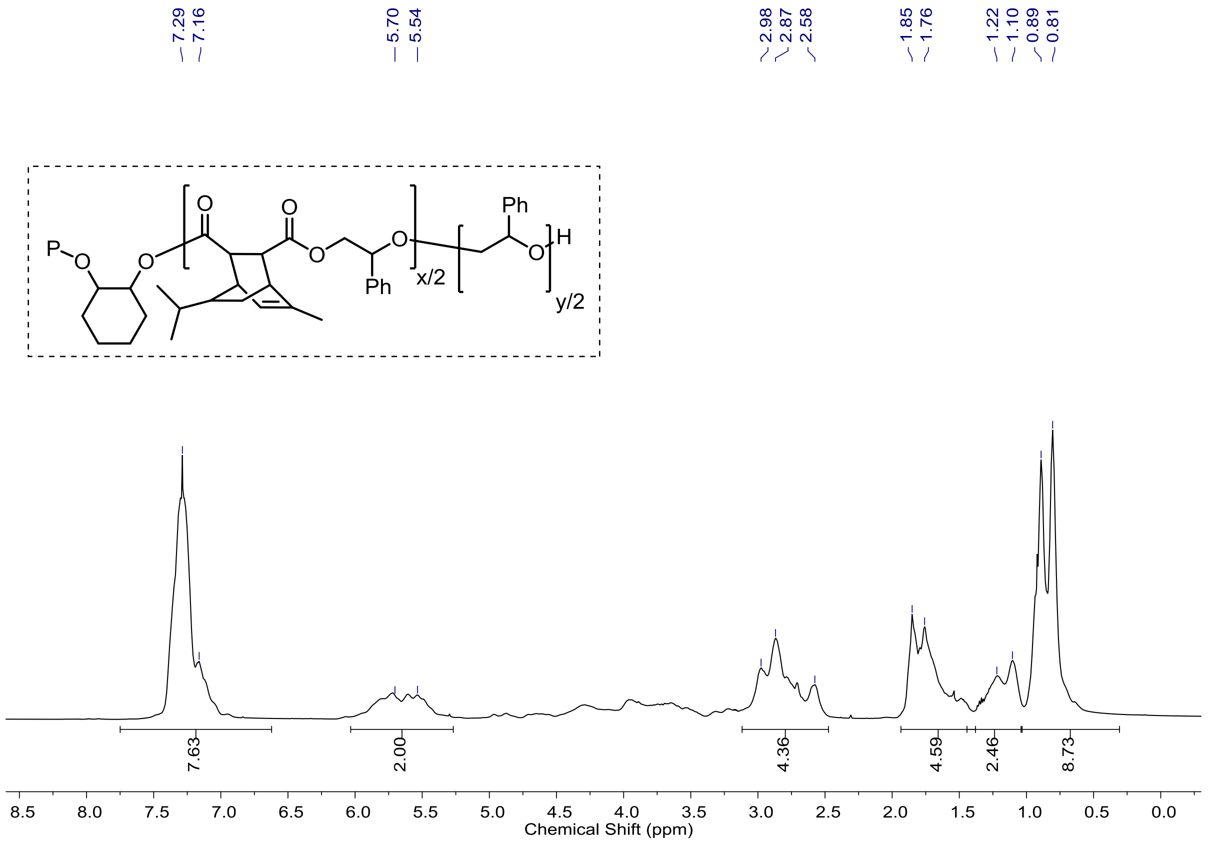

Supplementary Figure 66 – ^{1}H NMR Spectrum for polymer obtained from SO/TCA1.

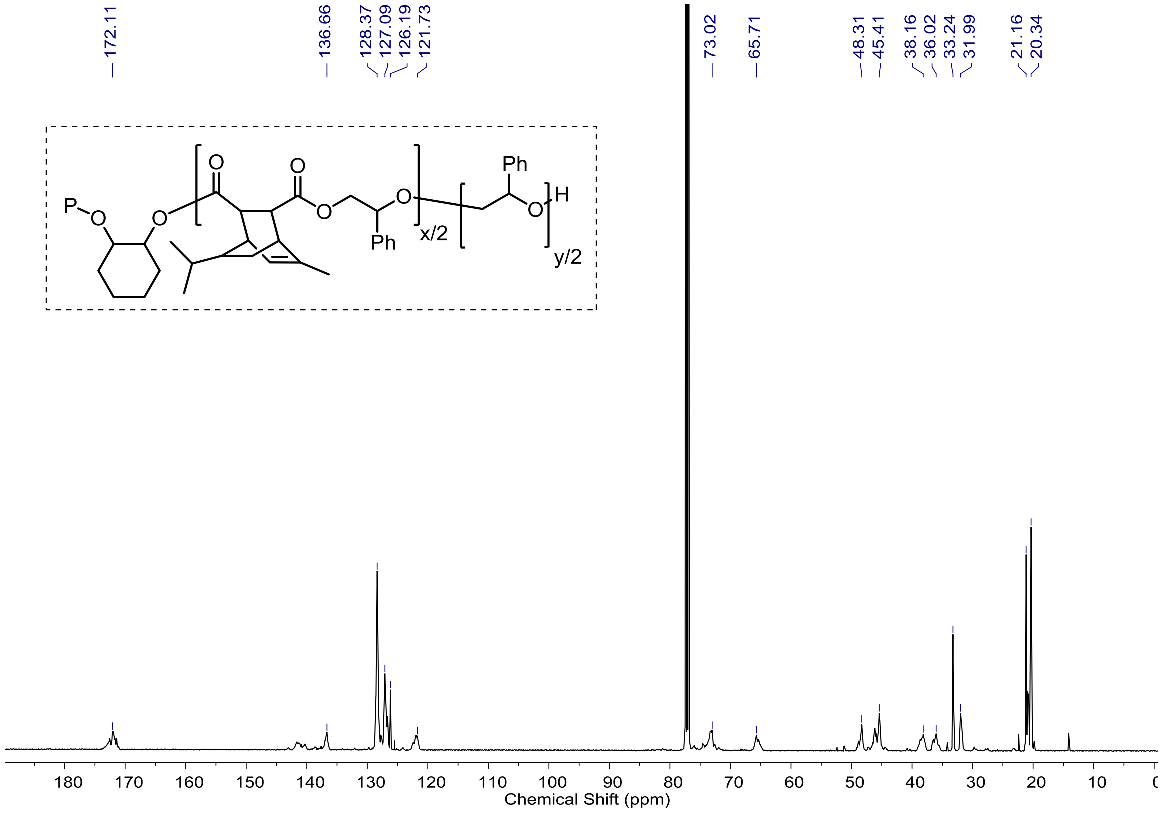

Supplementary Figure 67 – ^{13}C{^{1}H} NMR Spectrum for polymer obtained from SO/TCA1.

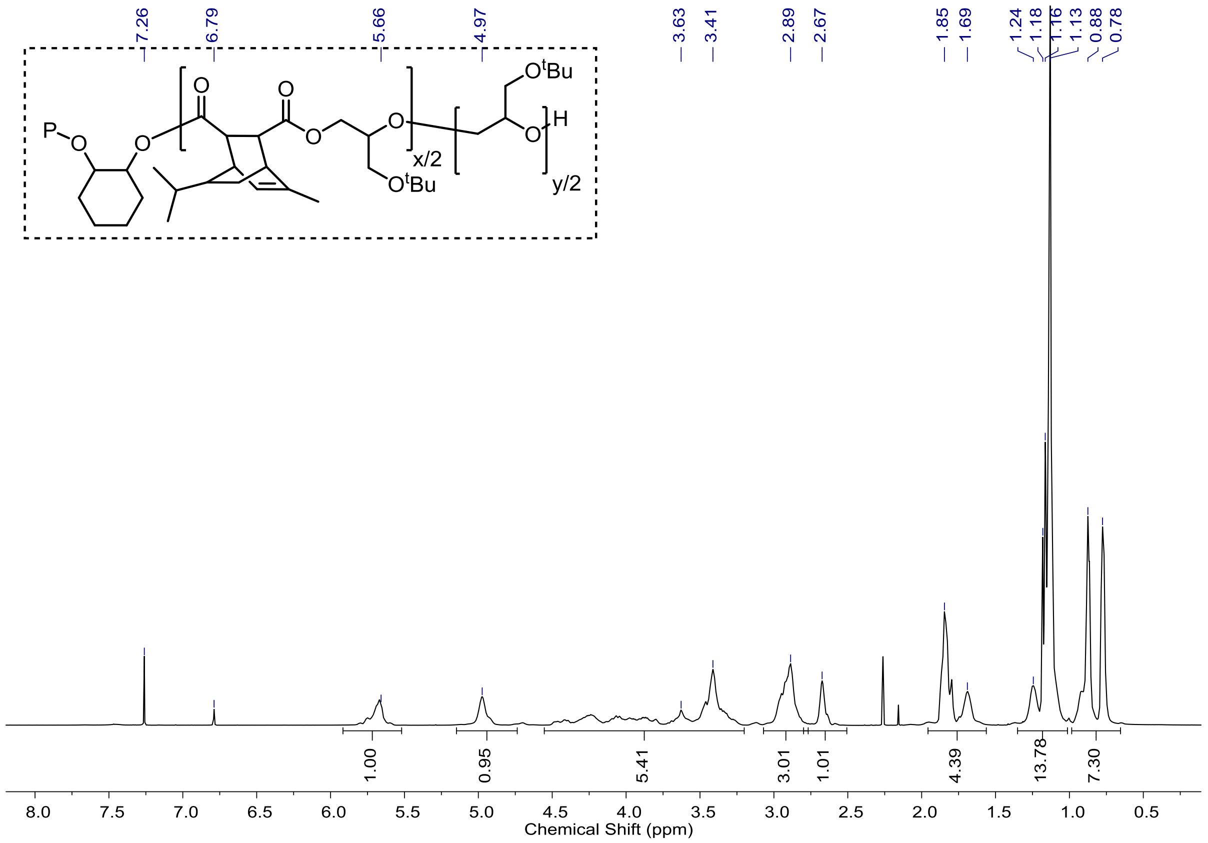

Supplementary Figure 68 – ^{1}H NMR Spectrum for polymer obtained from tBGE/TCA1. The signals at 6.78 and 2.26 ppm are attributed to residual amounts of mesitylene (ca. 1%).

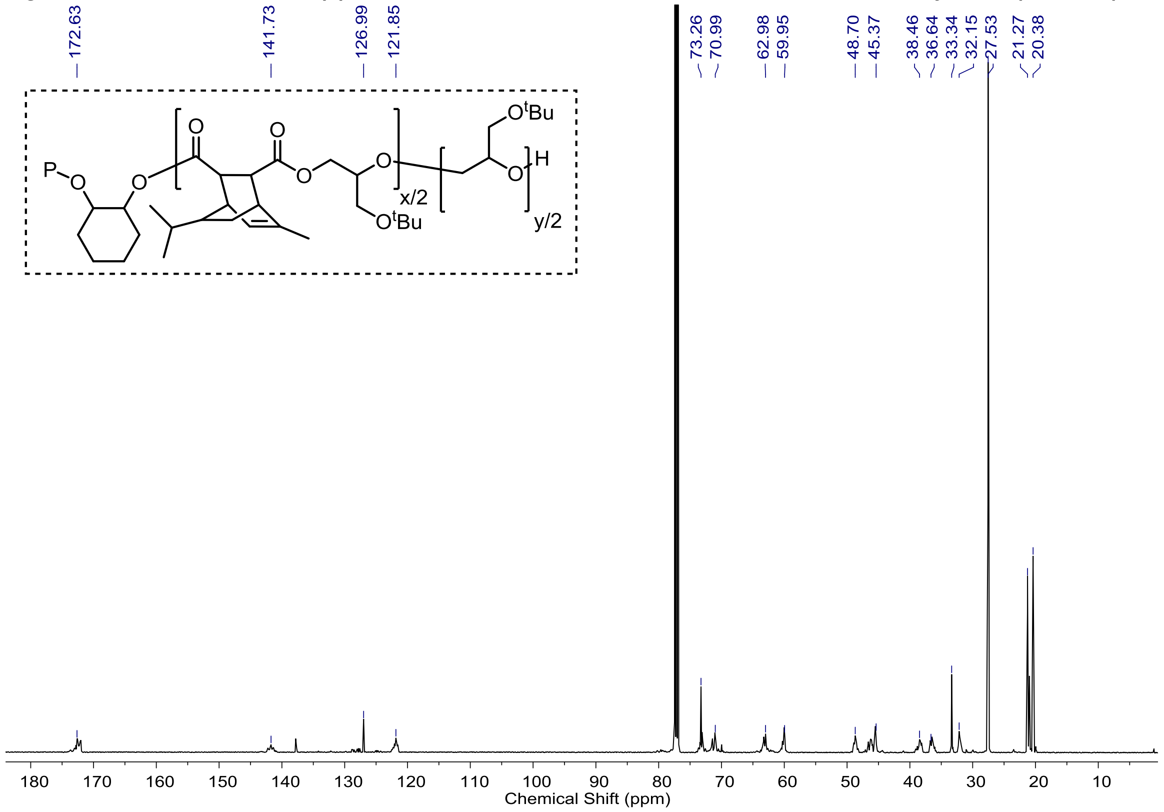

Supplementary Figure 69 – $^{13}C\{^{1}H\}$ NMR Spectrum for polymer obtained from tBGE/TCA1.

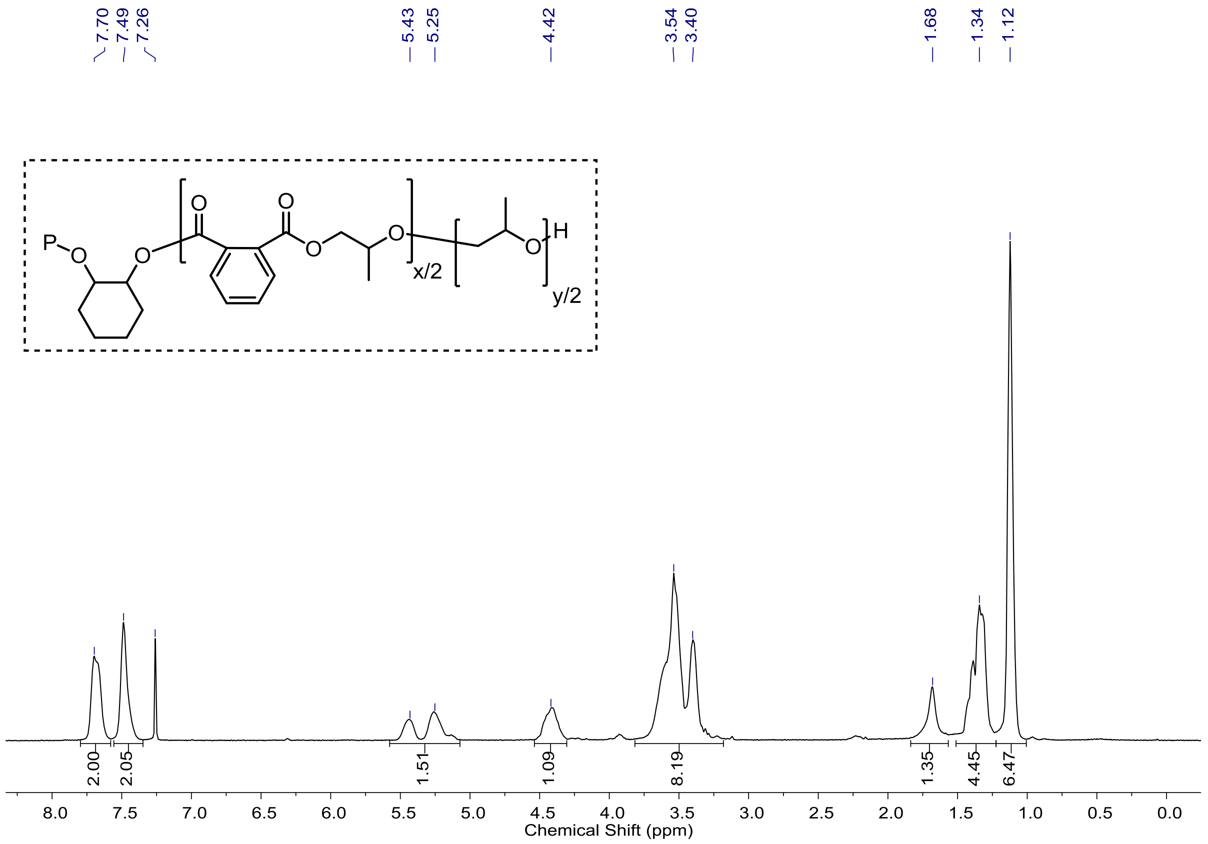

Supplementary Figure 70 – ^{1}H NMR Spectrum for polymer obtained from PA/PO.

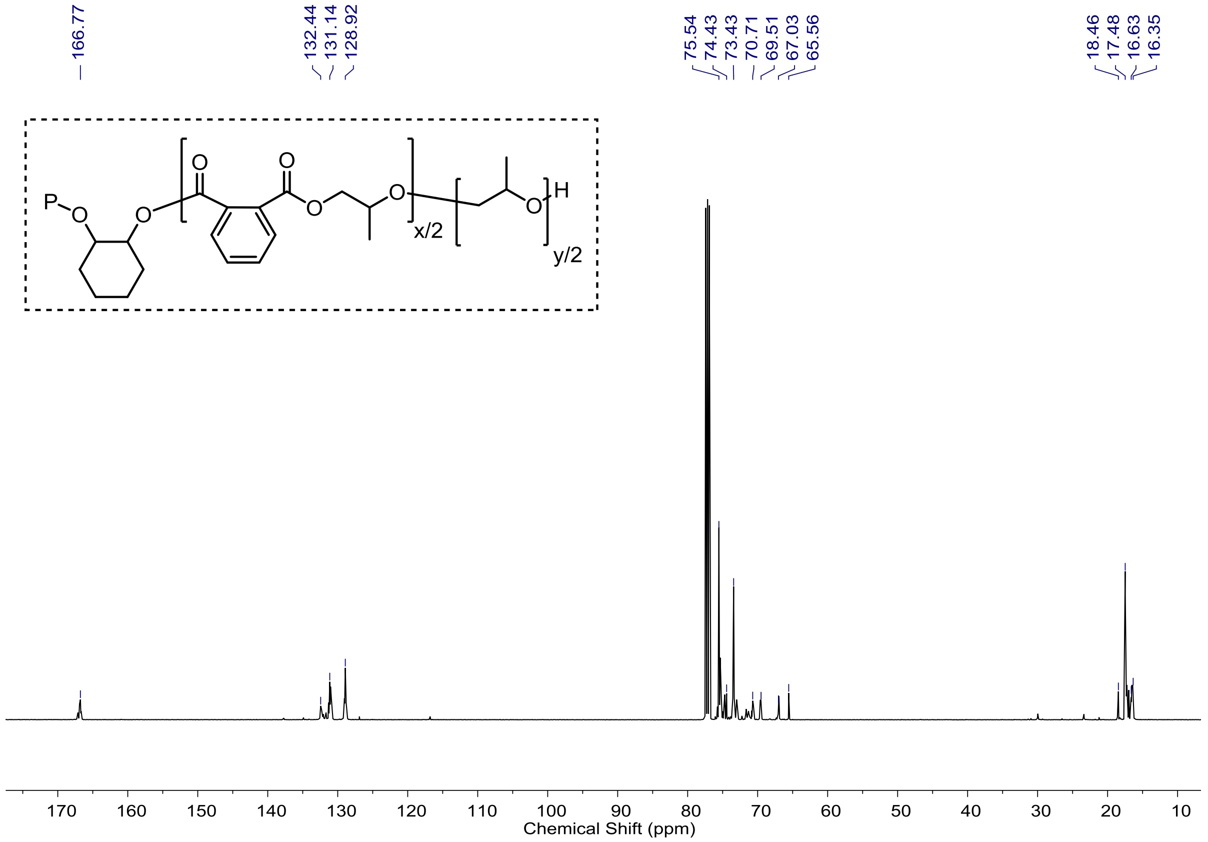

Supplementary Figure 71 – ^{13}C{^{1}H} NMR Spectrum for polymer obtained from PA/PO.

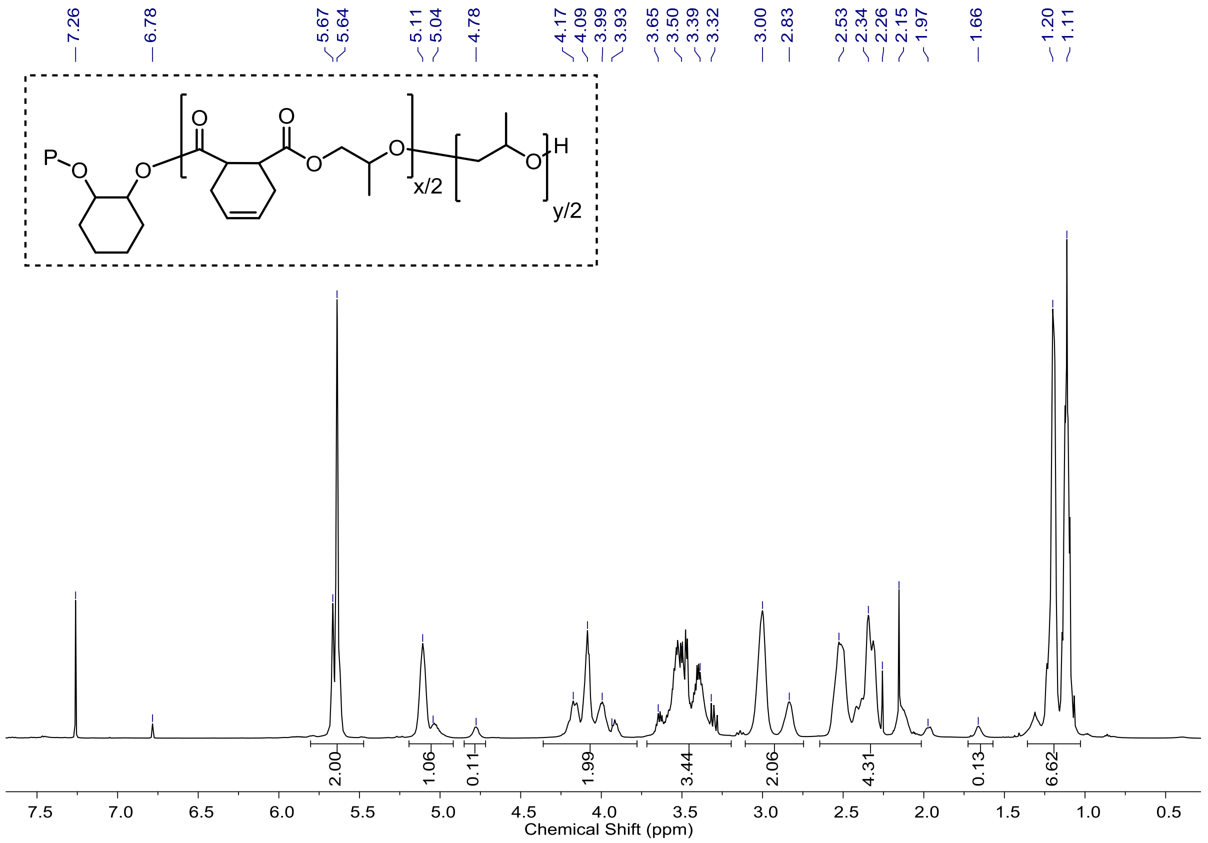

Supplementary Figure 72 – ^{1}H NMR Spectrum for polymer obtained from THPA/PO. The signals at 6.78 and 2.26 ppm are attributed to residual amounts of mesitylene (ca. 1%).

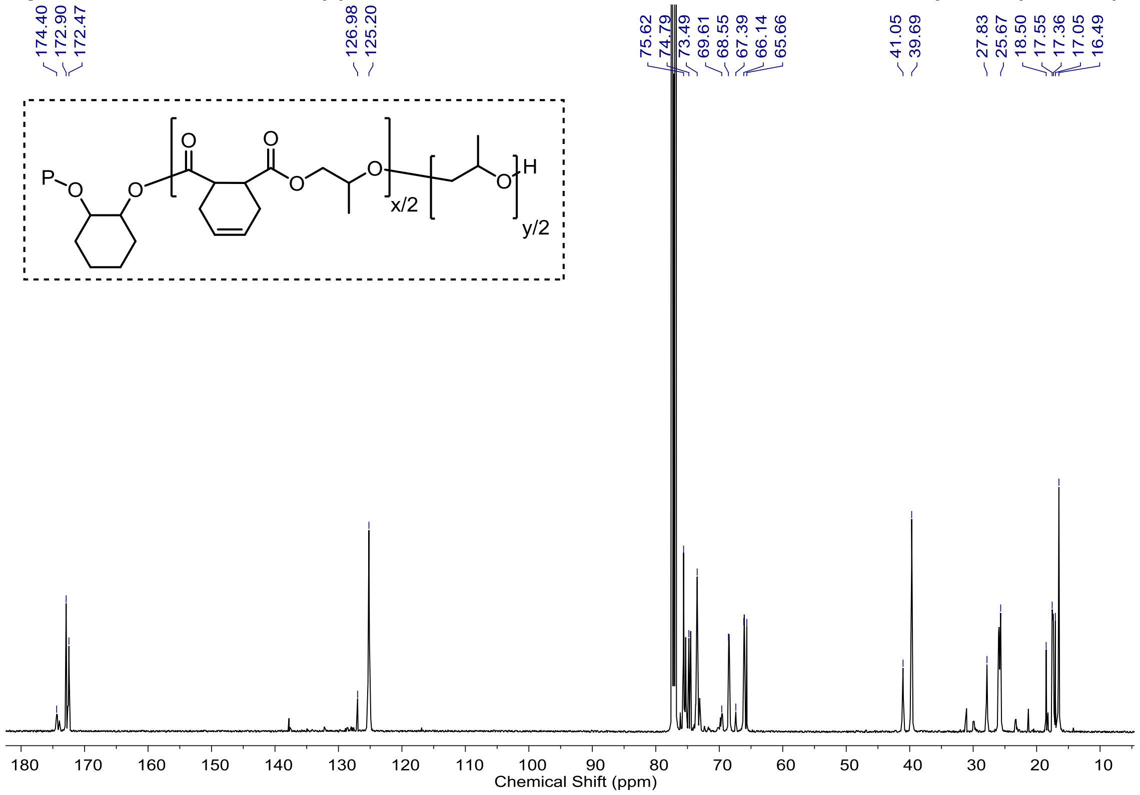

Supplementary Figure 73 – ^{13}C{^{1}H} NMR Spectrum for polymer obtained from THPA/PO.

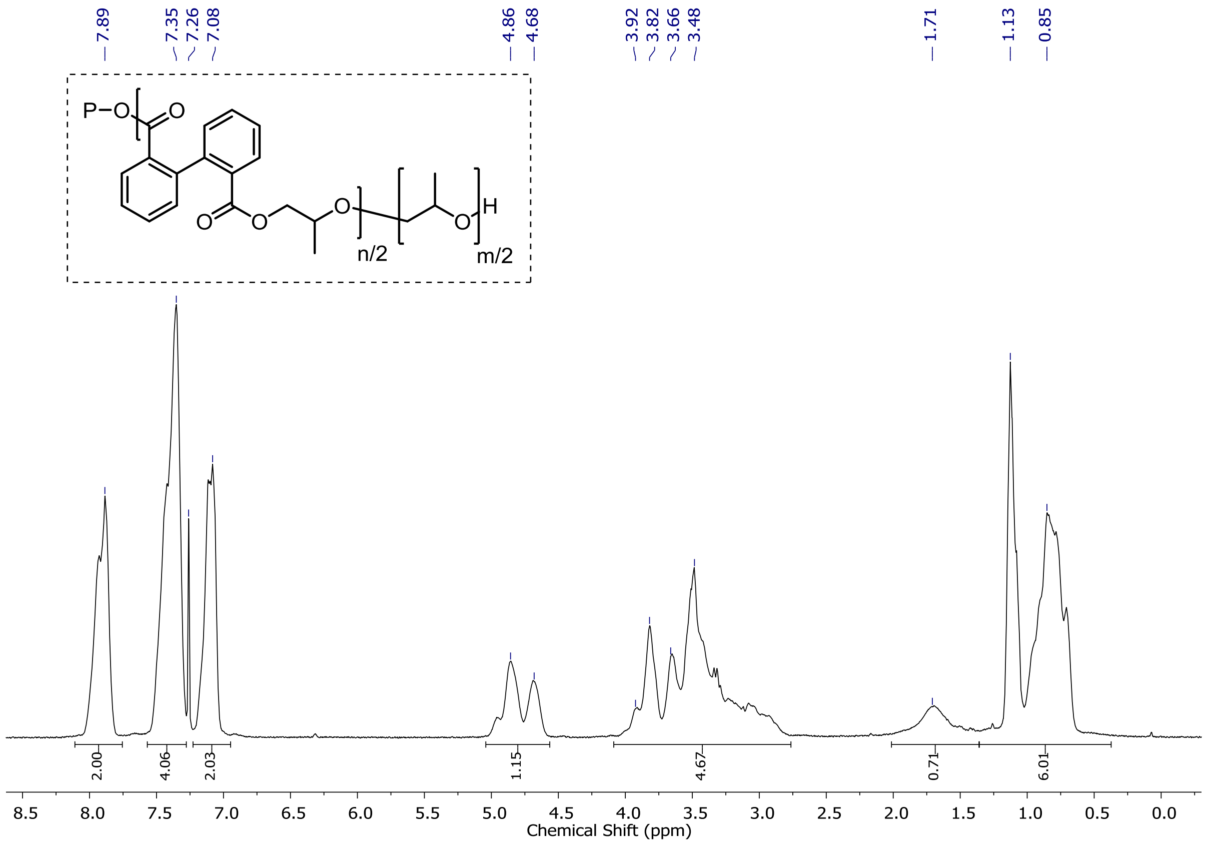

Supplementary Figure 74 – ^{1}H NMR Spectrum for polymer obtained from DPA/PO.

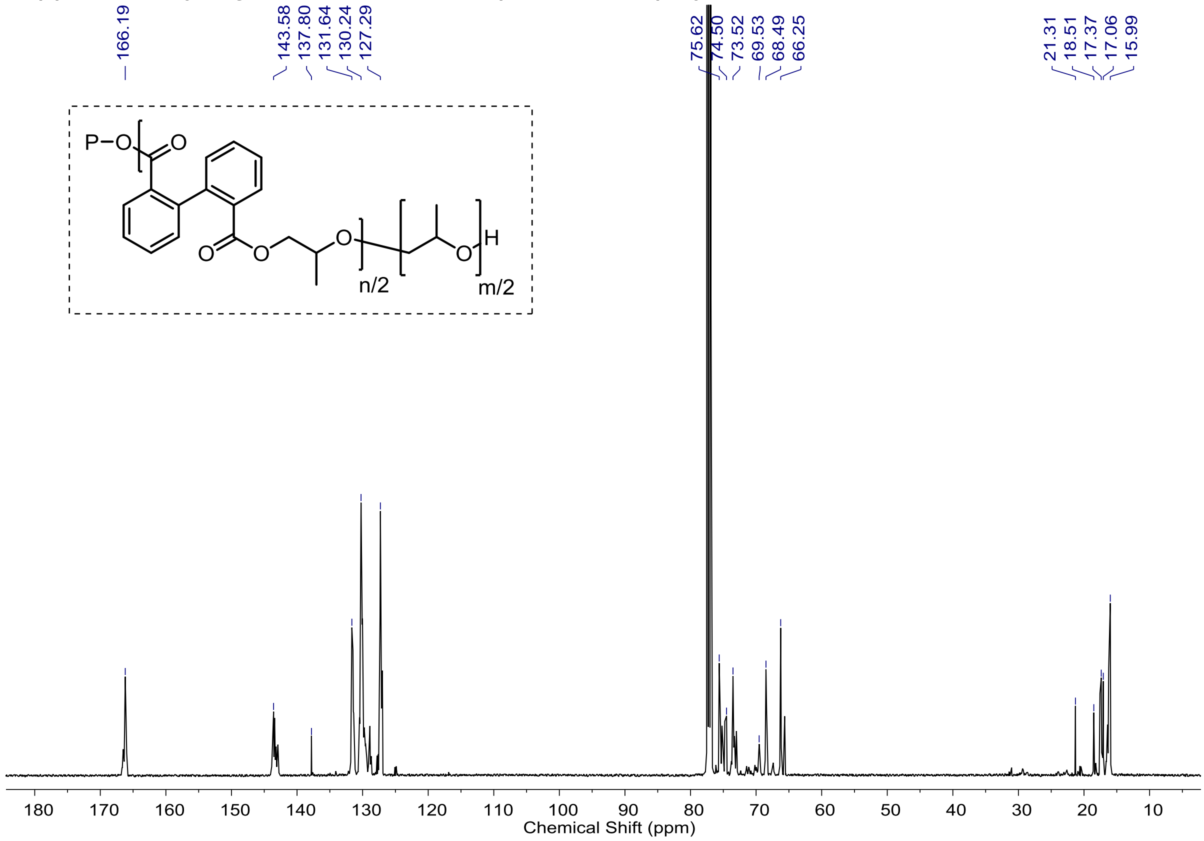

Supplementary Figure 75 – ^{1}H NMR Spectrum for polymer obtained from DPA/PO.

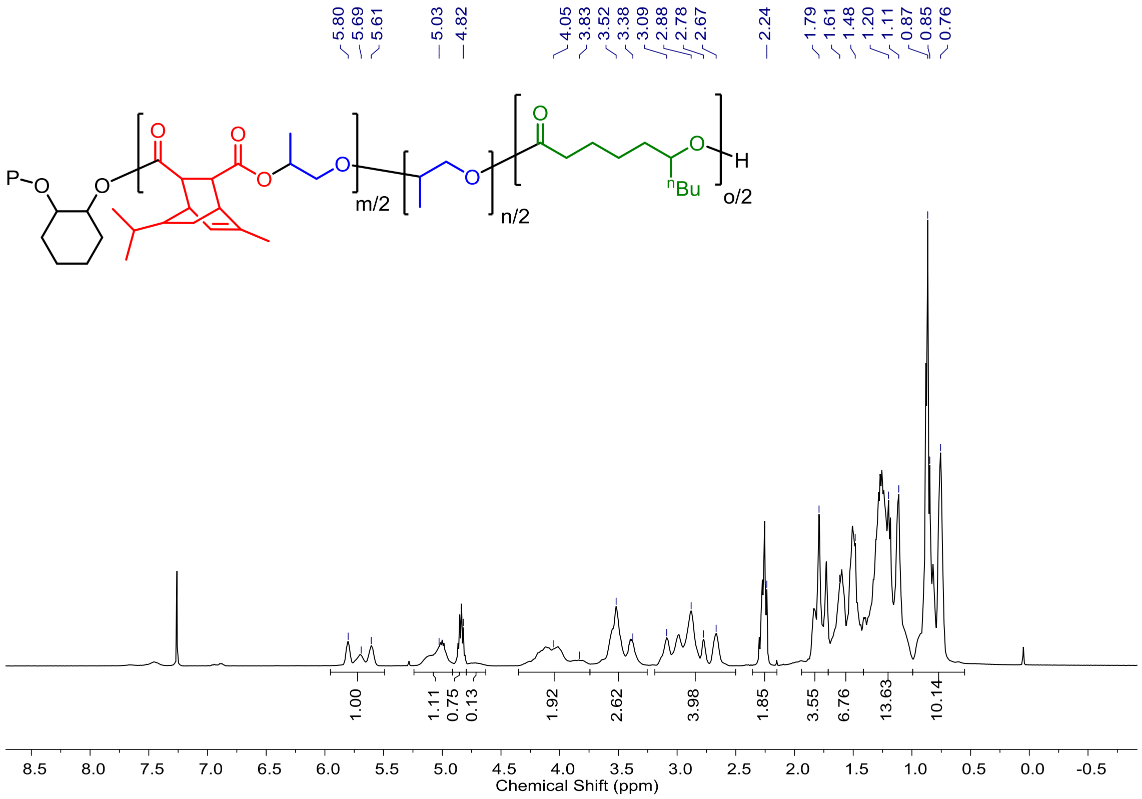

Supplementary Figure 76 – ^{1}H NMR Spectrum for polymer obtained from PO/TCA1/DL.

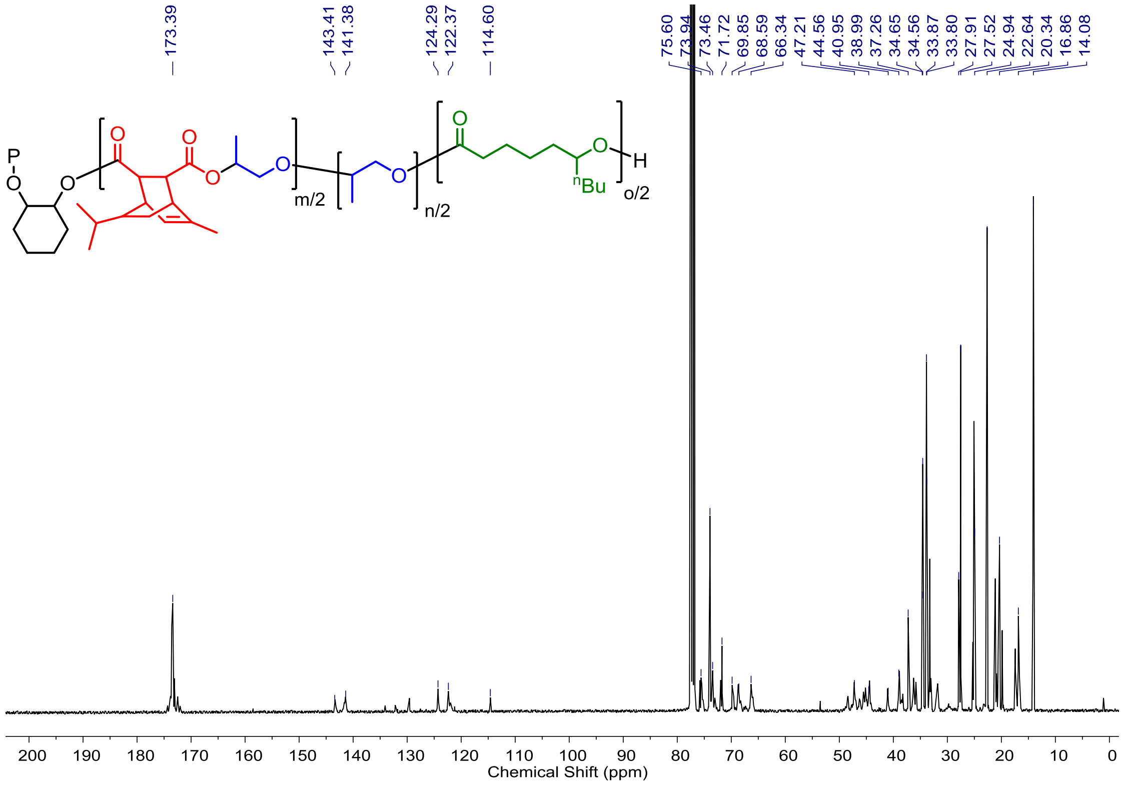

Supplementary Figure 77 – $^{13}C\{^{1}H\}$ NMR Spectrum for polymer obtained from PO/TCA1/DL.

Supplementary References

1 Fulmer, G. R., Miller, A. J. M., Sherden, N. H., Gottlieb, H. E., Nudelman, A., Stoltz, B. M., Bercaw, J. E. & Goldberg, K. I. NMR Chemical Shifts of Trace Impurities: Common Laboratory Solvents, Organics, and Gases in Deuterated Solvents Relevant to the Organometallic Chemist. *Organometallics* **29**, 2176-2179, (2010).

2 Keller, B. O., Sui, J., Young, A. B. & Whittal, R. M. Interferences and contaminants encountered in modern mass spectrometry. *Anal. Chim. Acta* **627**, 71-81, (2008).

3 Tong, H., Bell, D., Tabei, K. & Siegel, M. M. Automated data massaging, interpretation, and e-mailing modules for high throughput open access mass spectrometry. *J. Am. Soc. Mass Spectrom.* **10**, 1174-1187, (1999).

4 Antelmann, B., Chisholm, M. H., Iyer, S. S., Huffman, J. C., Navarro-Llobet, D., Pagel, M., Simonsick, W. J. & Zhong, W. Molecular Design of Single Site Catalyst Precursors for the Ring-Opening Polymerization of Cyclic Ethers and Esters. 2. Can Ring-Opening Polymerization of Propylene Oxide Occur by a Cis-Migratory Mechanism? *Macromolecules* **34**, 3159-3175, (2001).

5 Fineman, M. & Ross, S. D. Linear method for determining monomer reactivity ratios in copolymerization. *J. Polym. Sci.* **5**, 259-262, (1950).

6 Kazemi, N., Duever, T. A. & Penlidis, A. Reactivity Ratio Estimation from Cumulative Copolymer Composition Data. *Macromol. React. Eng.* **5**, 385-403, (2011).

7 Quan, S. M., Wei, J. & Diaconescu, P. L. Mechanistic Studies of Redox-Switchable Copolymerization of Lactide and Cyclohexene Oxide by a Zirconium Complex. *Organometallics* **36**, 4451-4457, (2017).
